# Effects of cannabidiol on anxiety- and depressive-like behaviors and cognition: a systematic review and meta-analysis of preclinical studies

**DOI:** 10.1038/s41380-026-03733-x

**Published:** 2026-06-30

**Authors:** Jeferson Jantsch, Fernanda Wickert, Gabriel de Farias Fraga, Luciano Stürmer de Fraga, Luz Elena Durán-Carabali, Renata Padilha Guedes

**Affiliations:** 1 Graduate Program in Biosciences, Federal University of Health Sciences of Porto Alegre (UFCSPA), Porto Alegre, Rio Grande do Sul Brazil; 2 Graduate Program in Physiology, Institute of Basic Health Sciences (ICBS), Federal University of Rio Grande do Sul (UFRGS), Porto Alegre, Rio Grande do Sul Brazil; 3 Department of Physiological Sciences, Federal University of Santa Catarina (UFSC), Florianópolis, Santa Catarina Brazil; 4 Graduate Program in Health Sciences, Federal University of Health Sciences of Porto Alegre (UFCSPA), Porto Alegre, Brazil

**Keywords:** Neuroscience, Diseases

## Abstract

**Background:**

Cannabidiol (CBD) has emerged as a promising neuroprotective compound, with potential benefits for cognitive and emotional behaviors. However, the consistency of its effects across preclinical models remains uncertain.

**Methods:**

We conducted a systematic review and meta-analysis of 123 animal studies assessing the impact of CBD on anxiety-like and depressive-like behaviors, as well as cognition.

**Results:**

CBD consistently reduced anxiety- and depressive-like behaviors, with moderate effect sizes observed in paradigms such as the elevated plus maze, novelty suppressed feeding, forced swim, sucrose preference, and tail suspension test. Cognitive effects were more heterogeneous: CBD improved performance in adverse, discriminatory, and working memory tasks, while effects were small or inconsistent in hippocampus-dependent spatial memory tests. Subgroup analyses revealed that CBD’s efficacy often depended on the type of behavioral test and the presence of underlying pathologies. Mechanistic evidence implicates serotonergic signaling (notably 5-HT1A receptors), endocannabinoid modulation, anti-inflammatory and neurotrophic pathways, as well as mitochondrial and synaptic plasticity processes, as contributors to these behavioral outcomes. Risk of bias assessment indicated moderate to high quality across studies, confirming the robustness of the findings.

**Conclusions:**

Overall, our results indicate that CBD reduces anxiety-like and depressive-like behaviors and improves cognitive performance across a variety of preclinical models. While these findings support its therapeutic potential and provide a rationale for guiding translational research, clinical evidence in humans is still limited and inconclusive, underscoring the need for further research.

## Introduction

Cannabidiol (CBD) is a non-psychoactive phytochemical compound derived from the *Cannabis sativa* plant, which has attracted increasing attention over recent decades due to its broad therapeutic potential [1]. Scientific and clinical interest in CBD increased following the pioneering work of Dr. Raphael Mechoulam, who first elucidated its chemical structure in 1964 and later advanced the field through innovative clinical studies on its therapeutic potential, particularly in epilepsy [2, 3]. Currently, the U.S. Food and Drug Administration (FDA) has approved the use of Epidiolex, a purified CBD formulation, for the treatment of seizures associated with Lennox-Gastaut syndrome, Dravet syndrome, and tuberous sclerosis complex [4].

Beyond epilepsy, in recent years CBD has been investigated in a variety of experimental models and disease conditions, showing potential benefits, from metabolic improvements in diabetic rats [5] to attenuation of inflammation and colitis symptoms in Dextran Sulfate Sodium (DSS)-induced models [6]. One of the most promising fields of research focuses on the central nervous system (CNS), where CBD has been shown to reduce anxiety- and depressive-like behaviors and to enhance cognitive performance [7]. Growing evidence indicates benefits effects of CBD in neurological disorders, mental health conditions, and behavioral outcomes, with minimal or no reported side effects [8]. This has reinforced interest in systematically exploring CBD’s potential role in cognition and behavior.

CBD acts through multiple mechanisms. Within the endocannabinoid system, it modulates endogenous cannabinoids such as anandamide (AEA) and 2-arachidonoylglycerol (2-AG) and also influences expression and activity of cannabinoid receptors CB1 and CB2 (although CBD is not a primary ligand of these receptors) [9, 10]. CB1 receptors are primarily found in neurons, whereas CB2 receptors are enriched in immune cells, including microglia, and both are expressed in brain circuits involved in mood regulation and cognition [9, 11]. Moreover, CBD interacts with multiple other molecular targets, such as the serotonin 5-HT1A receptor, transient receptor potential cation channels (TRP), G-protein-coupled receptor 55 (GPR55), peroxisome proliferator-activated receptor gamma (PPARγ), gamma-aminobutyric acid (GABA) receptors, and adenosine receptors. Detailed descriptions of these cellular and molecular targets of CBD can be found in recent reviews [9, 10, 12].

Although numerous preclinical studies have investigated the effects of CBD across behavioral and cognitive paradigms, the overall consistency and strength of these findings remain uncertain. To address this gap, we conducted a systematic review and meta-analysis of preclinical studies examining the behavioral and cognitive effects of CBD. We calculated effect sizes across studies and stratified them by behavioral task, with further subgroup analyses based on experimental model characteristics identified in the literature. This approach allows us to identify the most robust preclinical evidence available and highlights the most reliable findings to better guide future clinical research in humans.

## Methods

### PROSPERO registration

The protocol of this systematic review and meta-analysis was registered in the International Prospective Register of Systematic Reviews (PROSPERO; CRD42025617725 - January 2025) prior to its execution. The review was conducted in accordance with the Systematic Review Center for Laboratory animal Experimentation (SYRCLE) guidelines and the Preferred Reporting Items for Systematic Reviews and Meta-Analyses (PRISMA) statement (Supplementary PRISMA 2020 Checklist).

### Search

The literature search was structured according to the PICO framework: P (population): rodents; I (intervention): cannabidiol (CBD); C (comparator): vehicle treatment; O (outcome): behaviors related to anxiety, depression, and memory.

The following Boolean operator chain was applied: ((((rats) OR (rat) OR (mice) OR (mouse) OR (rodents) OR (murine) OR (animals) OR (animal)) AND ((Cannabidiol) OR (CBD)) AND ((anxiety) OR (anxiety-like) OR (anxious) OR (anxious-like) OR (depression) OR (depression-like) OR (depressive) OR (depressive-like) OR (anhedonia) OR (psychotic disorders) OR (mood) OR (mood disorders) OR (mental health) OR (cognitive decline) OR (mild cognitive impairment) OR (MCI) OR (neurodegenerative disease) OR (neurodegeneration) OR (Alzheimer’s disease) OR (Alzheimer) OR (Parkinson) OR (Parkinson’s disease) OR (dementia) OR (aging) OR (age-related) OR (aging related impairments) OR (aged) OR (stress-induced) OR (cognitive impairment) OR (cognitive decline) OR (neurobehavioral disorders) OR (memory impairment) OR (memory loss) OR (behavior) OR (memory))) NOT (human)) NOT (review).

The search was performed in PubMed, Scopus, and Web of Science databases, covering the period from January 1995 to November 1, 2024. Only articles published in English were considered.

### Inclusion and exclusion criteria

Eligible studies included experimental animal research involving rodents in which a neuropsychiatric or mood-related state was either (1) pre-established through induced disease or stress models, or (2) elicited during validated behavioral paradigms without prior pathological induction. This approach allowed the inclusion of studies where the behavioral test was used to induce anxiety- or depressive-like related responses as experimental models of these conditions. To be included, studies were required to assess at least one of the following outcomes: a) anxiety-related behaviors: elevated plus maze (with elevated zero maze considered equivalent), open field, light-dark box, and novelty suppressed feeding; b) depressive-related behaviors: forced swim test, sucrose/ saccharin preference test, tail suspension test; c) Cognition-related behaviors: Barnes maze, contextual fear conditioning, cued fear conditioning, inhibitory avoidance, Morris water maze, object location, object recognition, social memory, and Y maze. Only full-text and peer-reviewed studies published in English were considered.

Exclusion criteria, applied in order of priority, were: 1) not an animal study; 2) not published in English; 3) not involving models of neuropsychiatric, neurodegenerative, or mood disorders (via experimental manipulation or behavioral paradigm); 4) absence of a vehicle-treated group exposed to the same experimental model or induced condition as the CBD-treated group; 5) in vitro or cell culture studies only; 6) co-treatment with other cannabinoids or drugs; 7) use of CBD-rich extracts instead of isolated CBD; 8) if behavioral tests were performed but data related to anxiety-, depressive-, or cognition-associated outcomes were not reported; and 9) behavioral tests unrelated to anxiety-, depressive-, or cognition-associated behaviors.

### Article selection

Articles were imported into Rayyan software [13], where duplicates were removed. Titles and abstracts were screened, and studies were included only if approved by two independent reviewers (JJ and FW). In cases of disagreement, a third reviewer (GFF) examined the articles. Studies with two positive assessments were then reviewed in full to confirm eligibility. Agreement in study inclusion was assessed using Kappa test, with values interpreted as ≤0 no agreement, >0.20 none to slight, 0.21–0.40 fair, 0.41–0.60 moderate, 0.61–0.80 substantial, and 0.81–1.00 almost perfect [14].

### Study quality and risk of bias assessment

Methodological quality and risk of bias were assessed using two tools: (i) a scoring system adapted from the Collaborative Approach to Meta-Analysis and Review of Animal Data from Experimental Studies (CAMARADES) guidelines, and (ii) the 10-item SYRCLE Risk of Bias (RoB) tool for preclinical studies [15]. A combined score from both tools was used to categorize studies as very high quality (≥16), high (11–15), moderate (6–10), or low (≤5).

All assessments were independently performed by two reviewers (FW and GFF) with disagreements resolved by a third reviewer (JJ). Funnel plots were used to visualize the distribution of effect sizes. Asymmetry was detected using Egger’s regression test, and in the case of publication bias, the trim-and-fill method was applied to estimate its impact and adjust pooled effect size [16].

### Data extraction

For each behavioral test, data were extracted following the order of parameters listed in Supplementary Table 1; if a parameter was not available, the next listed outcome was selected. For each outcome, data were extracted as mean and standard error of the mean (SEM) or standard deviation (SD), or as median with interquartile range, minimum, and maximum values. Whenever possible, numerical values were collected directly from tables or text; when available only in graphical form, values were extracted using the WebPlotDigitizer (version 4.8). Studies were assigned to syntheses according to the behavioral outcomes they reported. As different studies evaluated different behavioral domains, studies were included in each outcome-specific synthesis whenever data for the corresponding behavioral endpoint were available. All included studies contributed to at least one synthesis.

Additional information recorded included first author, year of publication, species, sex, age, treatment duration, interval between the last administered dose and behavioral testing, and the disease model (if applicable). Relevant study characteristics were summarized in a descriptive table.

### Data analysis

All analyses were conducted in R (version 4.5.0, 2025.04.11) using the packages *esc* [17], *meta* [18], *dmetar* [19], and *metafor* [20]. Effect sizes were calculated using Hedges’ g standardized mean difference (SMD), determined as the difference between the CBD and vehicle groups. Effect size magnitude was interpreted according to standard accepted thresholds: small (0.2–0.5), moderate (0.5–0.8), large (>0.8).

The direction of SMD values was standardized so that negative values indicate a protective effect of CBD, as stated in the captions of each figure. Forest plots were generated for each behavioral outcome to visually display the pooled effect estimates and their corresponding 95% confidence intervals, including all eligible studies. Subgroup analyses were conducted based on the behavioral paradigm, experimental model, sex, and dose. For the dose analysis, we first stratified the data by route of administration (intraperitoneal and oral), as other routes did not reach the minimum number of comparable outcomes required for separate meta-analyses. Within each route, doses were further categorized as low (≤10 mg/kg), moderate (>10 to <30 mg/kg), and high (≥30 mg/kg). These classifications were based on cut-off values derived from the dose distribution in our dataset. Subgroup analyses were performed only when at least two studies reported comparable measures.

Heterogeneity was evaluated using the I² statistic, Cochran’s Q test, and prediction intervals. I² values were interpreted as low (0–25%), moderate (25–50%), substantial > 50% [21]. Potential outliers were identified with the *find.outliers* function and excluded from further analyses. Additionally, we assessed the influence of individual studies on the overall meta-analytic results using influence diagnostics. This included graphical inspection of standardized residuals, Cook’s distance, DFFITS, hat values, covariance ratios, and other related parameters. Study was considered influential only if flagged across all diagnostic criteria [22].

### Moderator assessment

A random-effects meta-regression was conducted to assess the impact of various moderators on the overall effect size and the variability among studies. The moderators included were route of administration (intraperitoneal, intravenous, vaporized, CNS injection, oral, subcutaneous), sex (female, male, mixed), number of administrations (single, repeated), disease model, and age at the start of the experiment.

## Results

### Studies description

**Fig. 1:**
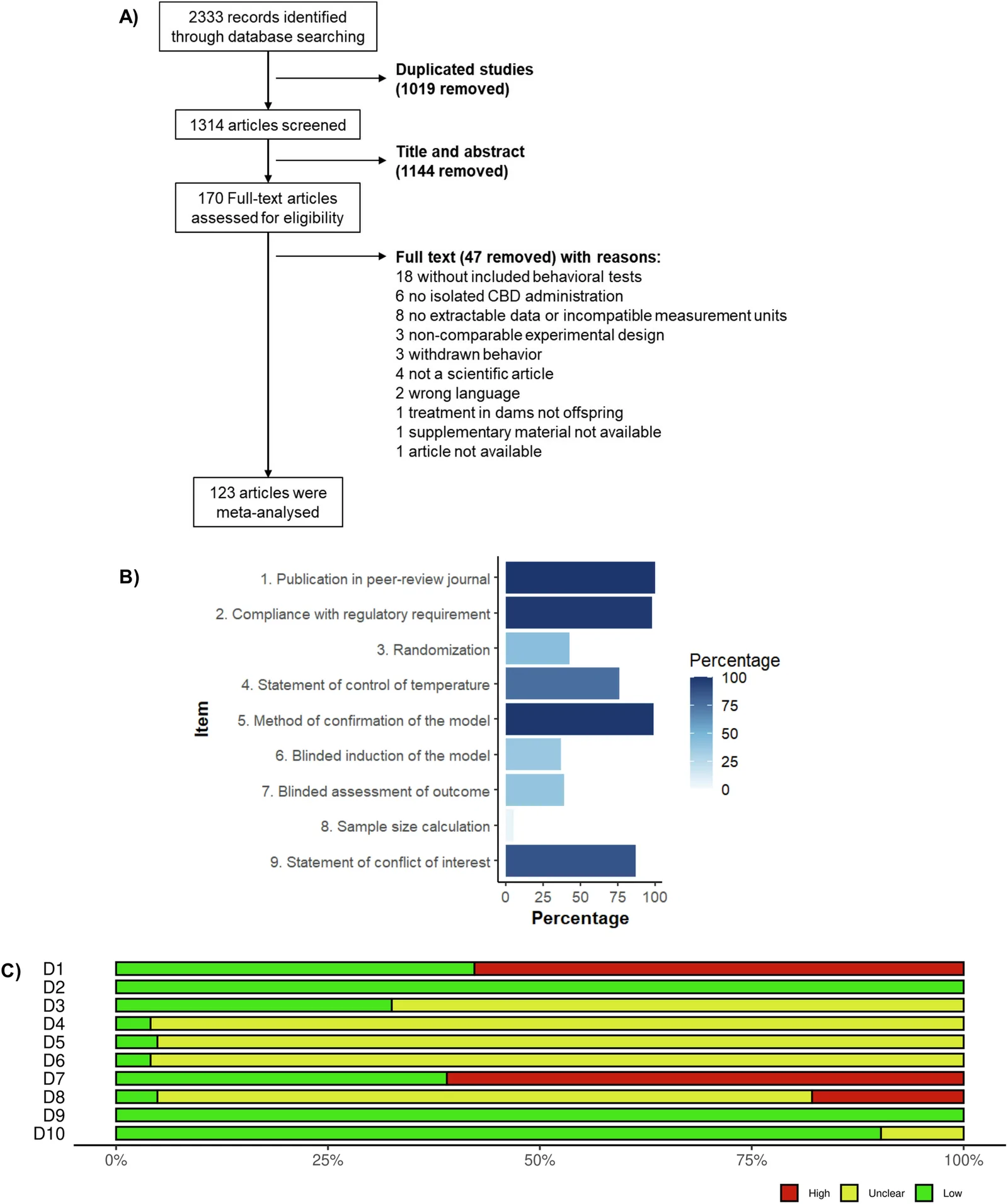
Study selection and quality assessment. **A** Flowchart of the article selection process from three databases: PubMed, Scopus, and Web of Science. Of the total articles identified using the search terms, 1314 remained after removing duplicates. A total of 170 articles were assessed, and 123 were ultimately included. **B** Study quality was evaluated using CAMARADES, and **C** the SYRCLE’s RoB tool was applied to evaluate the risk of bias. Results are expressed as the percentage of studies reporting each category. D1 = random sequence generation; D2 = baseline characteristics; D3 = allocation concealment; D4 = random housing; D5 = blinding; D6 = random outcome assessment; D7 = blinding of outcome assessment; D8 = incomplete outcome data; D9 = selective reporting; D10 = other bias.

The database search retrieved 2333 records. After removing duplicates, a total of 1314 studies were selected for screening. Following title and abstract reading, 1144 were excluded (Fig. 1A). During full-text assessment, 47 additional articles were excluded: one because the full article could not be obtained due to insufficient contact information for the corresponding author, and another because the data of interest were only available in the supplementary material, which was not provided after contacting the authors. The remaining 45 articles were excluded for the following reasons: 18 did not included behavioral tests, 6 did not administered CBD in isolation, 8 lacked extractable data or used incompatible measurement units, 3 had non-comparable experimental designs, 3 assessed withdrawal behavior, 4 were not scientific articles, 2 were not written in English, and 1 applied treatment to dams rather than offspring. In total, 123 studies were included. The study population across the included articles comprised rodents, specifically rats, mice, and hamsters (details of the studies and behavioral outcomes extracted from each study are provided in Supplementary Table 2 and Supplementary Table 3). Agreement in study inclusion was assessed using Cohen’s Kappa, resulting in Kappa = 0.602 (p < 0.001), indicating moderate agreement. The overall percent agreement between reviewers was 90.3%.

### Study quality and risk of bias assessment

The assessment of the articles using the adapted CAMARADES tool (Fig. 1B) showed that the least frequently reported items were sample size calculation (5%), blinding of model induction (37%), blinding in outcome assessment (39%), and statements on randomization (43%). All evaluated studies were published in peer-reviewed journals. Moreover, 99% reported confirmation of model induction, 98% complied with regulatory requirements for the use of animal models, 87% explicitly declared no conflicts of interest, and 76% included statements on temperature control in animal housing. A summary of the SYRCLE’s Risk of Bias (RoB) tool (Fig. 1C and Supplementary Fig. S1) indicates that the main sources of potential bias were related to inadequate reporting of randomization, blinding, and incomplete outcome data. Among the studies analyzed, 67 were classified as high quality and 56 as moderate quality. No study was categorized as very high quality or poor quality.

### Anxiety-like behaviors

**Fig. 2:**
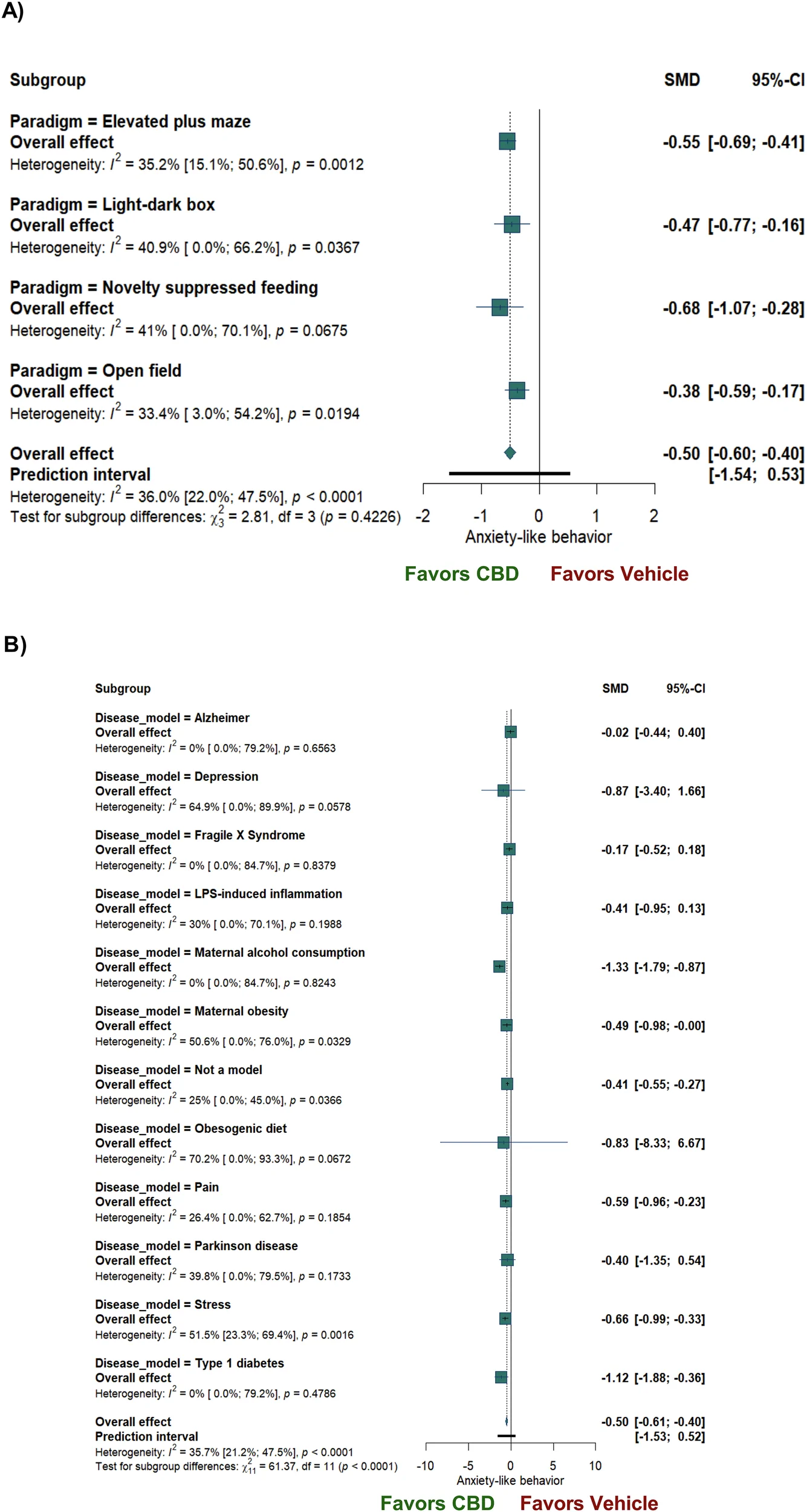
Forest plots of meta-analyses evaluating anxiety-like behaviors. **A** Forest plot of the meta-analysis comparing the performance of animals treated with CBD versus vehicle across the evaluated paradigms related to anxiety-like behavior. **B** Forest plot of the meta-analysis comparing the performance of animals treated with CBD versus vehicle subgrouped by disease model independent of the behavioral test. Outliers and influential results were removed prior to analysis. Negative values indicate a favorable effect of CBD, suggesting a potential anxiolytic effect across all tests. CBD, cannabidiol; CI, confidence interval; DF, degrees of freedom; SMD, standardized mean difference.

To assess anxiety-like behavior, we included the most commonly used paradigms in the literature: the elevated plus maze, open field test, light/dark box, and novelty-suppressed feeding test. A meta-analysis of 200 results from 75 studies showed a moderate anxiolytic effect of CBD compared to vehicle-treated groups (overall effect (SMD): −0.64; 95% CI = [−0.79; −0.48]; p < 0.0001; prediction interval = [−2.80 to 1.53]; heterogeneity: I² = 71.9%, 95% CI = [67.6%; 75.6%]; Q = 707.93, p < 0.0001) (Supplementary Fig. S2, **including all studies**). Forty-four results were identified as outliers; after their removal, the anxiolytic effect of CBD remained moderate and statistically significant (overall effect (SMD): −0.50; 95% CI = [−0.60; −0.40]; p < 0.0001; prediction interval = [−1.54; 0.53]; heterogeneity: I² = 36.0%, 95% CI = [22.0%; 47.5%]; Q = 242.23, p < 0.0001, k = 156) (Fig. 2A, **excluding outliers**). These results indicate that CBD treatment has anxiolytic action.

When analyzed by behavioral paradigm (Fig. 2A; Supplementary Fig. S2), moderate effect was observed in the elevated plus maze (SMD: −0.55; 95% CI = [−0.69; −0.41]; I² = 35.2%; k = 83) and the novelty suppressed feeding test (SMD: −0.68; 95% CI = [−1.07; −0.28]; I² = 41.0%; k = 12). Smaller, yet still significant, effects were found in the open field test (SMD: −0.38; 95% CI = [−0.59; −0.17]; I² = 33.4%; k = 43) and the light-dark box (SMD: −0.47; 95% CI = [−0.77; −0.16]; I² = 40.9%; k = 18).

Subgroup analysis by sex showed significant small to moderate effects in both males (SMD: −0.50; 95% CI = [−0.62; −0.39]; I² = 35.1%; k = 116) and females (SMD: −0.52; 95% CI = [−0.78; −0.26]; I² = 40.9%; k = 29). Results including both sexes did not reach significance (SMD: −0.34; 95% CI = [−0.87; 0.17]; I² = 36.4%; k = 8), and studies in which sex was not reported showed a highly imprecise estimate (SMD: −1.13; 95% CI = [−8.96; 6.70]; I² = 59.2%; k = 2), possibly due to the small number of studies. Overall, effect sizes were comparable between males and females regarding anxiety-like outcomes (Supplementary Fig. S3A, **including all studies** and Fig. S3B, **excluding outliers**).

Subgroup analyses based on species revealed similar effects of CBD in mice (SMD: −0.50; 95% CI = [−0.64; −0.35]; I² = 40.2%; k = 80 and rats (SMD: −0.51; 95% CI = [−0.66; −0.37]; I² = 31.8%; k = 76), with no evidence of significant differences between species (Supplementary Fig. S4A, **including all studies** and Fig. S4B, **excluding outliers**).

To analyze dose-dependent effects, we focused on intraperitoneal and oral regimens, as these are the most commonly used routes of administration and provide a sufficient number of studies for comparison. Regarding intraperitoneal administration, all dose categories showed statistically significant effect (low: SMD: −0.48; 95% CI = [−0.64; −0.33]; I² = 38.2%; k = 69; moderate: SMD: −0.47; 95% CI = [−0.76; −0.18]; I² = 18.4%; k = 13; high: SMD: −0.56; 95% CI = [−0.76; −0.37]; I² = 35.2%; k = 47), with no differences observed between groups (Q = 0.52, p = 0.77) (Supplementary Fig. S5A, **including all studies** and Fig. S5B, **excluding outliers**). In contrast, for oral administration, none of the dose categories reached statistical significance (low: SMD: −0.11; 95% CI = [−4.40; 4.17]; I² = 0.0%; k = 2; moderate: SMD: −0.13; 95% CI = [−0.28; 0.02]; I² = 0.0%; k = 3; high: SMD: −0.48; 95% CI = [−1.07; 0.10]; I² = 56.3%; k = 9), which may be related to the limited number of studies included in each subgroup. Additionally, no significant differences were observed between dose groups (Q = 1.87, p = 0.39) (Supplementary Fig. S5C, **including all studies** and Fig. S5D, **excluding outliers**).

We then subgrouped the analysis by disease model (Fig. 2B, **excluding outliers**; Supplementary Fig. S6, **including all studies**), and moderate to large effects were observed in models of maternal alcohol consumption (SMD: −1.33; 95% CI = [−1.79; −0.87]; I² = 0.0%; k = 4), type 1 diabetes (SMD: −1.12; 95% CI = [−1.88; −0.36]; I² = 0.0%; k = 5), stress (SMD: −0.66; 95% CI = [−0.99; −0.33]; I² = 51.5%; k = 25), and pain (SMD: −0.59; 95% CI = [−0.96; −0.23]; I² = 26.4%; k = 12). Smaller, yet still significant, effects were found in maternal obesity (SMD: −0.49; 95% CI = [−0.98; −0.0001]; I² = 50.6%; k = 10) and in models not classified under a specific disease category (SMD: −0.41; 95% CI = [−0.55; −0.27]; I² = 25.0%; k = 67). Effects were small and not significant in Alzheimer’s disease (SMD: −0.02; 95% CI = [−0.44; 0.40]; I² = 0.0%; k = 5), Fragile X Syndrome (SMD: −0.17; 95% CI = [−0.52; 0.18]; I² = 0.0%; k = 4), LPS-induced inflammation (SMD: −0.41; 95% CI = [−0.95; 0.13]; I² = 30.0%; k = 7), Parkinson’s disease (SMD: −0.40; 95% CI = [−1.35; 0.54]; I² = 39.8%; k = 4), model of depression (SMD: −0.87; 95% CI = [−3.40; 1.66]; I² = 64.9%; k = 3), and obesogenic diet models (SMD: −0.83; 95% CI = [−8.33; 6.67]; I² = 70.2%; k = 2).

We then conducted an exploratory analysis to examine the different experimental models in more detail, specifically within each behavioral test (Supplementary Fig. S7, **excluding outliers**; Supplementary Fig. S8**, including all studies**). A meta-analysis was conducted when two or more studies were available for each category. In the elevated plus maze, this test showed the largest number of models with differences. Moderate to large effects were observed in stress models (SMD: −0.78; 95% CI = [−1.24; −0.32]; I² = 45.8%; k = 14) and type 1 diabetes models (SMD: −1.12; 95% CI = [−1.88; −0.36]; I² = 0.0%; k = 5). Smaller effects were found in studies not classified under a specific disease (SMD: −0.51; 95% CI = [−0.72; −0.31]; I² = 31.6%; k = 37). Non-significant effects were observed in the other models included (Alzheimer’s disease: SMD = −0.05; 95% CI = [−0.63; 0.53]; I² = 0.0%; k = 4; pain: SMD = −0.56; 95% CI = [−1.30; 0.18]; I² = 34.9%; k = 5; maternal obesity: SMD = −0.50; 95% CI = [−1.04; 0.03]; I² = 43.6%; k = 8; Fragile X Syndrome: SMD = −0.30; 95% CI = [−2.65; 2.05]; I² = 0.0%; k = 2; LPS-induced inflammation: SMD = −0.47; 95% CI = [−1.26; 0.31]; I² = 43.2%; k = 5).

In the open field test, a large effect was observed in pain models (SMD: −0.99; 95% CI = [−1.82; −0.16]; I² = 0.0%; k = 4). Only non-significant effects were found in the other included studies (not classified as a disease: SMD = −0.21; 95% CI = [−0.42; 0.00]; I² = 0.0%; k = 18; stress: SMD = −0.57; 95% CI = [−1.72; 0.58]; I² = 67.9%; k = 6; LPS-induced inflammation: SMD = −0.24; 95% CI = [−5.55; 5.08]; I² = 23.8%; k = 2; model of depression: SMD = −1.36; 95% CI = [−9.97; 7.26]; I² = 53.8%; k = 2; Parkinson’s disease: SMD = 0.01; 95% CI = [−4.81; 4.83]; I² = 30.8%; k = 2; maternal obesity: SMD = −0.43; 95% CI = [−10.87; 10.00]; I² = 82.5%; k = 2); Fragile X Syndrome: SMD = −0.04; 95% CI = [−0.92; 0.84]; I² = 0.0%; k = 2).

Only non-significant effects were seen in the included models in the light-dark box (stress: SMD = −1.14; 95% CI = [−4.14; 1.86]; I² = 0.0%; k = 2; maternal alcohol consumption: SMD = −1.31; 95% CI = [−4.68; 2.06]; I² = 0.0%; k = 2; studies not classified as a disease: SMD = −0.36; 95% CI = [−0.75; 0.03]; I² = 18.8%; k = 10; pain: SMD = −0.02; 95% CI = [−1.82; 1.78]; I² = 0.0%; k = 2). For the novelty suppressed feeding, only non-significant effects were also detected: maternal alcohol consumption (SMD: −1.36; 95% CI = [−4.13; 1.40]; I² = 0.0%; k = 2); pain (SMD: −0.83; 95% CI = [−2.44; 0.77]; I² = 0.0%; k = 2); studies not classified as a disease (SMD: −0.81; 95% CI = [−12.13; 10.50]; I² = 83.1%; k = 2), obesogenic diet (SMD: −0.83; 95% CI = [−8.33; 6.67]; I² = 70.2%; k = 2), and stress (SMD: −0.12; 95% CI = [−0.45; 0.21]; I² = 0.0%; k = 3). These results suggest that CBD exerts important effects that vary depending on the disease model and the specific behavioral test. However, the presence of non-significant findings in several subgroups may reflect the small number of studies included for some models and the high heterogeneity observed across studies.

Egger’s test revealed significant asymmetry, consistent with publication bias (intercept = −4.23, 95% CI = [−5.35; −3.12], t = −7.46, p < 0.0001; Supplementary Fig. S9). After outlier removal and applying the trim-and-fill method, 31 results were added, and the overall effect size changed from moderate −0.50 to a small effect size −0.28 (95% CI = [−0.40; −0.16]; p < 0.0001). This finding indicates that while CBD shows consistent anxiolytic properties, the strength of this effect should be interpreted with caution given the possible influence of selective publication. None of the parameters evaluated as moderators were found to be significant in the meta-regression.

### Depressive-like behaviors

**Fig. 3:**
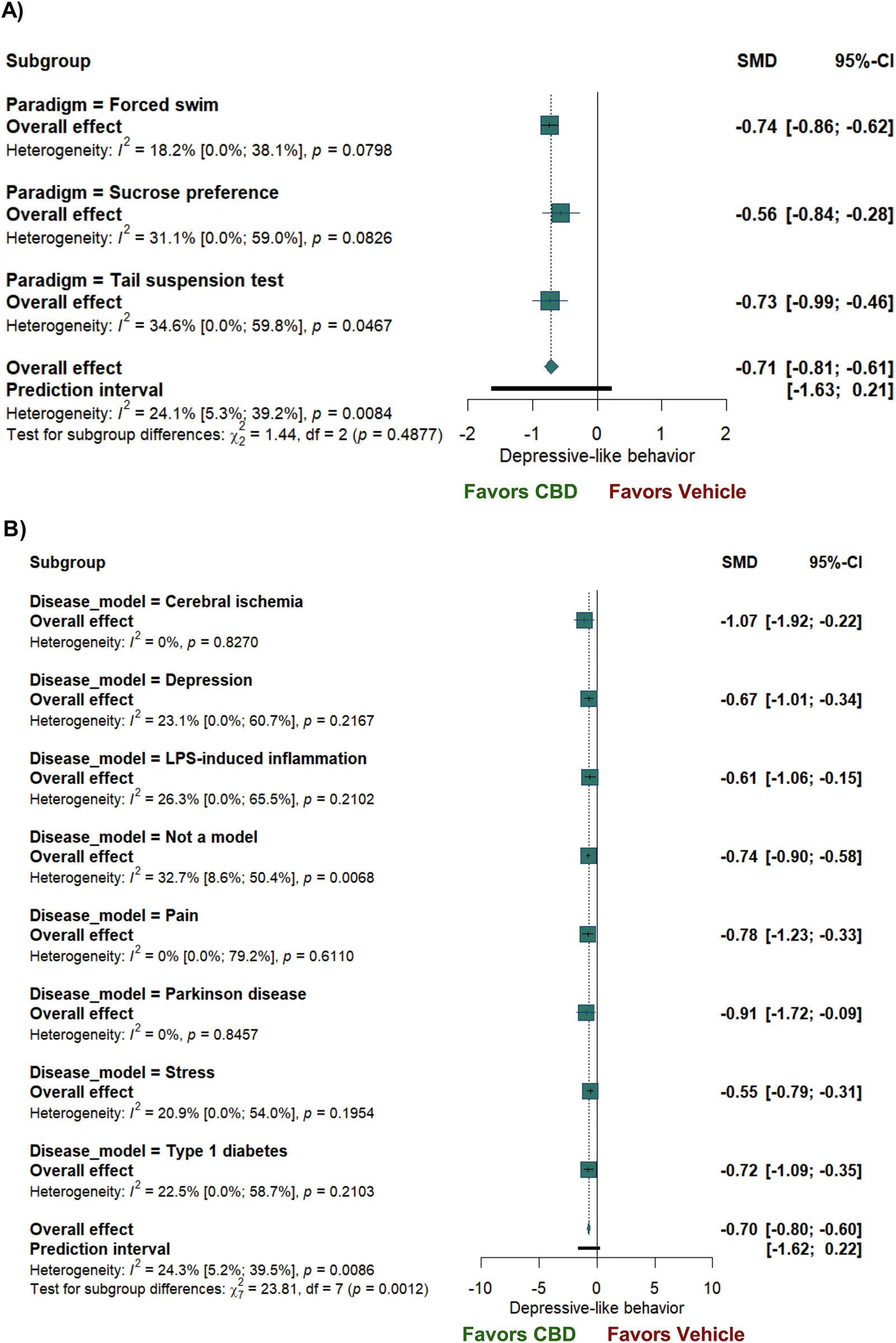
Forest plots of meta-analyses assessing depressive-like behaviors. **A** Forest plot of the meta-analysis comparing the performance of animals treated with CBD versus vehicle across the evaluated paradigms related to depressive-like behavior. **B** Forest plot of the meta-analysis comparing the performance of animals treated with CBD versus vehicle subgrouped by disease model independent of the depressive-like behavior paradigm. Outliers and influential results were removed prior to analysis. Negative values indicate a favorable effect of CBD, suggesting a potential antidepressant effect across all tests. CBD, cannabidiol; CI, confidence interval; DF, degrees of freedom; SMD, standardized mean difference.

To assess depressive-like behavior, we included the forced swim test, tail suspension test, and sucrose/saccharine preference test. A meta-analysis of 160 results from 46 studies showed a moderate and statistically significant effect of CBD in reducing depressive-like behaviors compared to vehicle-treated groups (overall effect (SMD): −0.70; 95% CI = [−0.85; −0.55]; p < 0.0001; prediction interval = [−2.45 to 1.06]; heterogeneity: I² = 69.4%, 95% CI = [64.1%; 74.0%]; Q = 519.85, p < 0.0001) (Supplementary Fig. S10**, including all studies**). After the removal of 26 results (25 outliers and 2 influencers), 133 results were analyzed and showed that the antidepressant effect of CBD remained moderate and with low heterogeneity (overall effect (SMD): −0.71; 95% CI = [−0.81; −0.61]; p < 0.0001; heterogeneity: I² = 24.1%, 95% CI = [5.3%; 39.2%]; Q = 173.98, p = 0.0084) (Fig. 3A, **excluding outliers**).

Interestingly, moderate antidepressant effects of CBD were seen across all behavioral paradigms (forced swim test: SMD = −0.74; 95% CI = [−0.86, −0.62]; I² = 18.2%; k = 86; tail suspension test: SMD = −0.73; 95% CI = [−1.00, −0.46]; I² = 34.6%; k = 25; sucrose preference test: SMD = −0.56; 95% CI = [−0.84, −0.28]; I² = 31.1%; k = 22; Fig. 3A; Supplementary Fig. S10).

Subgroup analysis by sex showed moderate effects in males (SMD: −0.75; 95% CI = [−0.86; −0.64]; I² = 23.5%; k = 111) and small effects in females (SMD: −0.47; 95% CI = [−0.74; −0.21]; I² = 17.4%; k = 20). Studies including both sexes did not reach significance (SMD: −0.70; 95% CI = [−4.74; 3.33]; I² = 0.0%; k = 2), likely due to the small number of studies. Overall, effect sizes were more pronounced in males, although the direction of the effect was consistent across sexes (Supplementary Fig. S11A, **including all studies**; Supplementary Fig. S11B, **excluding outliers**).

We found similar moderate effect sizes in rats (SMD: −0.67; 95% CI = [−0.80; −0.54]; I² = 23.3%; k = 78) and mice (SMD: −0.76; 95% CI = [−0.92; −0.59]; I² = 25.3%; k = 55), with no evidence of significant differences between species (Supplementary Fig. S12A, **including all studies;** Supplementary Fig. S12B, **excluding outliers**).

For intraperitoneal administration, all doses showed a significant effect on depressive-like behavior (low: SMD: −0.59; 95% CI = [−0.73; −0.45]; I² = 14.1%; k = 57; moderate: SMD: −0.82; 95% CI = [−1.22; −0.42]; I² = 0.0%; k = 7; high: SMD: −0.83; 95% CI = [−1.04; −0.61]; I² = 33.1%; k = 38) with no significant differences were observed between dose groups (Q = 4.42, p = 0.11) (Supplementary Fig. S13A, **including all studies** and Fig. S13B, **excluding outliers**). For oral administration, high doses (SMD: −0.56; 95% CI = [−0.93; −0.19]; I² = 43.6%; k = 15) showed a significant effect, whereas moderate doses (SMD: −0.44; 95% CI = [−2.18; 1.30]; I² = 61.8%; k = 3) did not reach statistical significance, likely due to the low number of studies. Low doses were not included in this analysis due to the limited number of available studies (k = 2). No differences were observed between dose groups (Q = 0.07, p = 0.79) (Supplementary Fig. S13C, **including all studies** and Fig. S13D, **excluding outliers**).

All disease models assessed showed a robust effect of CBD in reducing depressive-like behavior, with varying effect sizes across models (Fig. 3B, **including all studies**; Supplementary Fig. S14**, excluding outliers**). The largest effects were observed in cerebral ischemia (SMD: −1.07; 95% CI = [−1.92; −0.22]; I² = 0.0%; k = 2) and Parkinson’s disease (SMD: −0.91; 95% CI = [−1.72; −0.09]; I² = 0.0%; k = 2). Moderate anti-depressant effects were found in animals with no underlying pathology (SMD: −0.74; 95% CI = [−0.90; −0.58]; I² = 32.7%; k = 66) and in pain (SMD: −0.78; 95% CI = [−1.23; −0.33]; I² = 0.0%; k = 5), type 1 diabetes (SMD: −0.72; 95% CI = [−1.09; −0.35]; I² = 22.5%; k = 14), model of depression (SMD: −0.67; 95% CI = [−1.01; −0.34]; I² = 23.1%; k = 12), LPS-induced inflammation (SMD: −0.61; 95% CI = [−1.06; −0.15]; I² = 26.3%; k = 9) and stress models (SMD: −0.55; 95% CI = [−0.79; −0.31]; I² = 20.9%; k = 20).

We then proceeded to look at specific behavioral tests subgroup by disease model (Supplementary Fig. S15, **excluding outliers**; Supplementary Fig. S16**, including all studies**). In the forced swim test, the strongest effects were observed in cerebral ischemia (SMD: −1.07; 95% CI = [−1.92; −0.22]; I² = 0.0%; k = 2), Parkinson’s disease (SMD: −0.91; 95% CI = [−1.72; −0.09]; I² = 0.0%; k = 2) and model of depression (SMD: −0.81; 95% CI = [−1.55; −0.07]; I² = 43.3%; k = 5) and in animals with no underlying pathology (SMD: −0.82; 95% CI = [−1.01; −0.63]; I² = 25.5%; k = 41). Moderate antidepressant effects were detected in pain (SMD: −0.74; 95% CI = [−1.39; −0.09]; I² = 0.0%; k = 4), type 1 diabetes (SMD: −0.72; 95% CI = [−1.09; −0.35]; I² = 22.5%; k = 14), and LPS-induced inflammation models (SMD: −0.60; 95% CI = [−1.30; 0.10]; I² = 32.0%; k = 5). Small effect was seen in stress models (SMD: −0.49; 95% CI = [−0.77; −0.22]; I² = 0.0%; k = 12).

In the tail suspension test, CBD produced a moderate reduction of depressive-like behaviors in animals with no underlying pathology (SMD: −0.61; 95% CI = [−0.93; −0.30]; I² = 38.7%; k = 20) but it did not reach significancy in stress models (SMD: −1.16; 95% CI = [−2.40; 0.08]; I² = 0.0%; k = 2). For the sucrose preference test, effects were moderate in model of depression (SMD: −0.57; 95% CI = [−1.04; −0.11]; I² = 10.0%; k = 7) and small in stress (SMD: −0.47; 95% CI = [−1.20; 0.26]; I² = 46.7%; k = 6). No statistically significant effects were seen in “not a model” (SMD: −0.63; 95% CI = [−1.66; 0.41]; I² = 56.1%; k = 5) and LPS-induced inflammation (SMD: −0.43; 95% CI = [−2.22; 1.35]; I² = 39.2%; k = 3).

Asymmetry was detected with Egger’s test, indicating publication bias (intercept = −1.90; 95% CI = [−3.03, −0.77]; t = −3.31; p = 0.0012; Supplementary Fig. S17). After outlier removal and trim-and-fill, 15 studies were added. The effect size changed from −0.71 to −0.61 (95% CI = [−0.72; −0.50]; p < 0.0001), therefore it remained moderate and significant. None of the parameters evaluated as moderators were found to be significant in the meta-regression.

### Memory

**Fig. 4:**
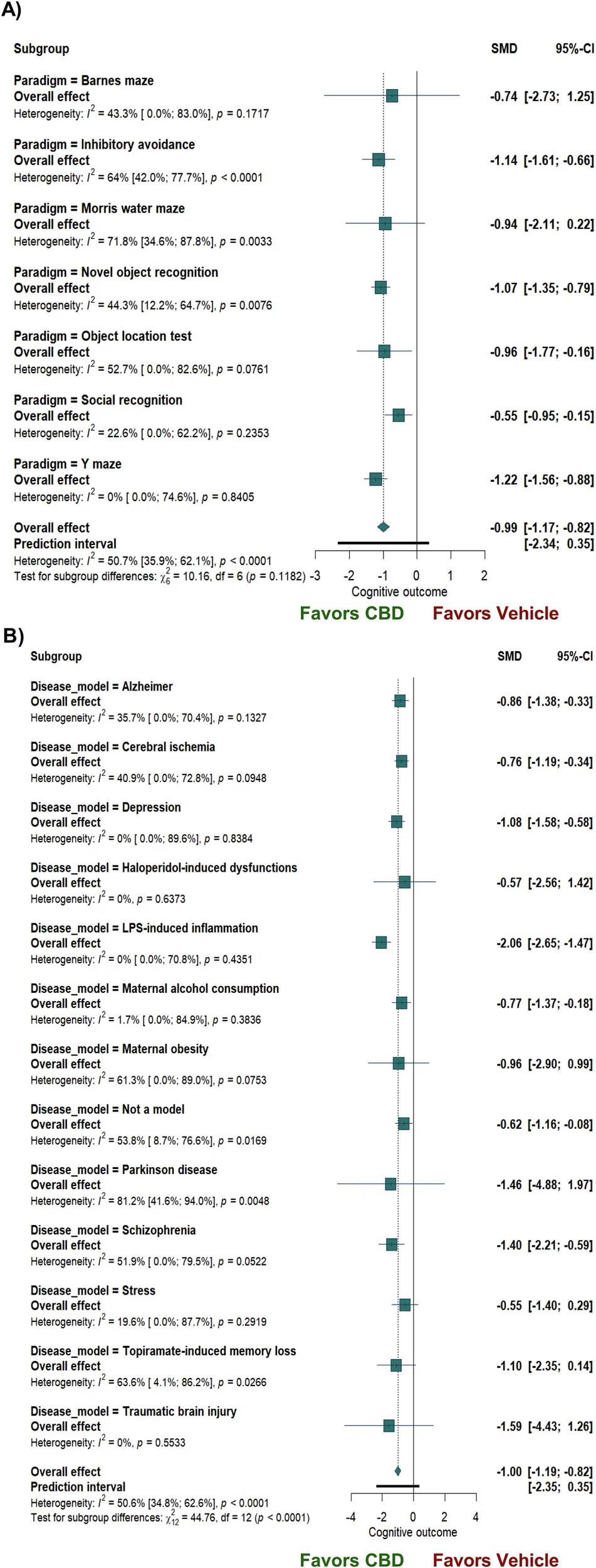
Forest plots of meta-analyses evaluating cognition and memory. **A** Forest plot of the meta-analysis comparing the performance of animals treated with CBD versus vehicle across the evaluated cognitive and memory tests. **B** Forest plot of the meta-analysis comparing the performance of animals treated with CBD versus vehicle subgrouped by disease model independent of cognitive test. Outliers and influential results were excluded prior to analysis. Negative values indicate a beneficial effect of CBD, reflecting better performance relative to vehicle-treated animals. CBD, cannabidiol; CI, confidence interval; DF, degrees of freedom; SMD, standardized mean difference.

We analyzed memory and cognitive domains separately from fear memory due to differences in their interpretation. A total of 102 outcomes from 50 different studies were included in the analysis. CBD produced a large and statistically significant improvement in cognitive performance and memory compared to vehicle-treated groups (overall effect (SMD): −1.20; 95% CI = [−1.59; −0.80]; p < 0.0001; prediction interval = [−5.03 to 2.64]; heterogeneity: I² = 95.3%, 95% CI = [94.7%; 95.8%]; Q = 2132.10, p < 0.0001) (Supplementary Fig. S18, **including all studies**). After removing outliers, 77 results were analyzed, showing that the effect of CBD on cognitive performance remained large and beneficial (overall effect (SMD): −0.99; 95% CI = [−1.17; −0.82]; p < 0.0001; prediction interval = [−2.34 to 0.35]; heterogeneity: I² = 50.7%, 95% CI = [35.9%; 62.1%]; Q = 154.13, p < 0.0001) (Fig. 4A, **excluding outliers**).

The beneficial effects of CBD on cognitive performance with a large and statistically significant effect were observed in several paradigms (Fig. 4A; Supplementary Fig. S18). Specifically, Y maze (SMD: −1.22; 95% CI = [−1.56, −0.88]; I² = 0.0%; k = 6), object location test (SMD: −0.96; 95% CI = [−1.77, −0.16]; I² = 52.7%; k = 5), novel object recognition (SMD: −1.07; 95% CI = [−1.35, −0.79]; I² = 44.3%; k = 27), inhibitory avoidance (SMD: −1.14; 95% CI = [−1.61, −0.66]; I² = 64.0%; k = 20), and social recognition (SMD: −0.55; 95% CI = [−0.95, −0.15]; I² = 22.6%; k = 10), all showed effect sizes favoring CBD. For spatial memory tasks, Barnes maze (SMD: −0.74; 95% CI = [−2.73, 1.25]; I² = 43.3%; k = 3) and Morris water maze (SMD: −0.94; 95% CI = [−2.11, 0.22]; I² = 71.8%; k = 6) did not reach statistical significance. Overall, the results indicate that CBD maintains a beneficial impact on various cognitive domains even after outlier removal.

Subgroup analysis by sex showed significant large effect in males (SMD: −1.07; 95% CI = [−1.27; −0.87]; I² = 53.7%; k = 65) and in studies including both sexes (SMD: −0.84; 95% CI = [−1.09; −0.59]; I² = 0.0%; k = 5). In females, the effect was moderate, but borderline significant (SMD: −0.53; 95% CI = [−1.06; −0.01]; I² = 22.6%; k = 6), which should be interpreted with caution given the small number of studies. Overall, the magnitude of the effect was greater in males, although the direction was consistent across groups (Supplementary Fig. S19A, **including all studies;** Supplementary Fig. S19B, **excluding outliers**).

Subgroup analyses based on species revealed similar large effects of CBD in mice (SMD: −0.92; 95% CI = [−1.16; −0.69]; I² = 51.2%; k = 43) and rats (SMD: −1.08; 95% CI = [−1.36; −0.80]; I² = 50.5%; k = 34) (Supplementary Fig. S20A, **including all studies**; Supplementary Fig. S20B**, excluding outliers**).

For intraperitoneal administration, all doses showed a significant effect on cognitive outcomes (low: SMD: −0.89; 95% CI = [−1.13; −0.65]; I² = 47.0%; k = 37; moderate: SMD: −1.48; 95% CI = [−2.30; −0.66]; I² = 58.0%; k = 7; high; SMD: −0.67; 95% CI = [−1.00; −0.34]; I² = 22.4%; k = 13) with no significant differences between dose groups (Q = 5.03, p = 0.08) (Supplementary Fig. S21A, **including all studies**; Supplementary Fig. S21B**, excluding outliers**). For oral administration, all doses showed a significant effect on cognitive outcomes again (low: SMD: −1.71; 95% CI = [−2.44; −0.98]; I² = 61.6%; k = 9; moderate: SMD: −1.04; 95% CI = [−1.32; −0.75]; I² = 0.0%; k = 3; high: SMD: −0.97; 95% CI = [−1.84; −0.10]; I² = 60.2%; k = 7) with no significant differences were observed between dose groups (Q = 4.44, p = 0.11) (Supplementary Fig. S21C, **including all studies;** Supplementary Fig. S21D, **excluding outliers**).

When two or more results were available for the same experimental model, it was analyzed as a subgroup (Fig. 4B, **excluding outliers;** Supplementary Fig. S22, **including all studies**). CBD significantly improved cognitive performance in several disease models. Large effects were found in Alzheimer’s disease (SMD: −0.86; 95% CI = [−1.38, −0.33]; I² = 35.7%; k = 9), cerebral ischemia (SMD: −0.76; 95% CI = [−1.19, −0.34]; I² = 40.9%; k = 9), and model of depression (SMD: −1.08; 95% CI = [−1.58, −0.58]; I² = 0.0%; k = 3). A strong effect was also found in LPS-induced inflammation (SMD: −2.06; 95% CI = [−2.65, −1.47]; I² = 0.0%; k = 7) and in schizophrenia (SMD: −1.40; 95% CI = [−2.21, −0.59]; I² = 51.9%; k = 7). Moderate and statistically significant improvement was observed in maternal alcohol exposure (SMD: −0.77; 95% CI = [−1.37, −0.18]; I² = 1.7%; k = 4) and in the subgroup not classified as a model (SMD: −0.62; 95% CI = [−1.16, −0.08]; I² = 53.8%; k = 11). No significant effects were detected in haloperidol-induced dysfunctions (SMD: −0.57; 95% CI = [−2.56, 1.42]; I² = 0.0%; k = 2), maternal obesity (SMD: −0.96; 95% CI = [−2.90, 0.99]; I² = 61.3%; k = 3), Parkinson’s disease (SMD: −1.46; 95% CI = [−4.88, 1.97]; I² = 81.2%; k = 3), stress (SMD: −0.55; 95% CI = [−1.39, 0.29]; I² = 19.6%; k = 4), topiramate−induced memory loss (SMD: −1.10; 95% CI = [−2.35, 0.14]; I² = 63.6%; k = 5), and traumatic brain injury (SMD: −1.59; 95% CI = [−4.43, 1.26]; I² = 0.0%; k = 2).

When two or more results were available for the same experimental model and the same model, it was analyzed as a subgroup (Supplementary Fig. S23, **excluding outliers**; Supplementary Fig. S24, **including all studies**). The effects of CBD on cognitive performance varied according to the task and disease model. In the Barnes maze, no significant effect was found in animals not subjected to a disease model (SMD: −0.33; 95% CI = [−0.91, 0.26]; I² = 0.0%; k = 2) and the other models in this test did not reach the minimum number of studies required for inclusion. In the inhibitory avoidance task, large and significant improvement was detected in LPS-induced inflammation (SMD: −2.47; 95% CI = [−3.62, −1.31]; I² = 0.0%; k = 3) and schizophrenia models (SMD: −1.46; 95% CI = [−2.79, −0.13]; I² = 67.5%; k = 5). No significant effects were observed in topiramate-induced memory loss (SMD: −1.10; 95% CI = [−2.35, 0.14]; I² = 63.6%; k = 5) or in studies not classified as a model (SMD: −0.29; 95% CI = [−0.77, 0.18]; I² = 0.0%; k = 4).

In the Morris water maze, only results from cerebral ischemia were included and showed no significant change (SMD: −0.33; 95% CI = [−2.22, 1.57]; I² = 0.0%; k = 2). For the novel object recognition test, significant improvements were observed in model of depression (SMD: −1.08; 95% CI = [−1.58, −0.58]; I² = 0.0%; k = 3) and cerebral ischemia (SMD: −0.66; 95% CI = [−0.66, −0.66]; I² = 0.0%; k = 2), but not for other models included in this test (Alzheimer’s disease: SMD = −1.19; 95% CI = [−3.39, 1.00]; I² = 58.9%; k = 3; maternal alcohol exposure: SMD = −0.64; 95% CI = [−5.20, 3.92]; I² = 54.7%; k = 2;, LPS-induced inflammation: SMD = −1.95; 95% CI = [−11.76, 7.86]; I² = 65.5%; k = 2; schizophrenia: SMD = −1.32; 95% CI = [−3.53, 0.89]; I² = 0.0%; k = 2; Parkinson’s disease: SMD = −0.77; 95% CI = [−7.68, 6.15]; I² = 62.8%; k = 2; haloperidol-induced dysfunctions: SMD = −0.57; 95% CI = [−2.56, 1.42]; I² = 0.0%; k = 2); and in studies not classified as a model: SMD = −1.41; 95% CI = [−4.47, 1.66]; I² = 81.6%; k = 3).

For the object location test, animals exposed to cerebral ischemia did not show significant effects (SMD: −0.98; 95% CI = [−3.30, 1.35]; I² = 76.0%; k = 3). In the social recognition task, the effects were not significant in Alzheimer’s disease (SMD: −0.51; 95% CI = [−1.30, 0.28]; I² = 3.5%; k = 4), maternal obesity (SMD: −0.96; 95% CI = [−2.90, 0.99]; I² = 61.3%; k = 3), or stress (SMD: −0.09; 95% CI = [−0.84, 0.65]; I² = 0.0%; k = 2).). For the Y maze, CBD markedly improved performance in LPS-induced inflammation (SMD: −1.70; 95% CI = [−2.63, −0.77]; I² = 0.0%; k = 2), whereas in cerebral ischemia models the effect was not significant (SMD: −1.13; 95% CI = [−2.37, 0.11]; I² = 0.0%; k = 2).

Asymmetry was detected with Egger’s test, indicating publication bias (intercept = −4.21; 95% CI = [−5.52, −2.89]; t = −6.27; p < 0.0001; Supplementary Fig. S25). After removing outliers and applying the trim-and-fill procedure, 16 studies were added, and the overall effect size decreased from −0.99 to −0.71 (SMD: −0.71; 95% CI = [−0.93, −0.50]; p < 0.0001), showing that even when accounting for publication bias, the effects remain moderate and robust. None of the parameters evaluated as moderators were found to be significant in the meta-regression.

### Fear memory

**Fig. 5:**
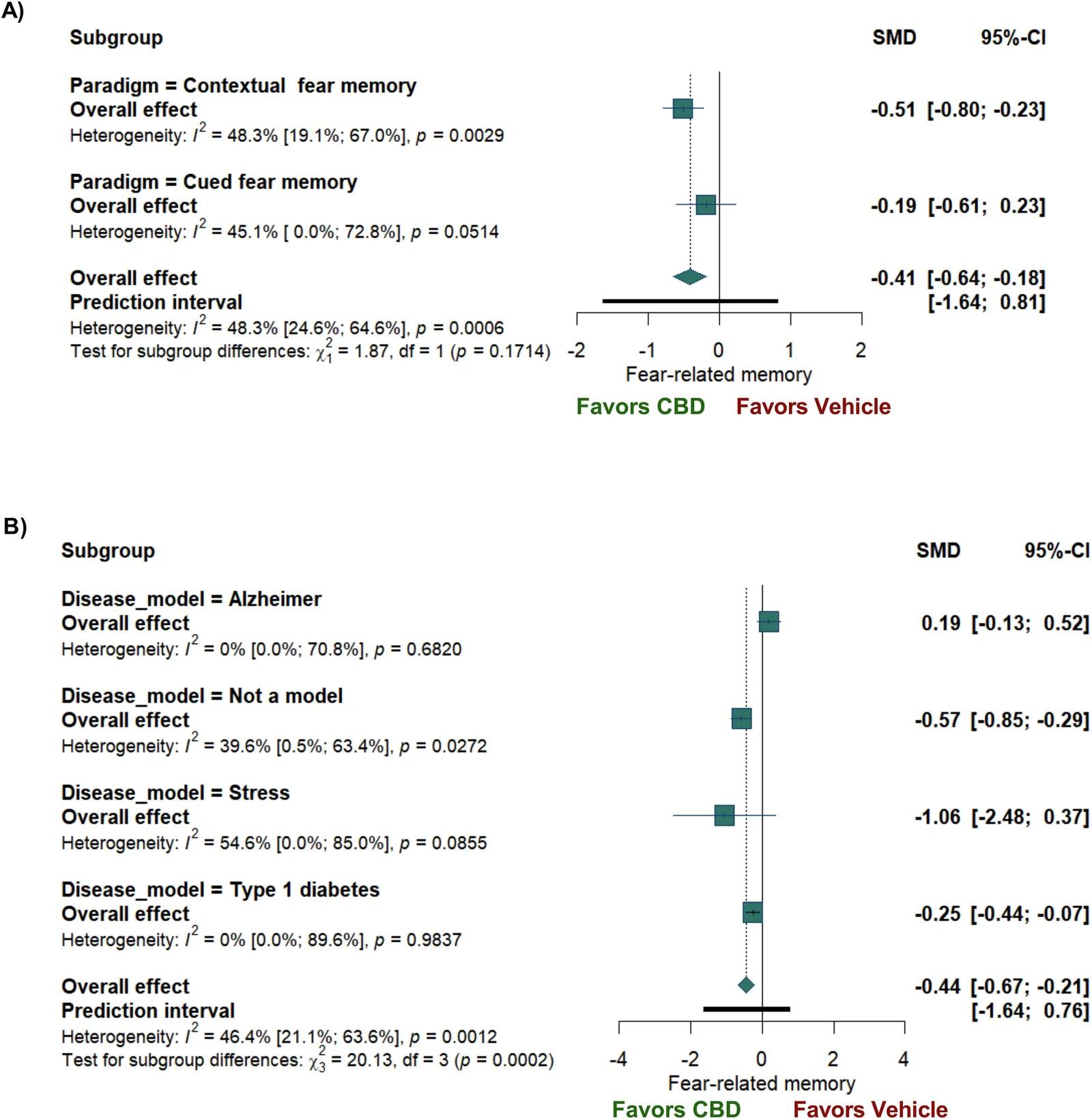
Forest plots of meta-analyses evaluating fear-related behaviors. **A** Forest plot of the meta-analysis comparing the performance of animals treated with CBD versus vehicle in fear-memory related paradigms. **B** Forest plot of the meta-analysis comparing the performance of animals treated with CBD versus vehicle subgrouped by model independent of the fear-related test. Outliers and influential results were excluded prior to analysis. Negative values indicate a beneficial effect of CBD, reflecting a reduced aversive response when animals are exposed to the aversive stimulus. CBD, cannabidiol; CI, confidence interval; DF, degrees of freedom; SMD, standardized mean difference.

We decided to analyze fear-related memories (cued or contextual) separately, as most studies interpret CBD’s beneficial effect in these paradigms as a reduction in fear responses (freezing). Thus, in this context, CBD’s positive impact is reflected in the attenuation of aversive memory recall. A total of 46 results from 20 different studies were included. CBD showed a beneficial effect in paradigms related to fear memory, promoting reduced consolidation of aversive memories, which is interpreted as an increased ability to cope with emotions in CBD-treated animals compared to vehicle-treated groups (overall effect (SMD): −0.57; 95% CI = [−0.96; −0.18]; p = 0.0049; prediction interval = [−3.15 to 2.00]; heterogeneity: I² = 74.4%, 95% CI = [65.9%; 80.8%]; Q = 175.54, p < 0.0001) (Supplementary Fig. S26, **including all studies**). After removing outliers, 38 results were analyzed, showing a small and statistically significant beneficial effect of CBD on fear memory (overall effect (SMD): −0.41; 95% CI = [−0.64, −0.18]; t = −3.63; p = 0.0009; prediction interval = [−1.64 to 0.81]; heterogeneity: I² = 48.3%, 95% CI = [24.6%; 64.6%]; Q = 71.60, p = 0.0006) (Fig. 5A, **excluding outliers**).

The beneficial effect of CBD in contextual fear memory is moderate (SMD: −0.51; 95% CI = [−0.80, −0.23]; I² = 48.3%; k = 27), while the effect in cued fear memory is small and not significant (SMD: −0.19; 95% CI = [−0.61, 0.23]; I² = 45.1%; k = 11) (Fig. 5A; Supplementary Fig. S26).

Subgroup analysis by sex showed a significant small effect in males (SMD: −0.35; 95% CI = [−0.58; −0.12]; I² = 40.6%; k = 34). In females, the effect was not statistically significant (SMD: −0.94; 95% CI = [−2.36; 0.49]; I² = 74.2%; k = 4) and should be interpreted with caution given the small number of studies and high heterogeneity (Supplementary Fig. S27A, **including all studies**; Supplementary Fig. S27B, **excluding outliers**). Subgroup analyses based on species revealed a small effect in mice (SMD: −0.33; 95% CI = [−0.64; −0.02]; I² = 52.9%; k = 22) and a moderate effect in rats (SMD: −0.53; 95% CI = [−0.91; −0.16]; I² = 40.8%; k = 16). Although the effect size appeared larger in rats, there was no evidence of significant differences between species (Supplementary Fig. S28A, **including all studies;** Supplementary Fig. S28B, **excluding outliers**).

For fear-related memory, only intraperitoneal administration was analyzed due to the limited number of studies employing other regimens. Low doses (SMD: −0.86; 95% CI = [−1.37; −0.36]; I² = 46.4%; k = 10) showed a significant effect. In contrast, moderate (SMD: 0.35; 95% CI = [−0.06; 0.76]; I² = 0.0%; k = 2) and high doses (SMD: −0.04; 95% CI = [−0.43; 0.35]; I² = 25.4%; k = 10) did not show significant effects. A significant difference between dose groups was observed (Q = 33.20, p < 0.0001), suggesting a dose-dependent effect, with beneficial effects restricted to low doses (Supplementary Fig. S29A, **including all studies;** Supplementary Fig. S29B, **excluding outliers**). However, this finding should be interpreted with caution, as it may be influenced by the unequal number of studies across subgroups, particularly the low number of studies in the moderate-dose group.

When two or more results were available for the same experimental model, they were analyzed as a subgroup (Fig. 5B, **including all studies;** Supplementary Fig. S30, **excluding outliers**). No significant effect was observed in Alzheimer’s disease (SMD: 0.19; 95% CI = [−0.13, 0.52]; I² = 0.0%; k = 7) or in stress (SMD: −1.06; 95% CI = [−2.48, 0.37]; I² = 54.6%; k = 4). A small but statistically significant effect was found in Type 1 diabetes (SMD: −0.25; 95% CI = [−0.44, −0.07]; I² = 0.0%; k = 3). Interestingly, the subgroup not classified as a model showed a moderate effect (SMD: −0.57; 95% CI = [−0.85, −0.29]; I² = 39.6%; k = 23). These results indicate that, in the context of animals without underlying disease and with type 1 diabetes, CBD treatment improves the ability to cope with fear memories and results in a reduced freezing response.

When two or more results were available for the same experimental model, it was analyzed as a subgroup (Supplementary Fig. S31, **excluding outliers**; Supplementary Fig. S32, **including all studies**). The effects of CBD on fear memory varied according to the memory type and disease model. In contextual fear memory, moderate and significant improvement was found in animals not subjected to a disease model (SMD: −0.60; 95% CI = [−0.96, −0.24]; I² = 49.9%; k = 18) and small effect in type 1 diabetes (SMD: −0.25; 95% CI = [−0.44, −0.07]; I² = 0.0%; k = 3). No significant effects were detected in stress (SMD: −0.94; 95% CI = [−3.58, 1.70]; I² = 66.2%; k = 3) or Alzheimer’s disease (SMD: 0.12; 95% CI = [−1.07, 1.31]; I² = 11.3%; k = 3). In cued fear memory, a moderate effect was observed in animals not classified as a model (SMD: −0.50; 95% CI = [−0.94, −0.06]; I² = 0.0%; k = 5), whereas no significant effect was found in Alzheimer’s disease (SMD: 0.24; 95% CI = [−0.25, 0.73]; I² = 0.0%; k = 4). Other models in these tests did not reach the minimum number of studies required for inclusion.

Egger’s test indicated significant funnel plot asymmetry (intercept = −3.81; 95% CI = [−6.04, −1.58]; t = −3.35; p = 0.0019; Supplementary Fig. S33). Trim-and-fill procedure added 3 studies, and the effect size changed but was still small (from −0.41 to −0.32; 95% CI = [−0.58; −0.06]; p = 0.0174). Number of administrations (R² = 67.98%; p = 0.0027; estimate = 0.2390; t = 1.2512) and disease model (R² = 72.66%; p = 0.0077; estimate = 0.1928; t = 0.9610) were identified as moderators, indicating that single administration and the type of disease model influenced CBD’s effect on fear-memory.

## Discussion

In recent years, interest in the therapeutic potential of cannabidiol (CBD) has grown considerably, particularly regarding its effects on the central nervous system. CBD has been shown to exert neuroprotective, anti-inflammatory, and antioxidant actions, without eliciting psychotropic effects. A key feature of CBD is its broad pharmacological activity, modulating the endocannabinoid system - including anandamide, 2-AG, and CB1/CB2 receptors - as well as other molecular targets such as 5-HT1A, TRPV1, PPARγ, and adenosine receptors. These cellular and molecular mechanisms of action have been extensively reviewed [10]. Importantly, many of these targets are also widely expressed in the gastrointestinal tract, and together with reported modulatory effects of CBD on gut microbiota composition and intestinal permeability, this suggests that some of its central effects may be mediated, at least in part, by the gut–brain axis, as discussed in previous reviews [23]. This wide-ranging activity may underlie the cognitive and behavioral effects reported for CBD, including anxiolytic and antidepressant actions [24–26]. However, inconsistences in the literature remain, and it is still unclear whether CBD consistently improves cognition or behavior. Two main factors motivated the present meta-analysis. First, preclinical studies assess cognitive and behavioral domains using a diverse array of tests, whose specificities are not always accounted for when interpreting CBD’s effects. Second, outcomes may vary depending on whether animals are healthy or exposed to experimental disease models. Our findings indicate that CBD’s effects are not uniform, varying across behavioral domains, test types, and experimental models, highlighting the importance of careful and context-specific interpretation.

We also identified sex-specific differences, with CBD showing stronger effects in males, particularly in reducing depressive-like behavior, improving cognitive performance, and enhancing the ability to cope with fear-related memories. However, these findings should be interpreted cautiously, since the smaller effects observed in females may reflect reduced statistical power and variability across studies rather than true biological differences. Evidence from the literature has also demonstrated sex-dependent responses to CBD [27]. Interestingly, females appear to exhibit higher plasma and concentrations of CBD, suggesting greater bioavailability [28]. Despite this, behavioral effects can be more pronounced in males, indicating that differences in biological sensitivity to CBD may underlie these findings [27]. One possible explanation involves hormonal modulation of the endocannabinoid system, as estrogen has been shown to regulate FAAH expression [29, 30], potentially influencing endocannabinoid tone and CBD responsiveness in females. Additionally, sex-specific differences in other molecular targets of CBD may contribute to variability in behavioral outcomes, such as differential responses to stress hormones [31] and sex-dependent functioning of receptors including 5-HT1A [32]. Therefore, the differences observed in this meta-analysis may be supported by these biological mechanisms. However, it is essential that more studies are conducted in females to increase the robustness of the analyses and to confirm these findings.

We will next examine CBD’s effects across individual behavioral domains, emphasizing differences related to test paradigms and experimental models.

### Anxiety-like behaviors

Our results indicate that CBD consistently reduces anxiety-like behaviors across the tests, although the magnitude of this effect varies by paradigm, likely reflecting the distinct dimensions of anxiety-like behavior captured by each test. The effect was small in the open field and light-dark box tests, but moderate in the elevated plus maze and novelty-suppressed feeding and remained robust after excluding outliers or influential studies. When examining each behavioral test by disease model, the effects of CBD appeared more heterogeneous, likely reflecting the limited number of studies within each model and the variability of results within them.

The anxiolytic effects of CBD are mediated by multiple mechanisms, primarily involving serotonergic and endocannabinoid systems, as well as modulation of neuroinflammation and neuroplasticity. Among this, CBD’s interaction with 5-HT1A receptors appear particularly important, although CB1 and CB2 receptors also contribute depending on the model and brain region studied. For instance, CBD exerts anxiolytic effects in models of epilepsy, neuropathic pain, and acute restraint stress, effects abolished by pre-administration of the 5-HT1A receptor antagonist WAY100635 [24, 33, 34]. Direct binding of CBD to 5-HT1A receptors has been confirmed in vivo using PET imaging and is associated with reduced anxiety-like behavior in an osteoarthritis model [25]. Region-specific studies further support this involvement of 5-HT1A receptors. In the dorsolateral periaqueductal gray, CBD’s anxiolytic effect depended on 5-HT1A signaling and was not influenced by the CB1 agonist AM251 [35]. Similarly, administration of CBD into the prelimbic medial prefrontal cortex produced anxiolytic responses in stressed animals, which were blocked by the 5-HT1A antagonist WAY100635 [36]. Collectively, these findings indicate that CBD acts at corticolimbic circuitry, with serotonergic signaling via 5-HT1A receptors playing a central role.

Endocannabinoid signaling also contributes to the anxiolytic effects of CBD, though its involvement is more variable. CB1 receptor knockout or pharmacological blockade prevents some anxiolytic responses to CBD, whereas CB2 and GPR55 do not appear necessary in certain contexts [37]. In chronic stress models, CBD restored CB1 and CB2 expression in the amygdala, which was associated with anxiolytic effects [38]. Similarly, in type 1 diabetes model, blockade of 5-HT1A or CB1 receptors prevented CBD’s anxiolytic effect, while CB2 blockade had no effect [39]. In contrast, in HIV-1 Tat transgenic mice, acute anxiolytic effects occurred independently of changes in endocannabinoid levels or CB1/CB2 expression [40]. Notably, although 5-HT1A blockade typically abolishes CBD’s anxiolytic effects, in a chronic stress model study, anxiolysis persisted despite 5-HT1A inhibition, but was abolished by CB1 or CB2 antagonists [41], indicating context-dependent contributions of the endocannabinoid system.

CBD’s anti-inflammatory actions and modulation of neurotrophic pathways also appear to contribute to its anxiolytic effects. CBD reduced neuroinflammation in the prefrontal cortex and hippocampus while improving anxiety- and depressive-like behaviors in chemotherapy-induced dysfunction [42]. Similarly, offspring of obese mothers treated with CBD showed reduced neuroinflammation in the prefrontal cortex and hippocampus, associated with reduced anxiety-like behavior, along with modulation of the endocannabinoid system and normalization of dopamine and oxytocin receptor levels in the prefrontal cortex [43, 44]. In a type 1 diabetes model, CBD also restored norepinephrine and serotonin levels in the hippocampus and prefrontal cortex, which was associated with anxiolytic effects [45].

Neuroplasticity also appears critical in this scenario: CBD restored serotonergic and glutamatergic signaling, increased BDNF and PPARδ expression, and produced anxiolytic, antidepressant, and cognitive improvements in chronically stressed animals [46]. Similar effects were seen in other models: an anxiolytic effect related to increased BDNF and reduced neuroinflammation in the prefrontal cortex and hippocampus was reported in a cerebral malaria model [47]. The neuroprotective and anxiolytic-like effects of CBD in cerebral ischemia models included hippocampal neurogenesis, dendritic remodeling, glial modulation, and increased BDNF [48, 49], with anxiolytic effects attenuated by CB1, CB2, 5-HT1A, and PPAR-γ antagonists [50].

In addition to these anti-inflammatory and neurotrophic actions, CBD modulates stress-response systems. In chronic stress model, CBD reversed alterations in the expression of key receptors, including corticotropin-releasing factor (CRF), in the paraventricular nucleus and glucocorticoid receptors (GR) in the hippocampus, contributing to anxiolytic effects [38].

In summary, while 5-HT1A receptor antagonism consistently demonstrates involvement of this receptor in CBD’s anxiolytic effects, complementary mechanisms - including endocannabinoid signaling, anti-inflammatory actions, neurotrophic modulation, and stress-system regulation - also contribute. Importantly, studies administering CBD during adolescence reported no harmful effects on neurodevelopment, and in some cases, observed anxiolytic and cognitive benefits [51].

### Depressive-like behaviors

Interestingly, in all paradigms assessing depressive-like outcomes included in this meta-analysis - forced swim test, tail suspension test, and sucrose preference - CBD consistently maintained a moderate effect size in reducing depressive-like behaviors, even after the removal of outliers and influential studies. This pattern suggests that, unlike the more variable results observed in paradigms to test anxiety-like behaviors, CBD may elicit a more homogeneous response across tests of depressive-like behavior. When examining each paradigm separately, the effects of CBD appeared more variable across disease models, likely due both to the limited number of studies reporting each outcome and to the differences observed in effect sizes across studies. Overall, these findings indicate that CBD exerts a robust antidepressant effect across the different behavioral paradigms.

Some reports indicate that genetic depletion of CB1, CB2, or GPR55 does not block CBD’s antidepressant-like effect [37], and in other studies, CBD produces antidepressant-like behavior without altering CB1 or CB2 receptor expression or hippocampal cell proliferation markers [52]. On the other hand, in a neuropathic pain model, the antidepressant effect of CBD appears to involve both CB1 and 5-HT1A receptors, as administration of antagonists abolishes the effect in both cases [53]. As seen in anxiety-like behaviors, 5-HT1A receptor activation appears central for antidepressant-like effects in many studies. It has been shown that CBD increases serotonin and glutamate levels in the prefrontal cortex, and the antidepressant-like effect was lost after 5-HT1A antagonism [54]. Other studies have shown that blocking serotonin synthesis abolishes CBD’s antidepressant effects, while in vivo PET imaging has demonstrated direct binding of CBD to 5-HT1A receptors, an interaction associated with antidepressant effects in an osteoarthritis model [25, 55]. In chronic stress models, CBD’s antidepressant effects are mediated by 5-HT1A receptors in the prefrontal cortex, as prior administration of the antagonist WAY100635 abolishes these effects, and CBD also modulated microRNAs miR-16 and miR-135, which regulate serotonergic neurotransmission and contribute to its antidepressant-like action [56]. Intracerebral administration of CBD into the ventromedial prefrontal cortex (including prelimbic and infralimbic regions) reduces immobility in the forced swim test, and this effect is blocked by the 5-HT1A antagonism, underscoring the importance of region-specific serotonergic mechanisms [57]. Similarly, 5-HT1A antagonism abolishes CBD-antidepressant-like effects without altering BDNF [58].

Anti-inflammatory effects also appear relevant to these antidepressant outcomes of CBD. Increases in serotonin and dopamine require high doses of CBD, whereas the anti-inflammatory effect occurs across a broader range of doses capable of inhibiting NF-κB signaling [59]. In an LPS-induced inflammation model, CBD diminished cortical NF-κB activation, reduced IL-6 in plasma and brain, and normalized kynurenine-to-tryptophan (KYN/TRP) and kynurenine-to-serotonin (KYN/5-HT) ratios in hippocampus and cortex, linking anti-inflammatory action to behavioral improvement [60]. In another LPS model, CBD prevented behavioral changes, reduced IL-6 in hypothalamus and hippocampus, and normalized BDNF and nerve growth factor (NGF) levels in hippocampus, suggesting that CBD’s modulation of both inflammatory and neurotrophic responses [61]. These data illustrate that CBD does not uniformly increase BDNF; rather, its effect depends on the baseline or model-induced dysregulation restoring deficient levels or reducing pathological overexpression. Activation of BDNF-TrkB-mTOR pathway in the hippocampus and amygdala was implicated as necessary for CBD’s antidepressant effects [62, 63]. In chronic stress models, CBD reduced depressive-like behaviors associated with increased BDNF in prefrontal cortex (without changing BDNF levels in the hippocampus), decreased IBA-1 transcripts in prefrontal cortex, and increased synaptophysin in both prefrontal cortex and hippocampus [64].

All these CBD’s antidepressant-like effects appear to involve multiple central and peripheral mechanisms converging on synaptic plasticity, epigenetic regulation, and intracellular signaling. In the Flinders Sensitive Line (FSL) model, CBD improved depressive-like behaviors and modulated prefrontal cortex markers related to synaptic function, such as mGluR5, ERK1/2, and synaptophysin, without altering endocannabinoid levels [65]. Epigenetic modulation has also been implicated: CBD restores stress-induced reductions in DNA methyltransferase (DNMT) activity and prevents global hypomethylation in the prefrontal cortex, while reducing pathologically increased methylation in the hippocampus - indicating region-specific effects [66]. Furthermore, CBD modulates the FoxO signaling pathway, rescuing depressive-like symptoms and preventing overactivation and astrocytic differentiation of radial neural stem cells under chronic stress [67]. In a neuropathic pain model, CBD ameliorated depressive-like behaviors and normalized spinal expression of PTGS2, GPR55, SOD1, NQO1, and CYP1A2, enhancing neuronal survival [68]. Collectively, these findings suggest that CBD’s antidepressant effects result from coordinated actions across molecular pathways that support neuronal resilience and adaptive plasticity in both the central nervous systems.

In addition to stress-based and neuropathic models, studies using metabolic paradigms provide further evidence for CBD’s antidepressant potential. In a type 1 diabetes model, CBD produced antidepressant-like effects when administered intraperitoneally at 30 mg/kg, whereas lower doses were ineffective, likely due to insufficient increases in serotonin and norepinephrine levels in the prefrontal cortex and hippocampus [45]. These effects were dependent on CB1, CB2, and 5-HT1A receptors, as pharmacological blockade of any of these receptors abolished the antidepressant response [39]. Notably, CBD’s antidepressant effects in diabetic animals were not observed after acute administration but emerged following sub-chronic treatment (three injections) at 30 mg/kg, with both lower and higher doses failing to produce the effect [69]. These findings suggest that in the context of metabolic dysregulation associated with diabetes, CBD requires an optimal dose range and repeated administration to effectively modulate monoaminergic signaling and engage relevant receptor pathways for antidepressant outcomes.

Taken together, these preclinical data support antidepressant-like effects of CBD mediated by a convergence of serotonergic mechanisms (notably 5-HT1A), anti-inflammatory and neuroplastic changes, epigenetic regulation, and model-dependent receptor involvement.

### Cognition

Preclinical studies on cognitive outcomes demonstrate considerable variability in CBD’s effects across different behavioral paradigms. Overall, effects on spatial memory were mixed: negligible in the Barnes maze and Morris water maze, but large in the object location test and Y-maze. Large effects were also observed in inhibitory avoidance and novel object recognition tasks, while social memory showed moderate effects. All reported outcomes indicated improved memory in CBD-treated animals, although heterogeneity across studies remained substantial. This variability likely reflects differences in the neurobiological substrates engaged by each test, as well as involvement of distinct brain regions. This may have translational implications, as spatial memory tasks are generally associated with hippocampus-dependent navigation in humans, while recognition-based tasks may better reflect episodic-like memory processes relevant to cognitive impairment in neuropsychiatric and neurodegenerative conditions [70]. However, future clinical studies will be necessary to determine whether these domain-specific effects translate to humans. A particularly relevant observation concerns fear-related memory: while cued fear memory was generally unaffected, CBD moderately reduced the fear response in the contextual fear paradigm, suggesting a decreased consolidation of aversive experiences and potentially improved coping with negative stimuli.

CBD enhances cognitive performance across a variety of memory tasks, with the 5-HT1A receptor playing a central role. In a hepatic encephalopathy model, CBD reduced TNF-α levels and increased BDNF in the hippocampus. Otherwise, BDNF increase was prevented by 5-HT1A blockade even in the absence of changes in receptor expression [71]. These findings suggest that serotonergic modulation and downstream neurotrophic pathways contribute to the pro-cognitive effects of CBD, providing a potential mechanism underlying improvements seen in both spatial, social, recognition, and inhibitory memory tasks.

Recognition memory outcomes, however, are mixed. In HIV-1 Tat transgenic mice, object recognition deficits were not ameliorated by CBD, likely due technical or model-related limitations - animals did not appear to discriminate novel versus familiar objects [40]. In a pentylenetetrazole-induced epilepsy model, CBD worsened object recognition despite anticonvulsant benefits [72]. These discrepancies highlight the influence of model-specific factors and task parameters on cognitive outcomes.

Mechanistically, CBD’s cognitive benefits are linked to hippocampal neurogenesis and synaptic/plasticity markers. Neurogenesis was necessary for CBD-mediated cognitive improvement, as blockade with temozolomide abolished benefits [73]. CBD restored serotonergic and glutamatergic signaling, BDNF expression, and other plasticity markers in chronic stress models [46], reversed topiramate-induced deficit [74], and improved cognitive performance in cerebral malaria [47]. CBD also decreased GFAP reactivity in ischemia models and prevented hippocampal neuron death, suggesting that glial modulation contributes to neuroprotection and preserved cognitive function [75].

CBD’s anti-inflammatory and antioxidant actions correlate with cognitive benefit in multiple pathological models, including maternal alcohol exposure [76], LPS-induced inflammation [77], schizophrenia and hypoxia-ischemia models [78, 79], and sepsis [80]. Mitochondrial modulation also contributes, as evidenced by increased levels of the mitochondrial fusion protein Mfn2 in LPS and experimental autoimmune encephalomyelitis (EAE) models [81]. Conversely, a study reported potential memory deficits after CBD via hippocampal mitochondrial dysfunction [82].

In neurodegenerative and injury models, CBD demonstrated variable but generally beneficial cognitive effects. In Alzheimer’s disease models, CBD improved social and spatial memory [26, 83], reversed object recognition deficits [84], modulated inhibitory glycine receptor function [85], reduced Aβ40 [83, 86], and decreased cortical neuroinflammation [87]. In neonatal hypoxia-ischemia [88], traumatic brain injury [89], Parkinson’s disease [90], and Gulf War Illness [91] models, CBD improved cognition and memory, reduced neuroinflammation, oxidative stress, and tau/β-amyloid pathology, and restored mitochondrial and metabolic functions [88–91]. Cognitive benefits were also linked to PPARγ and survival kinase pathways, Akt/GSK3β and Bcl-2 signaling, neurogenesis, dendritic spine density, and improved cerebral glucose metabolism [41, 92–95].

CBD modulation of fear memory has been documented across multiple models, with translational implications for disorders such as post-traumatic stress disorder (PTSD). For instance, CBD reduced fear memory consolidation in contextual fear conditioning paradigms in both female [96] and male models [38, 97]. In type 1 diabetes model, prolonged CBD dosing reduced persistence of contextual fear memory, consistent with anxiolytic effects observed in the elevated plus maze [98]. Direct administration of CBD into the bed nucleus of the stria terminalis confirmed 5-HT1A-dependent reductions in freezing [99], while CB1 receptor signaling was critical for fear extinction processes [100]. Collectively, these findings suggest that CBD may modulate the persistence and consolidation of aversive memories, which may be relevant in conditions characterized by pathological alterations in fear and anxiety-related memory processes. However, the interpretation of these effects is complex, as changes in fear memory may reflect either enhanced aversive memory strength or, in other models, impairments in memory processing.

In this context, the effects of CBD on fear-related behaviors may not be unidirectional, but rather reflect a normalization of altered neurobiological states, as illustrated by its ability to restore cued fear memory via 5-HT1A-mediated mechanisms in an epilepsy model [24].

Notably, not all studies reported beneficial cognitive effects of CBD. Acute CBD administration in the prefrontal cortex impaired cognitive flexibility and spatial working memory in healthy animals [101], and certain epilepsy models [72] or models in which CBD impaired mitochondrial function [82], it also worsened recognition memory [72, 82], emphasizing context dependence and the importance of dosing and timing for translational applications. Overall, preclinical evidence indicates that CBD exerts robust cognitive-protective effects in multiple pathological contexts - including neuroinflammation, neurodegeneration, trauma, metabolic, and toxic insults - through combined anti-inflammatory, antioxidant, neurogenic, neurotrophic, mitochondrial, and survival-signaling mechanisms, while highlighting potential cognitive impairments under specific acute or physiological conditions.

## Final considerations

The studies included in this work were generally of moderate to high quality, as assessed using SYRCLE’s RoB and CAMARADES tools, and none were classified as low quality. This supports confidence in the reliability of the data, though the most common risks of bias involved randomization, blinding, and lack of appropriate sample size calculations. Future studies should address these factors to reduce bias. Additionally, trim-and-fill analyses indicated minimal impact of potential publication bias on overall conclusions. In conclusion, CBD demonstrates robust protective effects on cognition, with consistent anxiolytic and antidepressant outcomes. However, these effects are not uniform and depend on the behavioral test used, cognitive or emotional domain, and the presence of underlying pathology. Future preclinical studies should combine complementary behavioral tests to fully capture CBD’s spectrum of effects. Importantly, these findings are derived from preclinical models and should not be directly extrapolated to clinical settings. Nevertheless, they provide a rational framework to support further mechanistic studies and the design of future clinical trials aimed at evaluating the translational potential of CBD.

Finally, we highlight the limited availability of studies in specific experimental paradigms, as well as the underrepresentation of female animals and oral administration routes. These factors likely contribute to heterogeneity across studies and emphasize the need for more standardized and diverse experimental designs to enable more robust and informative future meta-analyses.

## Supplementary information

Supplementary material

## Data Availability

Data will be made available upon reasonable request.
