# Supplementary material for: Effects of cannabidiol on anxiety- and depressive-like behaviors and cognition: a systematic review and meta-analysis of preclinical studies
Source: Mol Psychiatry. 2026 Jun 30;31(11):6676–92. doi: 10.1038/s41380-026-03733-x (PMC13668667; doi:10.1038/s41380-026-03733-x)
Supplement: Supplementary file 1 [file 41380_2026_3733_MOESM1_ESM.docx]

Title: Effects of cannabidiol on anxiety- and depressive-like behaviors and cognition: a systematic review and meta-analysis of preclinical studies

Jeferson Jantsch, PhD^1^, Fernanda Wickert^1^, Gabriel de Farias Fraga^1^, Luciano Stürmer de Fraga, PhD^2^, Luz Elena Durán-Carabali, PhD^3^, Renata Padilha Guedes, PhD^1,4^

Affiliations:

1 Graduate Program in Biosciences, Federal University of Health Sciences of Porto Alegre (UFCSPA), Porto Alegre, Rio Grande do Sul, Brazil

2 Graduate Program in Physiology, Institute of Basic Health Sciences (ICBS), Federal University of Rio Grande do Sul (UFRGS), Porto Alegre, Rio Grande do Sul, Brazil

3 Department of Physiological Sciences, Federal University of Santa Catarina (UFSC), Florianópolis, Santa Catarina, Brazil

4 Graduate Program in Health Sciences, Federal University of Health Sciences of Porto Alegre (UFCSPA), Porto Alegre, Brazil

Corresponding author: Renata Padilha Guedes

e-mail: [renata.guedes@ufcspa.edu.br](mailto:renata.guedes@ufcspa.edu.br)

Federal University of Health Sciences of Porto Alegre
Rua Sarmento Leite, 250, Porto Alegre, Rio Grande do Sul, Brazil

***Supplementary material***

| **Domain** | **Paradigm** | **Evaluated parameters** |  |
| --- | --- | --- | --- |
| **Anxiety-like** | Elevated plus maze/  Elevated zero maze | Time in the open arms/ area |  |
|  |  | Open arms entries | |
|  |  | Entries ratio (open/total; open/closed) | |
|  | Open field | Time in the center |  |
|  |  | Distance traveled in the center |  |
|  |  | Center crossings |  |
|  |  | Entries in the center |  |
|  |  | Latency to center |  |
|  | Light-dark box | Time in the light box |  |
|  | Novelty suppressed feeding | Latency time |  |
| **Depressive-like** | Forced swim | Immobility |  |
|  |  | Mobility |  |
|  | Tail suspension test | Immobility |  |
|  |  | Latency to immobilization |  |
|  | Sucrose/saccharin preference | Sucrose/saccharin preference |  |
|  |  | Sucrose consumption |  |
| **Cognition** | Barnes maze | Latency to target |  |
|  | Morris water maze | Time in the target quadrant |  |
|  |  | Frequency crossing platform |  |
|  |  | Escape latency |  |
|  | Y maze | Alternation |  |
|  |  | Time in the novel arm |  |
|  |  | Novel arm preference |  |
|  |  | Correct entries |  |
|  | Object location test | Discrimination index |  |
|  | Novel object recognition | Recognition or discrimination index |  |
|  |  | Time in the new object |  |
|  |  | Entries on the new object |  |
|  | Inhibitory avoidance | Latency |  |
|  | Social recognition | Social recognition index |  |
|  |  | Time spent exploring the novel animal |  |
|  |  | Exploration of the new animal |  |
| **Fear memory** | Context fear memory | Freezing |  |
|  | Cued fear memory | Freezing |  |

**Supplementary table 1.** Description of the parameters extracted from the studies. Data were extracted based on the outcomes reported for each behavioral paradigm, following the order listed in the table; when a parameter was not available, the next listed outcome was selected.

| doi | Author/ year | Strain | Disease model | | Sex | Age | Dose | Route | Number of administrations | Domain | Paradigm |
| --- | --- | --- | --- | --- | --- | --- | --- | --- | --- | --- | --- |
| 10.1186/s42238-020-00055-9 | Montoya et al., 2020 | C57BL/6J mice | Not a model | F | | Juvenile | 10 mg/kg | i.p. | Single | Fear memory | Contextual fear memory; Cued fear memory |
| 10.3389/fphar.2022.891842 | Hernández-Hernández et al., 2022 | Sprague–Dawley rats | Not a model | M | | Aged | 3, 10 and 30 mg/kg | i.p. | Repeated | Depressive-like | Forced swim |
| 10.1038/s41386-024-02005-y | Gom et al., 2025 | Long-Evans rats | Epilepsy | M | | Adult | 10 mg/mL | Vaporized | Single | Anxiety-like; Fear memory | Elevated plus maze; Cued fear memory |
| 10.1016/j.isci.2023.108327 | Samanta et al., 2023 | Lister Hooded rats | Not a model | M | | Juvenile | 120 mg/kg | Oral | Single | Cognition | Object location test |
| 10.3389/fnins.2024.1358555 | Yadav-Samudrala et al., 2024 | C57BL/6J mice | HIV-associated neurocognitive disorders | M&F | | Adult | 30 mg/kg | i.p. | Single | Anxiety-like; Cognition | Elevated plus maze; Novel object recognition |
| 10.3389/fphar.2023.1171646 | García-Gutiérrez et al., 2023 | CD1 mice | Stress | M | | Juvenile | 20 mg/kg | i.p. | Repeated | Anxiety-like; Depressive-like | Elevated plus maze; Light-dark box; Tail suspension; Sucrose consumption |
| 10.3390/ph15040473 | Austrich-Olivares et al., 2022 | CD1 mice | Not a model | M | | Adult | 10, 20 and 30 mg/kg | i.p. | Single | Anxiety-like; Depressive-like | Elevated plus maze; Light-dark box; Novelty suppressed feeding; Tail suspension |
| 10.3389/fnbeh.2021.711639 | Kaplan et al., 2021 | C57BL/6J mice | Not a model | M&F | | Juvenile; Adult | Adult: 5 and 10 mg/kg Juvenile: 20 mg/kg | i.p. | Single; Repeated | Anxiety-like; Cognition | Elevated plus maze; Open field; Barnes maze |
| 10.3389/fphar.2021.694510 | Gasparyan et al., 2021 | C57BL/6J mice | Stress | M | | Juvenile | 20 mg/kg | i.p. | Repeated | Anxiety-like; Fear memory | Elevated plus maze; Light-dark box; Contextual fear memory |
| 10.1038/s41380-024-02789-x | Jin et al., 2025 | C57BL/6J mice | Alzheimer | M | | Adut | 5 mg/kg | i.p. | Repeated | Anxiety-like; Cognition | Open field; Barnes maze; Morris water maze; Novel object recognition |
| 10.3390/ph14121269 | Silote et al., 2021 | Swiss mice; C57BL/6 mice; Flinders Sensitive Line rats | Not a model | M&F | | Adult | Mice: 3, 10 and 30 mg/kg Rats: 10, 30 and 60mg/kg | i.p. | Single | Anxiety-like; Depressive-like | Elevated plus maze; Forced swim; Tail suspension test |
| 10.1016/j.neulet.2020.135594 | Abame et al., 2021 | C57BL/6J mice | Not a model | M | | Juvenile | 10, 30 and 100 mg/kg | i.p. | Repeated | Depressive-like | Forced swim |
| 10.1007/s11064-023-04041-4 | Aghamiri et al., 2024 | Wistar rats | Topiramate-induced memory loss | M | | Adult | 1, 2.5, 5, 10, 20 and 40 mg/kg | i.p. | Single | Cognition | Inhibitory avoidance |
| 10.1016/j.euroneuro.2008.07.001 | Bitencourt et al., 2008 | Wistar rats | Not a model | M | | Adult | Fear conditioning 0.2, 1, 2 μg/μl Elevated plus maze: 2 ug/uL | CNS injection | Single | Anxiety-like; Fear memory | Elevated plus maze; Contextual fear memory |
| 10.3390/ijms24032052 | Bright et al., 2023 | Sprague-Dawley rats | Stress | M | | Adult | 10 mg/kg | i.p. | Repeated | Anxiety-like; Depressive-like | Open field; Forced swim |
| 10.1007/s00213-008-1168-x | Campos et al., 2008 | Wistar rats | Not a model | M | | Juvenile | 15, 30 and 60 nmol | CNS injection | Single | Anxiety-like | Elevated plus maze |
| 10.1016/j.jpsychires.2012.08.012 | Campos et al., 2012 | Wistar rats | Stress | M | | Adult | 5 mg/kg | i.p. | Single; Repeated | Anxiety-like | Elevated plus maze |
| 10.1016/j.neuroscience.2014.12.051 | Campos et al., 2015 | C57BL/6J mice | Cerebral malaria | F | | Juvenile | 30 mg/kg | i.p. | Repeated | Anxiety-like | Open field; Novel object recognition |
| 10.1016/j.brainres.2010.06.023 | Cassol-Jr et al., 2010 | Wistar rats | Sepsis | M | | Adult | 2.5, 5 and 10 mg/kg | i.p. | Repeated | Cognition | Inhibitory avoidance |
| 10.1016/j.neulet.2020.135020 | Chaves et al., 2020 | Wistar rats | Type 1 diabetes | M | | Adult | 3, 10 and 30 mg/kg | i.p. | Repeated | Anxiety-like; Depressive-like | Elevated plus maze; Forced swim |
| 10.1007/s11011-020-00667-3 | Chaves et al., 2021 | Wistar rats | Type 1 diabetes | M | | Adult | 30 mg/kg | i.p. | Repeated | Anxiety-like; Depressive-like | Elevated plus maze; Forced swim |
| 10.1007/s00213-014-3478-5 | Cheng et al., 2014a | C57BL/6J mice | Alzheimer | M | | Adult | 20 mg/kg | i.p. | Repeated | Anxiety-like; Cognition; Fear memory | Elevated plus maze; Novel object recognition; Social recognition; Contextual fear memory; Cued fear memory |
| 10.3233/JAD-140921 | Cheng et al., 2014b | C57BL/6J mice | Alzheimer | M | | Adult | 20 mg/kg | Oral | Repeated | Anxiety-like; Cognition; Fear memory | Elevated plus maze; Social recognition; Contextual fear memory; Cued fear memory |
| 10.3389/fphar.2022.931384 | Chesworth et al., 2022 | C57BL/6J mice | Alzheimer | F | | Adult | 20 mg/kg | Oral | Repeated | Anxiety-like; Fear memory | Elevated plus maze; Cued fear memory |
| 10.3389/fphar.2020.587604 | Coles et al., 2020 | C57BL/6J mice | Alzheimer | F | | Adult | 5 mg/kg | i.p. | Repeated | Anxiety-like; Cognition | Light-dark box; Novel object recognition |
| 10.1016/j.bbr.2021.113555 | Corrêa et al., 2022 | Wistar rats | Ovariectomy | F | | Adult | 10 mg/kg | i.p. | Repeated | Cognition | Inhibitory avoidance |
| 10.1016/j.neulet.2018.06.006 | Morais et al., 2018 | Wistar rats | Type 1 diabetes | M | | Adult | 0.3, 3, 10, 30 and 60 mg/kg | i.p. | Single; Repeated | Depressive-like | Forced swim |
| 10.23958/ijirms/vol07-i06/1418. | Ding et al., 2022 | C57BL/6J mice | Osteoarthritis | M&F | | Adult | 5 mg/kg | Subcutaneous | Repeated | Anxiety-like; Depressive-like | Light-dark box; Forced swim |
| 10.1089/can.2022.0011 | Drummond-Main et al., 2023 | Wistar rats | Not a model | M | | Juvenile | 30, 100 and 300 mg/kg | i.p. | Repeated | Cognition | Morris maze |
| 10.1177/02698811221125440 | Fabris et al., 2022 | Wistar rats | Not a model | M&F | | Adult | Single: 0.3, 3 and 30 mg/kg (M&F) Repeated: 0.3 mg/kg (F) | i.p. | Single; Repeated | Anxiety-like | Elevated plus maze; Open field |
| 10.3390/ijms23031076 | Faria et al., 2022 | Wistar rats | Type 1 diabetes | M | | Adult | 20 mg/kg | i.p. | Repeated | Cognition | Novel object recognition |
| 10.1016/j.bcp.2021.114433 | Florensa-Zanuy et al., 2021 | NMRI mice | LPS-induced inflammation | M | | Adult | 30 mg/kg | i.p. | Single | Anxiety-like; Depressive-like | Open field; Tail suspension test, Sucrose preference test |
| 10.1016/j.euroneuro.2013.10.012 | Fogaça et al., 2014 | Wistar rats | Stress; Not a model | M | | Adult | 15, 30 and 60 nmol | CNS injection | Single | Anxiety-like; Fear memory | Elevated plus maze; Contextual fear memory |
| 10.1016/j.neuropharm.2018.03.001 | Fogaça et al., 2018 | C57BL/6J mice | Stress | M | | Adult | 30 mg/kg | i.p. | Repeated | Anxiety-like | Elevated plus maze; Novelty suppressed feeding |
| 10.1016/j.biopha.2021.111813 | García-Baos et al., 2021 | C57BL/6J mice | Maternal alcohol consumption | M&F | | Juvenile | 20 mg/kg | i.p. | Repeated | Cognition | Y maze; Novel object recognition, Object location test |
| 10.1016/j.phrs.2023.106655 | Gasparyan et al., 2023 | C57BL/6J mice | Maternal alcohol consumption | M&F | | Juvenile | 30 mg/kg | i.p. | Repeated | Anxiety-like; Depressive-like; Cognition | Light-dark box; Novelty suppressed feeding; Tail suspension; Novel object recognition |
| 10.3897/folmed.65.e107259 | Georgieva-Kotetarova et al., 2023 | Wistar rats | LPS-induced inflammation | M | | Adult | 5 and 10mg/kg | Oral | Repeated | Cognition | Y maze; Novel object recognition; Inhibitory avoidance |
| 10.1016/j.schres.2015.01.015 | Gomes et al., 2015 | C57BL/6J mice | Schizophrenia | M | | Juvenile | 30 and 60 mg/kg | i.p. | Repeated | Cognition | Novel object recognition |
| 10.1016/j.pbb.2011.06.027 | Granjeiro et al., 2011 | Wistar rats | Stress | M | | Adult | 30 nmol | CNS injection | Single | Anxiety-like | Elevated plus maze |
| 10.1186/s10194-023-01589-y | Greco et al., 2023 | Sprague-Dawley rats | Pain | M | | Juvenile | Single: 15 and 30 mg/kg Repeated: 30 mg/kg | i.p. | Single; Repeated | Anxiety-like | Open field |
| 10.1097/j.pain.0000000000001386 | Gregorio et al., 2019 | Wistar rats | Pain | M | | Juvenile | 5 mg/kg | Subcutaneous | Repeated | Anxiety-like; Depressive-like | Elevated plus maze; Open field; Novelty suppressed feeding; Forced swim |
| 10.1007/BF02244012 | Guimarães et al., 1990 | Wistar rats | Not a model | M | | Adult | 2.5, 5, 10 and 20 mg/kg | i.p. | Single | Anxiety-like | Elevated plus maze |
| 10.1016/0306-3623(94)90027-2 | Guimarães et al., 1994 | Wistar rats | Not a model | M | | Adult | 5 mg/kg | i.p. | Single | Anxiety-like | Elevated plus maze |
| 10.1007/s00213-022-06132-6 | Han et al., 2022 | C57BL/6J mice | Stress | M | | Adult | 10 and 30 mg/kg | i.p. | Repeated | Anxiety-like; Fear memory | Elevated plus maze; Open field; Contextual fear memory |
| 10.1007/s11684-021-0896-8 | Hou et al., 2022 | C57BL/6J mice | Stress | M | | Adult | 5 and 10 mg/kg | i.p. | Repeated | Anxiety-like; Depressive-like | Elevated plus maze; Forced swim; Sucrose preference |
| 10.3390/molecules26195977 | Kruk-Slomka et al., 2021 | Swiss mice | Schizophrenia | M | | Juvenile | 1 and 5 mg/kg | i.p. | Single | Cognition | Inhibitory avoidance |
| 10.1016/j.neuropharm.2015.12.017 | Linge et al., 2016 | C57BL/6J mice | Depression | M | | Adult | Single:10 mg/kg Repeated: 50 mg/kg | i.p. | Single; Repeated | Anxiety-like; Depressive-like | Open field; Sucrose preference |
| 10.1017/S1461145709990605 | Long et al., 2010 | C57BL/6J mice | Not a model | M | | Adult | 1, 5, 10 and 50 mg/kg | i.p. | Repeated | Anxiety-like; Cognition | Open field; Light-dark box; Inhibitory avoidance |
| 10.1016/j.brainresbull.2021.06.017 | Malvestio et al., 2021 | Wistar rats | Pain | M | | Juvenile | 15, 30 and 60 nmol | CNS injection | Single | Depressive-like | Forced swim |
| 10.1007/s11011-022-01071-9 | Marçal et al., 2022 | BALB/c mice | Obesogenic diet | M | | Adult | 30 mg/kg | i.p. | Single; Repeated | Anxiety-like | Novelty suppressed feeding |
| 10.1016/j.bbr.2015.02.023 | Marinho et al., 2015 | Wistar rats | Stress | M | | Adult | 30 nmol | CNS injection | Single | Anxiety-like | Elevated plus maze |
| 10.1017/neu.2024.15 | Mattos et al., 2024 | Wistar rats | Parkinson | M | | Adult | 10 and 30mg/kg | i.p. | Repeated | Anxiety-like; Depressive-like; Novel object recognition | Open field; Novelty suppressed feeding; Forced swim; Novel object recognition |
| 10.1002/jnr.25384 | Mesiakaris et al., 2024 | C57BL/6J mice | Anxiety | M | | Adult | 50 mg/kg | i.p. | Single | Anxiety-like | Open field |
| 10.1007/s12035-021-02479-7 | Meyer et al., 2021 | Wistar rats | Cerebral ischemia | M | | Juvenile | 10 mg/kg | i.p. | Repeated | Cognition | Object location test |
| 10.1016/j.pnpbp.2016.11.005 | Mori et al., 2017 | C57BL/6J mice | Cerebral ischemia | M | | Adult | 10 mg/kg | i.p. | Repeated | Anxiety-like; Depressive-like; Cognition | Elevated zero maze; Forced swim; Y maze; Object location test |
| 10.1111/ejn.15134 | Mori et al., 2021 | C57BL/6J mice | Cerebral ischemia | M | | Adult | 10 mg/kg | i.p. | Repeated | Anxiety-like; Depressive-like; Cognition | Elevated zero maze; Forced swim; Y-maze |
| 10.1111/bph.14147 | Myers et al., 2019 | C57BL/6J mice | Not a model | M | | Juvenile | 5, 10 and 20 mg/kg | i.p. | Single | Cognition | Barnes maze |
| 10.1038/npp.2017.40 | Osborne et al., 2017 | Sprague-Dawley rats | Maternal immune activation | M | | Juvenile | 10 mg/kg | i.p. | Repeated | Cognition | Novel object recognition |
| 10.1016/j.neuropharm.2012.05.034 | Pazos et al., 2012 | Wistar rats | Hypoxia-ischemia | M&F | | Pre-weaning | 1 mg/kg | i.p. | Single | Cognition | Novel object recogntion |
| 10.3390/brainsci14100999 | Poudel et al., 2024 | Long-Evans rats | Chemotherapy | F | | Adult | 10 mg/kg | Oral | Repeated | Anxiety-like; Depressive-like; Cognition | Elevated plus maze; Open field; Sucrose preference; Novel object recognition |
| 10.1111/bph.14895 | Raymundi et al., 2020 | Wistar rats | Not a model | M | | Adult | 10 and 30 pmol | CNS injection | Single | Fear memory | Contextual fear memory |
| 10.1016/j.bbr.2006.05.016 | Resstel et al., 2006 | Wistar rats | Not a model | M | | Adult | 10 mg/kg | i.p. | Single | Fear memory | Contextual fear memory |
| 10.1111/j.1601-5215.2011.00579.x | Réus et al., 2011 | Wistar rats | Not a model | M | | Adult | 15, 30 and 60 mg/kg | i.p. | Single; Repeated | Depressive-like | Forced swim |
| 10.1016/j.bbi.2024.04.010 | Rodrigues et al., 2024 | Wistar rats | Maternal obesity | M&F | | Juvenile | 50 mg/kg | Oral | Repeated | Anxiety-like; Cognition | Elevated plus maze; Open field; Social recognition |
| 10.1016/j.neuroscience.2017.03.019 | Rossignoli et al., 2017 | Wistar rats | Not a model | M | | Adult | 2 ug/uL | CNS injection | Single | Fear memory | Contextual fear memory |
| 10.1016/j.pnpbp.2018.06.002 | Sales et al., 2018 | Swiss mice | Not a model | M | | Adult | 3, 7 and 10 mg/kg | i.p. | Single | Depressive-like | Forced swim |
| 10.1016/j.bbr.2016.01.033 | Sartim et al., 2016 | Wistar rats | Not a model | M | | Adult | 10, 30 and 60 nmol | CNS injection | Single | Depressive-like | Forced swim |
| 10.1177/0269881118784877 | Sartim et al., 2018 | Swiss mice | Not a model | M | | Adult | 10, 30 and 60 nmol | CNS injection | Single | Depressive-like | Forced swim |
| 10.1016/j.neuropharm.2021.108679 | Sartim et al., 2021 | Swiss mice | Not a model | M | | Adult | 3, 10 and 30 mg/kg | i.p. | Single | Depressive-like | Forced swim |
| 10.1007/s12640-014-9457-0 | Schiavon et al., 2014 | Swiss mice | Cerebral ischemia | M | | Juvenile | 3, 10 and 30 mg/kg | i.p. | Repeated | Cognition | Morris water maze |
| 10.1016/j.pnpbp.2015.06.017 | Schiavon et al., 2016 | Swiss mice | Not a model | M | | Juvenile | Single: 3, 10 and 30 mg/kg  Repeated: 3 and 30 mg/kg | i.p. | Single; Repeated | Anxiety-like; Depressive-like | Elevated plus maze; Tail suspension |
| 10.1016/j.physbeh.2018.12.019 | Shbiro et al., 2019 | Wistar-Kyoto rats; Flinders Sensitive Line rats | Depression | M&F | | Adult | 30 mg/kg | Oral | Single | Depressive-like | Forced swim; Sucrose preference |
| 10.1159/000443890 | Shoval et al., 2016 | Wistar-Kyoto rats | Depression | M | | Adult | 15, 30 and 45 mg/kg | Oral | Single | Anxiety-like; Depressive-like; Cognition | Elevated plus maze; Saccharin preference; Novel object recognition |
| 10.1016/j.neuropharm.2021.108712 | Silva-Cardoso et al., 2021 | Wistar rats | Neuropathic pain | M | | Adult | 0.3, 3, 10 and 30 mg/kg | i.p. | Repeated | Anxiety-like | Open field |
| 10.1016/j.pnpbp.2021.110367 | Sonego et al., 2021 | Swiss mice | Haloperidol-induced dysfunction | M | | Adult | 60 mg/kg | i.p. | Single; Repeated | Cognition | Novel object recognition |
| 10.1038/s41386-018-0282-7 | Szkudlarek et al., 2019 | Sprague-Dawley rats | Not a model | M | | Adult | 10, 100 and 500 ng/500 nl | CNS injection | Single | Anxiety-like; Cognition | Elevated plus maze; Y maze; Social recognition |
| 10.1007/s43440-021-00301-8 | Tito et al., 2021 | C57BL/6J mice | LPS-induced inflammation | M | | Adult | 30 mg/kg | i.p. | Single | Depressive-like | Forced swim |
| 10.1016/j.nlm.2018.02.009 | Uhernik et al., 2018 | C57BL/6J mice | Not a model | M | | Pre-weaning | 10 mg/kg | i.p. | Single | Fear memory | Context fear memory; Cued fear memory |
| 10.1038/s41390-024-03077-8 | Villa et al., 2024 | Wistar rats | Cerebral ischemia | M&F | | Pre-weaning | 5 mg/kg | i.p. | Single | Anxiety-like; Depressive-like | Open field; Forced swim; Tail suspension |
| 10.3233/JAD-191242 | Watt et al., 2020a | C57BL/6J mice | Alzheimer | M | | Adult | 50 mg/kg | i.p. | Repeated | Cognition | Social recognition |
| 10.1016/j.pbb.2020.172970 | Watt et al., 2020b | C57BL/6J mice | Alzheimer | M | | Adult | 50 mg/kg | i.p. | Repeated | Anxiety-like; Cognition; Fear memory | Elevated plus maze; Social recognition; Contextual fear memory; Cued fear memory |
| 10.1186/1478-811X-8-12 | Wolf et al., 2010 | C57BL/6J mice | Not a model | F | | Adult | NA | Oral | Repeated | Cognition | Morris water maze |
| 10.1016/j.etap.2019.103202 | Xu et al., 2019 | ICR mice | Stress | M | | Juvenile | Intravenous: 10mg/kg Oral: 10 and 100mg/kg | Intravenous; Oral | Repeated | Depressive-like | Forced swim |
| 10.1111/j.1476-5381.2009.00521.x | Zanelati et al., 2010 | Swiss mice | Not a model | M | | Adult | 3, 10, 30 and 100 mg/kg | i.p. | Single | Depressive-like | Forced swim |
| 10.1016/j.pbb.2019.05.002 | Zieba et al., 2019 | C57BL/6J mice | Fragile X Syndrome | M | | Adult | 5 and 20 mg/kg | i.p. | Single | Anxiety-like; Cognition | Elevated plus maze; Open field; Y maze; Inhibitory avoidance |
| 10.1007/s43440-021-00285-5 | Bis‑Humbert et al., 2021 | Sprague-Dawley rats | Stress | M | | Juvenile; Adults | 10 mg/kg | i.p. | Repeated | Anxiety-like; Depressive-like | Open field; Novelty suppressed feeding; Forced swim; Sucrose preference |
| 10.1007/s00213-020-05481-4 | Bis‑Humbert et al., 2020 | Sprague-Dawley rats | Not a model | M | | Juvenile; Adults | Juvenile: 3, 10 and 30 mg/kg Adults: 10 and 30 mg/kg | i.p. | Repeated | Axiety-like; Depressive-like | Open field; Forced swim; Sucrose preference |
| 10.3390/biom10050801 | Gáll et al., 2020 | Wistar rats | Stress | M | | Adult | 10 mg/kg | i.p. | Single; Repeated | Anxiety-like; Depressive-like | Elevated plus maze; Open field; Sucrose preference |
| 10.1159/000519534 | Amini et al., 2022 | Wistar rats | Alzheimer | M | | Adult | 120 mg/kg | Oral | Repeated | Cognition | Morris water maze |
| 10.1016/j.jpain.2023.10.001 | Rêgo et al., 2024 | Wistar rats | Pain | M&F | | Pre-weaning | 5 and 20 mg/kg | i.p. | Repeated | Anxiety-like | Elevated plus maze |
| 10.1016/j.bbi.2022.06.003 | Li et al., 2022 | C57BL/6J mice | LPS-induced inflammation | M | | Juvenile | 10 mg/kg | i.p. | Repeated | Anxiety-like | Elevated plus maze |
| 10.1016/j.neuroscience.2022.06.036 | Wang et al., 2022 | C57BL/6J mice | Parkinson | M | | Adult | 100 mg/kg | Oral | Repeated | Cognition | Morris water maze |
| 10.1016/j.neuropharm.2024.110196 | Rodrigues et al., 2025 | Wistar rats | Maternal obesity | M&F | | Adult | 50 mg/kg | Oral | Repeated | Anxiety-like; Cognition | Elevated plus maze; Social recognition |
| 10.1016/j.neuropharm.2024.110153 | Shen et al., 2024 | C57BL/6J mice | Pain | M | | Adult | 5 mg/kg | i.p. | Repeated | Anxiety-like | Open field; Tail suspension test |
| 10.1016/j.neuropharm.2024.109870 | Domingos et al., 2024 | Flinders Sensitive Line rats | Depression | M | | Adult | 10 mg/kg | i.p. | Repeated | Depressive-like | Forced swim |
| 10.1016/j.brainres.2024.148771 | Gava et al., 2024 | Wistar rats | Sepsis | M | | Adult | 10 mg/kg | i.p. | Repeated | Cognition | Inhibitory avoidance |
| 10.1016/j.euroneuro.2022.08.002 | Franzen et al., 2022 | Wistar rats | Not a model | F | | Adult | 10 mg/kg | i.p. | Single | Fear memory | Contextual fear |
| 10.1016/j.neuropharm.2023.109602 | Alonso et al., 2023 | C57BL/6J mice | Dravet syndrome | M&F | | Pre-weaning | 5 mg/kg | i.p. | Repeated | Cognition | Y maze |
| 10.1523/ENEURO.0417-22.2023 | Alvarez et al., 2023 | Wistar rats | Cerebral ischemia | M&F | | Pre-weaning | 0.1 and 1 mg/kg | i.p. | Repeated | Cognition | Novel object recongition |
| 10.1007/s12035-018-1456-3 | Aso et al., 2019 | C57BL/6J mice | Not a model | M | | Adult | 3 mg/kg | i.p. | Single | Anxiety-like; Cognition | Open field; Novel object recognition |
| 10.3389/fnbeh.2020.612624 | Cabral-Pereira et al., 2021 | GASH/Sal hamsters | Seizures | M | | Adult | 100 mg/kg | i.p. | Repeated | Anxiety-like | Open field |
| 10.1017/neu.2023.13 | Chaves et al., 2024 | Wistar rats | Type 1 diabetes | M | | Juvenile | 10, 30 and 60 mg/kg | i.p. | Repeated | Anxiety-like; Fear memory | Elevated plus maze; Contextual fear memory |
| 10.1016/j.pbb.2010.03.004. | El-Alfy et al., 2010 | Swiss mice | Not a model | M | | Adult | 20, 100 and 200 mg/kg | i.p. | Single | Depressive-like | Forced swim; Tail suspension test |
| 10.3390/biomedicines10081811 | Gáll et al., 2022 | Wistar rats | Epilepsy | M | | Adult | 60 mg/kg | Oral | Repeated | Anxiety-like; Cognition | Open field; Novel object recognition |
| 10.1186/s40779-024-00563-2 | Kodali et al., 2024 | Sprague-Dawley rats | Stress | M | | Adult | 20 mg/kg | Oral | Repeated | Depressive-like; Cognition | Sucrose preference; Novel object recogntion |
| 10.1038/s41398-022-01994-y | Ledesma-Corvi et al., 2022 | Sprague-Dawley rats | Stress | M&F | | Juvenile; Adult | 10 mg/kg | i.p. | Repeated | Anxiety-like; Depressive-like | Novelty suppressed feeding; Forced swim; Sucrose preference |
| 10.3390/ph16071003 | Macêdo-Souza et al., 2023 | Wistar rats | Pain | NA | | Adult | 5 mg/kg | i.p. | Repeated | Anxiety-like | Open field |
| 10.2174/1570159X21666230912101441 | Pérez-Valenzuela et al., 2024 | Sprague-Dawley rats | Stress | M&F | | Adult | 30 mg/kg | Oral | Repeated | Anxiety-like; Depressive-like; Cognition | Elevated plus maze; Forced swim; Sucrose preference; Social recognition |
| 10.1111/j.1476-5381.2008.00046.x | Resstel et al., 2009 | Wistar rats | Stress | M | | Adult | 10 mg kg | i.p. | Single | Anxiety-like | Elevated plus maze |
| 10.3389/fphar.2020.585096 | Silvestri et al., 2020 | CD1 mice | Colitis | M | | Adult | 1 mg/kg | Oral | Repeated | Anxiety-like; Cognition | Light-dark box; Novel object recognition |
| 10.3390/molecules26175354 | Stollenwerk et al., 2021 | C57BL/6J | Not a model | M | | Adult | 5 mg/kg | i.p. | Single | Anxiety-like | Elevated plus maze |
| 10.3389/fncel.2022.1010478 | Visini et al., 2022 | A/J mice | Schizophrenia | M | | Juvenile | 30 mg/kg | i.p. | Repeated | Anxiety-like; Fear memory | Open field; Cued fear memory |
| 10.3390/genes15040519 | Xie et al., 2024 | C57BL/6J mice | Stress | M | | Adult | 10 mg/kg | i.p. | Single | Anxiety-like; Cognition; Fear memory | Elevated plus maze; Cognition; Cued fear memory |
| 10.1097/FBP.0000000000000565 | Assareh et al., 2020 | C57BL/6J mice | Not a model | M | | NA | 1, 10, 30 and 100 mg/kg | i.p. | Single | Anxiety-like; Fear memory | Light-dark box; Contextual fear memory; Cued fear memory |
| 10.1177/0269881110389095 | Gomes et al., 2012 | Wistar rats | Not a model | M | | Adult | 15, 30 and 60 nmol | CNS injection | Single | Fear memory | Contextual fear memory |
| 10.1111/adb.12778 | Luján et al., 2020 | CD1 mice | Not a model | M | | Juvenile | 10 and 20 mg/kg | i.p. | Repeated | Anxiety-like; Cognition | Elevated plus maze; Novel object recognition |
| 10.1016/j.pnpbp.2021.110508 | Martin-Sánchez et al., 2022 | CD1 mice | Early-life stress | M&F | | Pre-weaning | 20 mg/kg | i.p. | Repeated | Anxiety-like; Depressive-like | Elevated plus maze; Tail suspension |
| 10.1016/j.bbr.2020.112627 | Sales et al., 2020 | Swiss mice | Not a model | M | | Adult | 3, 7 and 10 mg/kg | i.p. | Single | Depressive-like | Forced swim |
| 10.1089/can.2024.0086 | Novais Júnior et al., 2025 | Wistar rats | LPS-induced inflammation | M | | Adult | 15 and 30 mg/kg | Oral | Repeated | Anxiety-like; Depressive-like | Elevated plus maze; Open field; Forced swim |
| 10.1089/can.2023.0101 | Arnanz et al., 2024 | C57BL/6J mice | Alzheimer | M | | Adult | 0.273 mg/kg | i.p. | Repeated | Anxiety-like; Depressive-like; Cognition | Elevated plus maze; Open field; Tail suspension; Barnes maze |
| 10.1089/can.2022.0225 | Dinur et al., 2024 | C57BL/6J mice | LPS-induced inflammation | M&F | | Adult | Single: 5 mg/kg Repeated: 30 mg/kg | i.p. | Single; Repeated | Anxiety-like; Depressive-like | Elevated plus maze; Open field; Forced swim; Sucrose preference |
| 10.1089/neu.2023.0539 | Dong et al., 2024 | C57BL/6J mice | Traumatic brain injury | M | | Adult | 5 mg/kg | i.p. | Repeated | Cognition | Novel object recognition |
| 10.1097/j.pain.0000000000002960 | Sturaro et al., 2023 | C57BL/6J mice | Pain | M&F | | Adult | 30 mg/kg | i.p. | Single | Anxiety-like | Elevated plus maze; Light-dark box |

**Supplementary table 2.** Description and summary of the main characteristics of the included studies. i.p., intraperitoneal; M, male; F, female.

| **Anxiety-like behavior** |  |  |  | |
| --- | --- | --- | --- | --- |
| Authors | Paradigm | Hedges’ g | | SE |
| Gom et al., 2024 | Elevated plus maze | -2,76957 | 0,69752 | |
| Yadav-Samudrala et al., 2024 | Elevated plus maze | 0,023446 | 0,459486 | |
| García-Gutiérrez et al., 2023 | Elevated plus maze | -2,34254 | 0,537867 | |
| Austrich-Olivares et al., 2022 | Elevated plus maze | -4,23577 | 0,878459 | |
| Austrich-Olivares et al., 2022 | Elevated plus maze | -12,381 | 2,217356 | |
| Austrich-Olivares et al., 2022 | Elevated plus maze | -0,27366 | 0,473831 | |
| Kaplan et al., 2021 | Elevated plus maze | -1,3965 | 0,6548 | |
| Kaplan et al., 2021 | Elevated plus maze | -0,5632 | 0,5906 | |
| Kaplan et al., 2021 | Elevated plus maze | -0,10046 | 0,392496 | |
| Gasparyan et al., 2021 | Elevated plus maze | -2,04415 | 0,560254 | |
| Silote et al., 2021 | Elevated plus maze | -0,23114 | 0,501864 | |
| Silote et al., 2021 | Elevated plus maze | 0,430304 | 0,506432 | |
| Silote et al., 2021 | Elevated plus maze | -0,56363 | 0,510985 | |
| Silote et al., 2021 | Elevated plus maze | -0,23584 | 0,501941 | |
| Silote et al., 2021 | Elevated plus maze | -0,37335 | 0,50485 | |
| Silote et al., 2021 | Elevated plus maze | -1,06347 | 0,581166 | |
| Silote et al., 2021 | Elevated plus maze | -0,20752 | 0,501503 | |
| Silote et al., 2021 | Elevated plus maze | -0,08047 | 0,500226 | |
| Silote et al., 2021 | Elevated plus maze | -0,14583 | 0,500743 | |
| Silote et al., 2021 | Elevated plus maze | 0,46218 | 0,426523 | |
| Silote et al., 2021 | Elevated plus maze | 0,178644 | 0,434532 | |
| Silote et al., 2021 | Elevated plus maze | -0,1159 | 0,434009 | |
| Bitencourt et al., 2008 | Elevated plus maze | -2,27077 | 0,679628 | |
| Campos et al., 2008 | Elevated plus maze | -0,80202 | 0,581476 | |
| Campos et al., 2008 | Elevated plus maze | -1,19056 | 0,550928 | |
| Campos et al., 2008 | Elevated plus maze | -0,75125 | 0,60865 | |
| Campos et al., 2012 | Elevated plus maze | -0,62548 | 0,424466 | |
| Campos et al., 2012 | Elevated plus maze | -0,40297 | 0,584187 | |
| Chaves et al., 2020 | Elevated plus maze | -1,4504 | 0,660457 | |
| Chaves et al., 2020 | Elevated plus maze | -0,78309 | 0,602761 | |
| Chaves et al., 2020 | Elevated plus maze | -2,21923 | 0,757738 | |
| Chaves et al., 2021 | Elevated plus maze | -2,60521 | 0,815614 | |
| Cheng et al., 2014a | Elevated plus maze | -0,54397 | 0,425637 | |
| Cheng et al., 2014b | Elevated plus maze | -0,18523 | 0,501198 | |
| Chesworth et al., 2022 | Elevated plus maze | 0,41049 | 0,391559 | |
| Fabris et al., 2022 | Elevated plus maze | 0,315421 | 0,486634 | |
| Fabris et al., 2022 | Elevated plus maze | 0,701016 | 0,479669 | |
| Fabris et al., 2022 | Elevated plus maze | 0,281287 | 0,467109 | |
| Fabris et al., 2022 | Elevated plus maze | -1,53685 | 0,57669 | |
| Fabris et al., 2022 | Elevated plus maze | -0,69182 | 0,516461 | |
| Fabris et al., 2022 | Elevated plus maze | -1,56921 | 0,562586 | |
| Fabris et al., 2022 | Elevated plus maze | -1,13973 | 0,473781 | |
| Fabris et al., 2022 | Elevated plus maze | -2,06003 | 0,595788 | |
| Fabris et al., 2022 | Elevated plus maze | -0,62404 | 0,486751 | |
| Fabris et al., 2022 | Elevated plus maze | 0,010487 | 0,492809 | |
| Fabris et al., 2022 | Elevated plus maze | -1,20462 | 0,532434 | |
| Fogaça et al., 2014 | Elevated plus maze | 0,402969 | 0,47665 | |
| Fogaça et al., 2014 | Elevated plus maze | 1,42558 | 0,533349 | |
| Fogaça et al., 2014 | Elevated plus maze | 0,763698 | 0,489983 | |
| Fogaça et al., 2014 | Elevated plus maze | -2,003 | 0,727682 | |
| Fogaça et al., 2018 | Elevated plus maze | -1,41421 | 0,565614 | |
| Granjeiro et al., 2011 | Elevated plus maze | -1,07713 | 0,558136 | |
| Gregorio et al., 2019 | Elevated plus maze | -1,30757 | 0,524007 | |
| Guimarães et al., 1990 | Elevated plus maze | -1,58224 | 0,517911 | |
| Guimarães et al., 1990 | Elevated plus maze | -2,52 | 0,6108 | |
| Guimarães et al., 1990 | Elevated plus maze | -1,42279 | 0,505146 | |
| Guimarães et al., 1990 | Elevated plus maze | -0,69614 | 0,461744 | |
| Guimarães et al., 1994 | Elevated plus maze | -0,66473 | 0,50414 | |
| Han et al., 2022 | Elevated plus maze | -2,81202 | 0,848535 | |
| Han et al., 2022 | Elevated plus maze | -0,41196 | 0,584493 | |
| Hou et al., 2022 | Elevated plus maze | -3,22103 | 0,572228 | |
| Hou et al., 2023 | Elevated plus maze | -2,9977 | 0,549634 | |
| Marinho et al., 2015 | Elevated plus maze | 0,218047 | 0,536332 | |
| Poudel et al., 2024 | Elevated plus maze | -3,55332 | 1,242426 | |
| Rodrigues et al., 2024 | Elevated plus maze | -1,48492 | 0,509996 | |
| Rodrigues et al., 2024 | Elevated plus maze | 0,44609 | 0,492552 | |
| Schiavon et al., 2015 | Elevated plus maze | -0,01441 | 0,48592 | |
| Schiavon et al., 2015 | Elevated plus maze | -0,12555 | 0,459963 | |
| Schiavon et al., 2015 | Elevated plus maze | -0,80409 | 0,491959 | |
| Schiavon et al., 2015 | Elevated plus maze | 0,508529 | 0,366946 | |
| Schiavon et al., 2015 | Elevated plus maze | -0,19294 | 0,366857 | |
| Shoval et al., 2016 | Elevated plus maze | -0,06481 | 0,447342 | |
| Shoval et al., 2016 | Elevated plus maze | 0,454101 | 0,453454 | |
| Shoval et al., 2016 | Elevated plus maze | 1,525359 | 0,513238 | |
| Szkudlarek et al., 2019 | Elevated plus maze | 0,294594 | 0,45209 | |
| Szkudlarek et al., 2019 | Elevated plus maze | 0,090123 | 0,483751 | |
| Watt et al., 2020b | Elevated plus maze | 0,240705 | 0,394905 | |
| Zieba et al., 2019 | Elevated plus maze | -0,11635 | 0,408619 | |
| Zieba et al., 2019 | Elevated plus maze | -0,48623 | 0,414669 | |
| Gáll et al., 2020 | Elevated plus maze | -0,2993 | 0,503122 | |
| Gáll et al., 2020 | Elevated plus maze | -0,04456 | 0,474406 | |
| Rêgo et al., 2024 | Elevated plus maze | 0,275805 | 0,502652 | |
| Rêgo et al., 2024 | Elevated plus maze | -0,60692 | 0,512716 | |
| Li et al., 2022 | Elevated plus maze | -1,53534 | 0,468345 | |
| Rodrigues et al., 2025 | Elevated plus maze | -1,14002 | 0,485202 | |
| Rodrigues et al., 2025 | Elevated plus maze | -0,84 | 0,468221 | |
| Chaves et al., 2023 | Elevated plus maze | -0,84057 | 0,602307 | |
| Chaves et al., 2023 | Elevated plus maze | -0,65237 | 0,592508 | |
| Pérez-Valenzuela et al., 2024 | Elevated plus maze | 0,362061 | 0,590988 | |
| Pérez-Valenzuela et al., 2024 | Elevated plus maze | -0,63367 | 0,513846 | |
| Resstel et al., 2009 | Elevated plus maze | -2,1963 | 0,754465 | |
| Stollenwerk et al., 2021 | Elevated plus maze | -0,79835 | 0,611576 | |
| Xie et al., 2024 | Elevated plus maze | -2,01322 | 0,625855 | |
| Luján et al., 2019 | Elevated plus maze | -1,01197 | 0,435377 | |
| Luján et al., 2019 | Elevated plus maze | -1,23194 | 0,447866 | |
| Martin-Sánchez et al., 2021 | Elevated plus maze | -1,22515 | 0,490825 | |
| Novais Júnior et al., 2024 | Elevated plus maze | -0,15526 | 0,408908 | |
| Novais Júnior et al., 2024 | Elevated plus maze | 0,730781 | 0,422613 | |
| Arnanz et al., 2024 | Elevated plus maze | 0,171616 | 0,369141 | |
| Dinur et al., 2024 | Elevated plus maze | -0,49487 | 0,58763 | |
| Dinur et al., 2024 | Elevated plus maze | -0,1762 | 0,578664 | |
| Dinur et al., 2024 | Elevated plus maze | -0,01846 | 0,447224 | |
| Sturaro et al., 2023 | Elevated plus maze | -0,95014 | 0,432252 | |
| Sturaro et al., 2023 | Elevated plus maze | -0,2651 | 0,410167 | |
| Mori et al., 2016 | Elevated plus maze | -1,87865 | 0,49552 | |
| Mori et al., 2021 | Elevated plus maze | -1,36035 | 0,442179 | |
| Kaplan et al., 2021 | Open field | 0,261959 | 0,394022 | |
| Jin et al., 2024 | Open field | 0,149219 | 0,518358 | |
| Bright et al., 2023 | Open field | 0,341147 | 0,450746 | |
| Campos et al., 2014 | Open field | -2,43668 | 0,87401 | |
| Fabris et al., 2022 | Open field | 0,468244 | 0,443366 | |
| Fabris et al., 2022 | Open field | -0,27364 | 0,44949 | |
| Fabris et al., 2022 | Open field | 0,084944 | 0,447433 | |
| Fabris et al., 2022 | Open field | -0,71466 | 0,431502 | |
| Fabris et al., 2022 | Open field | -1,02915 | 0,496443 | |
| Fabris et al., 2022 | Open field | -0,33876 | 0,420628 | |
| Fabris et al., 2022 | Open field | -0,44067 | 0,453092 | |
| Fabris et al., 2022 | Open field | -0,4715 | 0,453937 | |
| Fabris et al., 2022 | Open field | -0,49957 | 0,444249 | |
| Florensa-Zanuy et al., 2021 | Open field | 0,251886 | 0,580031 | |
| Greco et al., 2023 | Open field | -0,8388 | 0,5607 | |
| Greco et al., 2023 | Open field | -0,9087 | 0,5651 | |
| Greco et al., 2023 | Open field | 0,5997 | 0,5481 | |
| Gregorio et al., 2019 | Open field | -1,99705 | 0,586822 | |
| Han et al., 2022 | Open field | -2,25262 | 0,762541 | |
| Han et al., 2022 | Open field | -2,01494 | 0,729293 | |
| Linge et al., 2016 | Open field | -2,13153 | 0,745324 | |
| Linge et al., 2016 | Open field | -0,76306 | 0,556275 | |
| Long et al., 2010 | Open field | -0,46796 | 0,542806 | |
| Long et al., 2010 | Open field | 0,491387 | 0,543649 | |
| Long et al., 2010 | Open field | 0,859859 | 0,561999 | |
| Long et al., 2010 | Open field | 0,040946 | 0,534586 | |
| Mattos et al., 2024 | Open field | -0,35316 | 0,431736 | |
| Mattos et al., 2024 | Open field | 0,406148 | 0,461334 | |
| Mesiakaris et al., 2024 | Open field | -0,79595 | 0,521678 | |
| Poudel et al., 2024 | Open field | -0,61693 | 0,729013 | |
| Rodrigues et al., 2024 | Open field | 0,372279 | 0,463798 | |
| Rodrigues et al., 2024 | Open field | -1,27013 | 0,50758 | |
| Silva-Cardoso et al., 2021 | Open field | -2,64723 | 0,703556 | |
| Silva-Cardoso et al., 2021 | Open field | -5,57851 | 1,156693 | |
| Silva-Cardoso et al., 2021 | Open field | -6,30369 | 1,280305 | |
| Silva-Cardoso et al., 2021 | Open field | -2,28354 | 0,657495 | |
| Villa et al., 2024 | Open field | 1,097 | 0,2086 | |
| Zieba et al., 2019 | Open field | 0,025719 | 0,408266 | |
| Zieba et al., 2019 | Open field | -0,11297 | 0,408597 | |
| Bis‑Humbert et al., 2021 | Open field | -0,4188 | 0,405 | |
| Bis‑Humbert et al., 2020 | Open field | 0,190793 | 0,460609 | |
| Bis‑Humbert et al., 2020 | Open field | 0,084179 | 0,471635 | |
| Bis‑Humbert et al., 2020 | Open field | -0,65698 | 0,514868 | |
| Bis‑Humbert et al., 2020 | Open field | -0,06348 | 0,534676 | |
| Bis‑Humbert et al., 2020 | Open field | -0,45957 | 0,542513 | |
| Gáll et al., 2020 | Open field | 0,12588 | 0,500554 | |
| Gáll et al., 2020 | Open field | 0,086004 | 0,47458 | |
| Shen et al., 2024 | Open field | -1,85039 | 0,493138 | |
| Aso et al., 2019 | Open field | 0,583986 | 0,547368 | |
| Cabral-Pereira et al., 2021 | Open field | -4,08429 | 1,071944 | |
| Gáll et al., 2022 | Open field | -0,08402 | 0,534791 | |
| Macêdo-Souza et al., 2023 | Open field | -0,55534 | 0,510668 | |
| Macêdo-Souza et al., 2023 | Open field | -1,79151 | 0,601833 | |
| Visini et al., 2022 | Open field | 0,755331 | 0,466444 | |
| Novais Júnior et al., 2024 | Open field | 0,35195 | 0,411625 | |
| Novais Júnior et al., 2024 | Open field | 0,464614 | 0,434321 | |
| Arnanz et al., 2024 | Open field | 0,249332 | 0,336754 | |
| Dinur et al., 2024 | Open field | -0,59457 | 0,457859 | |
| García-Gutiérrez et al., 2023 | Light-dark box | -0,9386 | 0,431688 | |
| Austrich-Olivares et al., 2022 | Light-dark box | -1,50038 | 0,539596 | |
| Austrich-Olivares et al., 2022 | Light-dark box | -2,09944 | 0,597667 | |
| Austrich-Olivares et al., 2022 | Light-dark box | -0,40555 | 0,476717 | |
| Gasparyan et al., 2021 | Light-dark box | -1,41563 | 0,504597 | |
| Coles et al., 2020 | Light-dark box | 0,672514 | 0,440953 | |
| Ding et al., 2022 | Light-dark box | -0,06696 | 0,44735 | |
| Gasparyan et al., 2023 | Light-dark box | -1,06572 | 0,45111 | |
| Gasparyan et al., 2023 | Light-dark box | -1,59865 | 0,500794 | |
| Long et al., 2010 | Light-dark box | -1,1567 | 0,583298 | |
| Long et al., 2010 | Light-dark box | -0,69917 | 0,552842 | |
| Long et al., 2010 | Light-dark box | 0,421101 | 0,54124 | |
| Long et al., 2010 | Light-dark box | -0,50446 | 0,544137 | |
| Silvestri et al., 2020 | Light-dark box | 0,112 | 0,447596 | |
| Assareh et al., 2020 | Light-dark box | 0,017099 | 0,385172 | |
| Assareh et al., 2020 | Light-dark box | -0,16274 | 0,36907 | |
| Assareh et al., 2020 | Light-dark box | -0,13539 | 0,410173 | |
| Assareh et al., 2020 | Light-dark box | -0,01601 | 0,392239 | |
| Sturaro et al., 2023 | Light-dark box | -0,15764 | 0,408928 | |
| Sturaro et al., 2023 | Light-dark box | 0,125555 | 0,40868 | |
| Austrich-Olivares et al., 2022 | Novelty suppressed feeding | -1,72978 | 0,560229 | |
| Austrich-Olivares et al., 2022 | Novelty suppressed feeding | -2,38241 | 0,629322 | |
| Austrich-Olivares et al., 2022 | Novelty suppressed feeding | 0,052025 | 0,471492 | |
| Fogaça et al., 2018 | Novelty suppressed feeding | -2,1959 | 0,646974 | |
| Gasparyan et al., 2023 | Novelty suppressed feeding | -1,1567 | 0,581106 | |
| Gasparyan et al., 2023 | Novelty suppressed feeding | -1,5928 | 0,610938 | |
| Gregorio et al., 2019 | Novelty suppressed feeding | -0,78056 | 0,490797 | |
| Marçal et al., 2022 | Novelty suppressed feeding | -0,26129 | 0,428363 | |
| Marçal et al., 2022 | Novelty suppressed feeding | -1,44254 | 0,482617 | |
| Mattos et al., 2024 | Novelty suppressed feeding | -0,71828 | 0,442722 | |
| Mattos et al., 2024 | Novelty suppressed feeding | -0,97208 | 0,483826 | |
| Bis‑Humbert et al., 2021 | Novelty suppressed feeding | -0,1029 | 0,4006 | |
| Ledesma-Corvi et al., 2024 | Novelty suppressed feeding | -0,26038 | 0,390073 | |
| Ledesma-Corvi et al., 2024 | Novelty suppressed feeding | 0 | 0,390868 | |
| Ledesma-Corvi et al., 2024 | Novelty suppressed feeding | 1,124542 | 0,464501 | |
| Ledesma-Corvi et al., 2024 | Novelty suppressed feeding | 0,537562 | 0,418977 | |
| **Depressive-like behavior** |  |  |  | |
| Hernández-Hernández et al., 2022 | Forced swim | 0,706193 | 0,490176 | |
| Hernández-Hernández et al., 2022 | Forced swim | -0,67261 | 0,460793 | |
| Hernández-Hernández et al., 2022 | Forced swim | 0,491455 | 0,444015 | |
| Silote et al., 2021 | Forced swim | -0,05358 | 0,556463 | |
| Silote et al., 2021 | Forced swim | -1,08247 | 0,558525 | |
| Silote et al., 2021 | Forced swim | -0,2066 | 0,558051 | |
| Silote et al., 2021 | Forced swim | -0,17773 | 0,460459 | |
| Silote et al., 2021 | Forced swim | 0,495375 | 0,587651 | |
| Silote et al., 2021 | Forced swim | 2,050345 | 0,771532 | |
| Silote et al., 2021 | Forced swim | 0,866365 | 0,608307 | |
| Silote et al., 2021 | Forced swim | -1,44815 | 0,660216 | |
| Abame et al., 2021 | Forced swim | -0,59131 | 0,457744 | |
| Abame et al., 2021 | Forced swim | -0,26555 | 0,449357 | |
| Abame et al., 2021 | Forced swim | -1,02931 | 0,47841 | |
| Bright et al., 2023 | Forced swim | -1,38208 | 0,502056 | |
| Chaves et al., 2020 | Forced swim | 0,251084 | 0,580014 | |
| Chaves et al., 2020 | Forced swim | -0,49253 | 0,587534 | |
| Chaves et al., 2020 | Forced swim | -1,27302 | 0,642324 | |
| Chaves et al., 2021 | Forced swim | -2,16359 | 0,749828 | |
| Morais et al., 2018 | Forced swim | 0 | 0,534522 | |
| Morais et al., 2018 | Forced swim | -1,04996 | 0,575011 | |
| Morais et al., 2018 | Forced swim | -0,20999 | 0,536201 | |
| Morais et al., 2018 | Forced swim | -0,97398 | 0,569536 | |
| Morais et al., 2018 | Forced swim | -0,93133 | 0,566622 | |
| Morais et al., 2018 | Forced swim | -0,8741 | 0,495602 | |
| Morais et al., 2018 | Forced swim | -0,2066 | 0,472789 | |
| Morais et al., 2018 | Forced swim | -0,16836 | 0,472324 | |
| Morais et al., 2018 | Forced swim | -1,70367 | 0,557773 | |
| Morais et al., 2018 | Forced swim | -0,98135 | 0,501713 | |
| Ding et al., 2022 | Forced swim | -0,41567 | 0,452448 | |
| Gregorio et al., 2019 | Forced swim | -0,2085 | 0,472815 | |
| Hou et al., 2022 | Forced swim | -2,64929 | 0,515937 | |
| Hou et al., 2022 | Forced swim | -3,95926 | 0,651294 | |
| Malvestio et al., 2021 | Forced swim | -0,57934 | 0,417334 | |
| Malvestio et al., 2021 | Forced swim | -1,11132 | 0,440757 | |
| Malvestio et al., 2021 | Forced swim | -1,01523 | 0,435546 | |
| Mattos et al., 2024 | Forced swim | -0,84922 | 0,448378 | |
| Mattos et al., 2024 | Forced swim | -0,97767 | 0,484132 | |
| Mori et al., 2016 | Forced swim | -1,14099 | 0,442448 | |
| Mori et al., 2021 | Forced swim | -1,00731 | 0,422353 | |
| Réus et al., 2011 | Forced swim | -1,4814 | 0,414692 | |
| Réus et al., 2011 | Forced swim | -1,00155 | 0,388579 | |
| Réus et al., 2011 | Forced swim | -0,78023 | 0,37954 | |
| Réus et al., 2011 | Forced swim | -1,07197 | 0,391873 | |
| Réus et al., 2011 | Forced swim | -0,84742 | 0,382068 | |
| Réus et al., 2011 | Forced swim | -0,54073 | 0,37213 | |
| Sales et al., 2018 | Forced swim | -0,0309 | 0,534559 | |
| Sales et al., 2018 | Forced swim | -0,69204 | 0,552477 | |
| Sales et al., 2018 | Forced swim | -1,68735 | 0,633827 | |
| Sartim et al., 2016 | Forced swim | -2,72713 | 0,767324 | |
| Sartim et al., 2016 | Forced swim | -1,32526 | 0,597733 | |
| Sartim et al., 2016 | Forced swim | -2,13674 | 0,686853 | |
| Sartim et al., 2016 | Forced swim | -0,33707 | 0,538836 | |
| Sartim et al., 2016 | Forced swim | -1,89684 | 0,657522 | |
| Sartim et al., 2016 | Forced swim | -3,32414 | 0,857905 | |
| Sartim et al., 2018 | Forced swim | -2,14663 | 0,74744 | |
| Sartim et al., 2018 | Forced swim | -1,71096 | 0,690278 | |
| Sartim et al., 2018 | Forced swim | -1,12191 | 0,628398 | |
| Sartim et al., 2021 | Forced swim | -1,12476 | 0,451492 | |
| Sartim et al., 2021 | Forced swim | -1,59298 | 0,483345 | |
| Sartim et al., 2021 | Forced swim | -0,57287 | 0,426523 | |
| Shbiro et al., 2019 | Forced swim | -1,5486 | 0,5064 | |
| Shbiro et al., 2019 | Forced swim | -1,19011 | 0,480662 | |
| Shbiro et al., 2019 | Forced swim | -0,57104 | 0,408938 | |
| Shbiro et al., 2019 | Forced swim | -0,01759 | 0,400329 | |
| Tito et al., 2021 | Forced swim | -1,52406 | 0,44886 | |
| Villa et al., 2024 | Forced swim | 0,9044 | 0,2041 | |
| Xu et al., 2019 | Forced swim | 1,365202 | 0,561389 | |
| Xu et al., 2019 | Forced swim | 0,035041 | 0,500043 | |
| Xu et al., 2019 | Forced swim | 1,180097 | 0,546522 | |
| Zanelati et al., 2010 | Forced swim | 1,003193 | 0,534026 | |
| Zanelati et al., 2010 | Forced swim | 0,062683 | 0,500137 | |
| Zanelati et al., 2010 | Forced swim | -1,07815 | 0,539108 | |
| Zanelati et al., 2010 | Forced swim | 1,028127 | 0,535681 | |
| Bis‑Humbert et al., 2021 | Forced swim | -0,4748 | 0,4063 | |
| Bis‑Humbert et al., 2021 | Forced swim | 0,416 | 0,4049 | |
| Bis‑Humbert et al., 2020 | Forced swim | -0,43512 | 0,465372 | |
| Bis‑Humbert et al., 2020 | Forced swim | -1,56044 | 0,544788 | |
| Bis‑Humbert et al., 2020 | Forced swim | 0,34163 | 0,504064 | |
| Bis‑Humbert et al., 2020 | Forced swim | -0,76366 | 0,556308 | |
| Bis‑Humbert et al., 2020 | Forced swim | -1,29431 | 0,594963 | |
| Domingos et al., 2024 | Forced swim | -0,96088 | 0,476638 | |
| El-Alfy et al., 2010 | Forced swim | -0,64136 | 0,549979 | |
| El-Alfy et al., 2010 | Forced swim | -0,94346 | 0,567439 | |
| El-Alfy et al., 2010 | Forced swim | -1,61071 | 0,62565 | |
| Ledesma-Corvi et al., 2024 | Forced swim | 0,3214 | 0,408221 | |
| Ledesma-Corvi et al., 2024 | Forced swim | 0,213675 | 0,389635 | |
| Ledesma-Corvi et al., 2024 | Forced swim | -1,09863 | 0,414718 | |
| Ledesma-Corvi et al., 2024 | Forced swim | -0,57134 | 0,39746 | |
| Ledesma-Corvi et al., 2024 | Forced swim | -0,31914 | 0,390746 | |
| Ledesma-Corvi et al., 2024 | Forced swim | -0,5447 | 0,396864 | |
| Ledesma-Corvi et al., 2024 | Forced swim | -0,60742 | 0,39831 | |
| Ledesma-Corvi et al., 2024 | Forced swim | -0,31067 | 0,392828 | |
| Pérez-Valenzuela et al., 2024 | Forced swim | -0,80778 | 0,564142 | |
| Pérez-Valenzuela et al., 2024 | Forced swim | -0,24546 | 0,502102 | |
| Sales et al., 2020 | Forced swim | -0,12455 | 0,447686 | |
| Sales et al., 2020 | Forced swim | -0,52095 | 0,455408 | |
| Sales et al., 2020 | Forced swim | -2,57407 | 0,616915 | |
| Novais Júnior et al., 2024 | Forced swim | -0,24433 | 0,409879 | |
| Novais Júnior et al., 2024 | Forced swim | -0,33865 | 0,403372 | |
| Dinur et al., 2024 | Forced swim | -0,67164 | 0,596148 | |
| Dinur et al., 2024 | Forced swim | -0,20887 | 0,579195 | |
| García-Gutiérrez et al., 2023 | Tail suspension test | -1,24983 | 0,448972 | |
| Austrich-Olivares et al., 2022 | Tail suspension test | -0,62097 | 0,483768 | |
| Austrich-Olivares et al., 2022 | Tail suspension test | -1,4606 | 0,536243 | |
| Austrich-Olivares et al., 2022 | Tail suspension test | -0,17549 | 0,472404 | |
| Silote et al., 2021 | Tail suspension test | -2,30353 | 0,825466 | |
| Silote et al., 2021 | Tail suspension test | -4,93599 | 1,470747 | |
| Silote et al., 2021 | Tail suspension test | -1,86453 | 0,762821 | |
| Silote et al., 2021 | Tail suspension test | -0,88625 | 0,586885 | |
| Silote et al., 2021 | Tail suspension test | -0,32573 | 0,538552 | |
| Silote et al., 2021 | Tail suspension test | 0,473071 | 0,594812 | |
| Silote et al., 2021 | Tail suspension test | -0,20785 | 0,519117 | |
| Silote et al., 2021 | Tail suspension test | -0,20701 | 0,501496 | |
| Silote et al., 2021 | Tail suspension test | 0,338073 | 0,50398 | |
| Silote et al., 2021 | Tail suspension test | -0,47829 | 0,434685 | |
| Silote et al., 2021 | Tail suspension test | -0,33178 | 0,420498 | |
| Silote et al., 2021 | Tail suspension test | -0,23577 | 0,429765 | |
| Florensa-Zanuy et al., 2021 | Tail suspension test | -1,32988 | 0,647933 | |
| Gasparyan et al., 2023 | Tail suspension test | -1,20575 | 0,372049 | |
| Gasparyan et al., 2023 | Tail suspension test | -1,93682 | 0,401424 | |
| Schiavon et al., 2015 | Tail suspension test | -2,16534 | 0,623847 | |
| Schiavon et al., 2015 | Tail suspension test | -1,43639 | 0,520207 | |
| Schiavon et al., 2015 | Tail suspension test | -0,99633 | 0,502616 | |
| Schiavon et al., 2015 | Tail suspension test | -1,15949 | 0,391297 | |
| Schiavon et al., 2015 | Tail suspension test | -1,32499 | 0,406001 | |
| Villa et al., 2024 | Tail suspension test | 0,2569 | 0,1951 | |
| Shen et al., 2024 | Tail suspension test | -0,9459 | 0,441791 | |
| El-Alfy et al., 2010 | Tail suspension test | -0,10037 | 0,534906 | |
| El-Alfy et al., 2010 | Tail suspension test | 0,094247 | 0,534861 | |
| El-Alfy et al., 2010 | Tail suspension test | 0,589026 | 0,547588 | |
| Martin-Sánchez et al., 2021 | Tail suspension test | -1,05309 | 0,508884 | |
| Arnanz et al., 2024 | Tail suspension test | 0,379906 | 0,309903 | |
| García-Gutiérrez et al., 2023 | Sucrose preference | -6,44409 | 1,046278 | |
| Florensa-Zanuy et al., 2021 | Sucrose preference | -1,23844 | 0,63901 | |
| Hou et al., 2022 | Sucrose preference | -1,93922 | 0,446699 | |
| Hou et al., 2022 | Sucrose preference | -2,94666 | 0,544573 | |
| Linge et al., 2016 | Sucrose preference | -1,06781 | 0,57635 | |
| Poudel et al., 2024 | Sucrose preference | -1,65885 | 0,852907 | |
| Shbiro et al., 2019 | Sucrose preference | -1,24923 | 0,492476 | |
| Shbiro et al., 2019 | Sucrose preference | -0,37566 | 0,451493 | |
| Shbiro et al., 2019 | Sucrose preference | -0,49725 | 0,454685 | |
| Shoval et al., 2016 | Sucrose preference | 0,155806 | 0,447953 | |
| Shoval et al., 2016 | Sucrose preference | -0,96964 | 0,475 | |
| Shoval et al., 2016 | Sucrose preference | -0,25932 | 0,449258 | |
| Bis‑Humbert et al., 2021 | Sucrose preference | 0,60824 | 0,401786 | |
| Bis‑Humbert et al., 2021 | Sucrose preference | 0,011872 | 0,392236 | |
| Bis‑Humbert et al., 2020 | Sucrose preference | 0,329858 | 0,462871 | |
| Bis‑Humbert et al., 2020 | Sucrose preference | -0,37268 | 0,475895 | |
| Bis‑Humbert et al., 2020 | Sucrose preference | -0,4304 | 0,506435 | |
| Bis‑Humbert et al., 2020 | Sucrose preference | -1,08938 | 0,577992 | |
| Bis‑Humbert et al., 2020 | Sucrose preference | -1,94879 | 0,663684 | |
| Gáll et al., 2020 | Sucrose preference | -0,2199 | 0,466158 | |
| Kodali et al., 2024 | Sucrose preference | -1,2137 | 0,4301 | |
| Ledesma-Corvi et al., 2024 | Sucrose preference | 0,3011 | 0,6941 | |
| Ledesma-Corvi et al., 2024 | Sucrose preference | 1,4171 | 0,7741 | |
| Pérez-Valenzuela et al., 2024 | Sucrose preference | -0,1278 | 0,540678 | |
| Pérez-Valenzuela et al., 2024 | Sucrose preference | -1,48982 | 0,592773 | |
| Dinur et al., 2024 | Sucrose preference | 0,268989 | 0,537273 | |
| Dinur et al., 2024 | Sucrose preference | -0,45996 | 0,453615 | |
| **Cognition** |  |  |  | |
| Kaplan et al., 2021 | Barnes maze | 0,156743 | 0,392874 | |
| Jin et al., 2024 | Barnes maze | -1,76899 | 0,669786 | |
| Myers et al., 2019 | Barnes maze | -0,37139 | 0,539754 | |
| Myers et al., 2019 | Barnes maze | -0,27986 | 0,5375 | |
| Myers et al., 2019 | Barnes maze | 0,468231 | 0,542815 | |
| Arnanz et al., 2024 | Barnes maze | -0,04253 | 0,353595 | |
| Jin et al., 2024 | Morris water maze | -3,80573 | 0,97636 | |
| Drummond-Main et al., 2023 | Morris water maze | -0,04003 | 0,527103 | |
| Drummond-Main et al., 2023 | Morris water maze | 0,799573 | 0,537603 | |
| Drummond-Main et al., 2023 | Morris water maze | 1,48692 | 0,664416 | |
| Schiavon et al., 2014 | Morris water maze | -0,16635 | 0,367171 | |
| Schiavon et al., 2014 | Morris water maze | -0,46469 | 0,343446 | |
| Schiavon et al., 2014 | Morris water maze | -0,03066 | 0,366609 | |
| Amini et al., 2022 | Morris water maze | -0,67794 | 0,551764 | |
| Wang et al., 2022 | Morris water maze | -3,0498 | 0,7584 | |
| Dong et al., 2024 | Morris water maze | -1,83866 | 0,570749 | |
| Wolf et al., 2010 | Morris water maze | 0,230755 | 0,448833 | |
| García-Baos et al., 2021 | Y maze | -0,90332 | 0,435505 | |
| Georgieva-Kotetarova et al., 2023 | Y maze | -1,77234 | 0,599846 | |
| Georgieva-Kotetarova et al., 2023 | Y maze | -1,62617 | 0,585191 | |
| Mori et al., 2016 | Y maze | -1,03555 | 0,436614 | |
| Mori et al., 2021 | Y maze | -1,23084 | 0,434328 | |
| Szkudlarek et al., 2019 | Y maze | 2,324687 | 0,662517 | |
| Zieba et al., 2019 | Y maze | 0,217582 | 0,409542 | |
| Zieba et al., 2019 | Y maze | 0,355892 | 0,4117 | |
| Alonso et al., 2023 | Y maze | -1,12123 | 0,628338 | |
| Samanta et al., 2023 | Object location test | -0,1711 | 0,2329 | |
| García-Baos et al., 2021 | Object location test | -1,03764 | 0,43054 | |
| Meyer et al., 2021 | Object location test | -0,85205 | 0,372723 | |
| Mori et al., 2016 | Object location test | -2,03945 | 0,509529 | |
| Mori et al., 2016 | Object location test | -0,15238 | 0,408883 | |
| Kodali et al., 2024 | Object location test | -0,93271 | 0,406798 | |
| Jin et al., 2024 | Novel object recognition | -2,34751 | 0,689369 | |
| Campos et al., 2014 | Novel object recognition | 0,364486 | 0,490355 | |
| Cheng et al., 2014a | Novel object recognition | -1,01019 | 0,445109 | |
| Coles et al., 2020 | Novel object recognition | -0,54857 | 0,436719 | |
| Faria et al., 2022 | Novel object recognition | -3,8117 | 1,021671 | |
| Faria et al., 2022 | Novel object recognition | -2,74086 | 0,837071 | |
| García-Baos et al., 2021 | Novel object recognition | 0,148918 | 0,4035 | |
| Gasparyan et al., 2023 | Novel object recognition | -1,01483 | 0,357018 | |
| Gasparyan et al., 2023 | Novel object recognition | -0,29637 | 0,326296 | |
| Georgieva-Kotetarova et al., 2023 | Novel object recognition | -1,25144 | 0,552042 | |
| Georgieva-Kotetarova et al., 2023 | Novel object recognition | -2,80249 | 0,724273 | |
| Gomes et al., 2015 | Novel object recognition | -1,15749 | 0,583362 | |
| Gomes et al., 2015 | Novel object recognition | -1,50609 | 0,614938 | |
| Mattos et al., 2024 | Novel object recognition | -0,25977 | 0,430105 | |
| Mattos et al., 2024 | Novel object recognition | -1,35049 | 0,507977 | |
| Osborne et al., 2017 | Novel object recognition | -1,48451 | 0,509963 | |
| Pazos et al., 2012 | Novel object recognition | -0,6612 | 0,37554 | |
| Poudel et al., 2024 | Novel object recognition | -1,02387 | 0,76593 | |
| Shoval et al., 2016 | Novel object recognition | -1,10286 | 0,482856 | |
| Shoval et al., 2016 | Novel object recognition | -0,87931 | 0,470184 | |
| Shoval et al., 2016 | Novel object recognition | -1,28296 | 0,494834 | |
| Sonego et al., 2021 | Novel object recognition | -0,7371 | 0,488734 | |
| Sonego et al., 2021 | Novel object recognition | -0,42306 | 0,452635 | |
| Alvarez et al., 2023 | Novel object recognition | -0,66073 | 0,460324 | |
| Alvarez et al., 2023 | Novel object recognition | -2,87287 | 0,651875 | |
| Aso et al., 2019 | Novel object recognition | 0,013274 | 0,534529 | |
| Gáll et al., 2022 | Novel object recognition | 1,1319 | 0,5813 | |
| Silvestri et al., 2020 | Novel object recognition | -0,0871 | 0,4474 | |
| Xie et al., 2024 | Novel object recognition | -1,08898 | 0,539869 | |
| Luján et al., 2019 | Novel object recognition | -1,94165 | 0,500918 | |
| Luján et al., 2019 | Novel object recognition | -2,2804 | 0,531865 | |
| Dong et al., 2024 | Novel object recognition | -1,38787 | 0,502491 | |
| Aghamiri et al., 2024 | Inhibitory avoidance | -0,27407 | 0,502619 | |
| Aghamiri et al., 2024 | Inhibitory avoidance | -0,54642 | 0,510331 | |
| Aghamiri et al., 2024 | Inhibitory avoidance | -0,58774 | 0,511934 | |
| Aghamiri et al., 2024 | Inhibitory avoidance | -1,76501 | 0,59909 | |
| Aghamiri et al., 2024 | Inhibitory avoidance | -2,73154 | 0,714735 | |
| Aghamiri et al., 2024 | Inhibitory avoidance | -3,26448 | 0,789024 | |
| Cassol-Jr et al., 2010 | Inhibitory avoidance | -10,239 | 0,3896 | |
| Cassol-Jr et al., 2010 | Inhibitory avoidance | -10,594 | 0,3913 | |
| Cassol-Jr et al., 2010 | Inhibitory avoidance | -10,594 | 0,3913 | |
| Corrêa et al., 2021 | Inhibitory avoidance | -1,37086 | 0,422444 | |
| Georgieva-Kotetarova et al., 2023 | Inhibitory avoidance | -3,07335 | 0,761716 | |
| Georgieva-Kotetarova et al., 2023 | Inhibitory avoidance | -4,24529 | 0,938117 | |
| Georgieva-Kotetarova et al., 2023 | Inhibitory avoidance | -2,15503 | 0,642152 | |
| Georgieva-Kotetarova et al., 2023 | Inhibitory avoidance | -2,32281 | 0,662287 | |
| Kruk-Slomka et al., 2021 | Inhibitory avoidance | -0,17729 | 0,501098 | |
| Kruk-Slomka et al., 2021 | Inhibitory avoidance | 0,510497 | 0,509029 | |
| Kruk-Slomka et al., 2021 | Inhibitory avoidance | -1,12088 | 0,542146 | |
| Kruk-Slomka et al., 2021 | Inhibitory avoidance | -1,21338 | 0,549063 | |
| Kruk-Slomka et al., 2021 | Inhibitory avoidance | -2,18188 | 0,645313 | |
| Kruk-Slomka et al., 2021 | Inhibitory avoidance | -3,03947 | 0,756949 | |
| Long et al., 2010 | Inhibitory avoidance | 0,05764 | 0,632616 | |
| Long et al., 2010 | Inhibitory avoidance | -0,35946 | 0,638686 | |
| Long et al., 2010 | Inhibitory avoidance | -0,2382 | 0,635199 | |
| Long et al., 2010 | Inhibitory avoidance | -0,66126 | 0,653299 | |
| Zieba et al., 2019 | Inhibitory avoidance | -0,16178 | 0,408964 | |
| Zieba et al., 2019 | Inhibitory avoidance | 0,020065 | 0,408259 | |
| Gava et al., 2024 | Inhibitory avoidance | -0,2463 | 0,4232 | |
| Cheng et al., 2014a | Social recognition | -0,60214 | 0,460328 | |
| Cheng et al., 2014b | Social recognition | -1,13992 | 0,543532 | |
| Rodrigues et al., 2024 | Social recognition | -1,30839 | 0,526713 | |
| Rodrigues et al., 2024 | Social recognition | 0,241979 | 0,487876 | |
| Szkudlarek et al., 2019 | Social recognition | -0,47797 | 0,500316 | |
| Watt et al., 2020a | Social recognition | -0,522 | 0,4831 | |
| Watt et al., 2020b | Social recognition | 0,0906 | 0,4567 | |
| Rodrigues et al., 2025 | Social recognition | -1,56008 | 0,530382 | |
| Rodrigues et al., 2025 | Social recognition | -0,12169 | 0,447665 | |
| Prez-Valenzuela et al., 2024 | Social recognition | -0,02524 | 0,517572 | |
| Prez-Valenzuela et al., 2024 | Social recognition | -0,14357 | 0,447841 | |
| **Fear-related memory** |  |  |  | |
| Montoya et al., 2020 | Contextual fear memory | -1,80429 | 0,489305 | |
| Gasparyan et al., 2021 | Contextual fear memory | -1,12332 | 0,484139 | |
| Gasparyan et al., 2021 | Contextual fear memory | -3,11338 | 0,681309 | |
| Bitencourt et al., 2008 | Contextual fear memory | -0,19312 | 0,421658 | |
| Bitencourt et al., 2008 | Contextual fear memory | 0,115968 | 0,41004 | |
| Bitencourt et al., 2008 | Contextual fear memory | -0,35373 | 0,403649 | |
| Cheng et al., 2014a | Contextual fear memory | 0,315626 | 0,420207 | |
| Cheng et al., 2014b | Contextual fear memory | -0,51597 | 0,509222 | |
| Fogaça et al., 2014 | Contextual fear memory | -2,09738 | 0,818295 | |
| Han et al., 2022 | Contextual fear memory | -2,00467 | 0,727908 | |
| Han et al., 2022 | Contextual fear memory | 0,167154 | 0,578532 | |
| Raymundi et al., 2020 | Contextual fear memory | -0,80156 | 0,558477 | |
| Raymundi et al., 2020 | Contextual fear memory | -2,56998 | 0,744891 | |
| Raymundi et al., 2020 | Contextual fear memory | -0,21916 | 0,519292 | |
| Raymundi et al., 2020 | Contextual fear memory | -2,22797 | 0,650796 | |
| Resstel et al., 2006 | Contextual fear memory | -4,35419 | 1,249784 | |
| Rossignoli et al., 2017 | Contextual fear memory | 0,189055 | 0,448301 | |
| Rossignoli et al., 2017 | Contextual fear memory | 0,17572 | 0,448153 | |
| Rossignoli et al., 2017 | Contextual fear memory | -0,4678 | 0,344102 | |
| Rossignoli et al., 2017 | Contextual fear memory | -0,79128 | 0,352846 | |
| Uhernik et al., 2018 | Contextual fear memory | 1,98771 | 0,504939 | |
| Watt et al., 2020b | Contextual fear memory | 0,397672 | 0,397498 | |
| Franzen et al., 2022 | Contextual fear memory | -1,72129 | 0,544575 | |
| Chaves et al., 2023 | Contextual fear memory | -0,21368 | 0,578996 | |
| Chaves et al., 2023 | Contextual fear memory | -0,20253 | 0,578829 | |
| Chaves et al., 2023 | Contextual fear memory | -0,33652 | 0,581422 | |
| Assareh et al., 2020 | Contextual fear memory | -0,59596 | 0,457908 | |
| Assareh et al., 2020 | Contextual fear memory | -0,54259 | 0,456096 | |
| Assareh et al., 2020 | Contextual fear memory | 0,085044 | 0,447434 | |
| Assareh et al., 2020 | Contextual fear memory | -0,26421 | 0,449336 | |
| Gomes et al., 2011 | Contextual fear memory | -0,72911 | 0,657709 | |
| Gomes et al., 2011 | Contextual fear memory | -4,81223 | 1,348808 | |
| Gomes et al., 2011 | Contextual fear memory | -5,77871 | 1,564171 | |
| Montoya et al., 2020 | Cued fear memory | -0,28715 | 0,410499 | |
| Gom et al., 2024 | Cued fear memory | 2,001286 | 0,55602 | |
| Cheng et al., 2014a | Cued fear memory | 0,3798 | 0,421447 | |
| Cheng et al., 2014b | Cued fear memory | 0,194628 | 0,501323 | |
| Chesworth et al., 2022 | Cued fear memory | -0,13728 | 0,382348 | |
| Uhernik et al., 2018 | Cued fear memory | 0,698457 | 0,421389 | |
| Watt et al., 2020b | Cued fear memory | 0,535422 | 0,4008 | |
| Visini et al., 2022 | Cued fear memory | 0,605208 | 0,460438 | |
| Xie et al., 2024 | Cued fear memory | -1,4291 | 0,56692 | |
| Assareh et al., 2020 | Cued fear memory | -0,53657 | 0,455902 | |
| Assareh et al., 2020 | Cued fear memory | -0,11947 | 0,447648 | |
| Assareh et al., 2020 | Cued fear memory | -1,08586 | 0,481804 | |
| Assareh et al., 2020 | Cued fear memory | -0,57149 | 0,457057 | |

**Supplementary table 3.** Individual effect size and corresponding standard errors for all comparisons included in the meta-analyses.

**Supplementary Fig. S1** Risk of bias analysis for each study conducted using the SYRCLE’s RoB tool. The colors of the symbols indicate the level of bias risk (green = low risk; yellow = unclear risk; red = high risk). The evaluated domains were: D1 = random sequence generation; D2 = baseline characteristics; D3 = allocation concealment; D4 = random housing; D5 = blinding; D6 = random outcome assessment; D7 = blinding of outcome assessment; D8 = incomplete outcome data; D9 = selective reporting; D10 = other bias.

***Anxiety-like behaviors***

**Supplementary Fig. S2** Forest plot of the meta-analysis comparing the performance of animals treated with CBD versus vehicle across the evaluated paradigms related to anxiety-like behavior. Results are shown including outliers and influential results. Negative values indicate a favorable effect of CBD, suggesting a potential anxiolytic effect across all tests. CBD, cannabidiol; CI, confidence interval; SMD, standardized mean difference.

**Supplementary Fig. S3** Forest plot of the meta-analysis comparing animals treated with CBD versus vehicle across anxiety-like behavioral paradigms, subgrouped by sex. Results are shown including outliers and influential cases (A) and after outlier removal (B). Negative values indicate a favorable effect of CBD, suggesting a potential anxiolytic effect. CBD, cannabidiol; CI, confidence interval; SMD, standardized mean difference.

**Supplementary Fig. S4** Forest plot of the meta-analysis comparing animals treated with CBD versus vehicle across anxiety-like behavioral paradigms, subgrouped by species. Results are shown including outliers and influential cases (A) and after outlier removal (B). Negative values indicate a favorable effect of CBD, suggesting a potential anxiolytic effect. CBD, cannabidiol; CI, confidence interval; SMD, standardized mean difference.

**Supplementary Fig. S5** Forest plots comparing CBD versus vehicle in anxiety-like behavioral paradigms, subgrouped by dose. Intraperitoneal administration is shown including outliers (A) and after removal (B), and oral administration including outliers (C) and after removal (D). Negative values indicate an anxiolytic effect of CBD. CBD, cannabidiol; CI, confidence interval; SMD, standardized mean difference.

**Supplementary Fig. S6** Forest plot of the meta-analysis comparing the performance of animals treated with CBD versus vehicle in anxiety-like paradigms, stratified by experimental model. Results are shown including outliers and influential results. Negative values indicate a favorable effect of CBD, suggesting a potential anxiolytic effect across all tests. CBD, cannabidiol; CI, confidence interval; SMD, standardized mean difference.

**Supplementary Fig. S7** Forest plot of the meta-analysis comparing the performance of animals treated with CBD versus vehicle subgrouped by disease model for each of the behavior tests included. Outliers and influential results were removed prior to analysis. Negative values indicate a favorable effect of CBD, suggesting a potential anxiolytic effect across all tests. CBD, cannabidiol; CI, confidence interval; DF, degrees of freedom; SMD, standardized mean difference

**Supplementary Fig. S8** Forest plot of the meta-analysis comparing the performance of animals treated with CBD versus vehicle in each anxiety-like paradigm, stratified by experimental model. Results are shown including outliers and influential results. Negative values indicate a favorable effect of CBD, suggesting a potential anxiolytic effect across all tests. CBD, cannabidiol; CI, confidence interval; LPS, lipopolysaccharide; SMD, standardized mean difference.

**Supplementary Fig. S9** Funnel plot of anxiety-like behavior in included studies, shown before (top) and after (bottom) trim-and-fill with outlier exclusion to evaluate potential publication bias.

***Depressive-like behaviors***

**Supplementary Fig. S10** Forest plot of the meta-analysis comparing the performance of animals treated with CBD versus vehicle across the evaluated paradigms related to depressive-like behavior. Results are shown including outliers and influential results. Negative values indicate a favorable effect of CBD, suggesting a potential antidepressant effect across all tests. CBD, cannabidiol; CI, confidence interval; SMD, standardized mean difference.

**Supplementary Fig. S11** Forest plot of the meta-analysis comparing animals treated with CBD versus vehicle across depressive-like behavioral paradigms, subgrouped by sex. Results are shown including outliers and influential cases (A) and after outlier removal (B). Negative values indicate a favorable effect of CBD, suggesting a potential anxiolytic effect. CBD, cannabidiol; CI, confidence interval; SMD, standardized mean difference.

**Supplementary Fig. S12** Forest plot of the meta-analysis comparing animals treated with CBD versus vehicle across depressive-like behavioral paradigms, subgrouped by species. Results are shown including outliers and influential cases (A) and after outlier removal (B). Negative values indicate a favorable effect of CBD, suggesting a potential anxiolytic effect. CBD, cannabidiol; CI, confidence interval; SMD, standardized mean difference.

**Supplementary Fig. S13** Forest plot of the meta-analysis comparing animals treated with CBD versus vehicle across depressive-like behavioral paradigms, subgrouped by dose. Intraperitoneal administration is shown including outliers (A) and after removal (B), and oral administration including outliers (C) and after removal (D). Negative values indicate a favorable effect of CBD, suggesting a potential anxiolytic effect. CBD, cannabidiol; CI, confidence interval; SMD, standardized mean difference.

**Supplementary Fig. S14** Forest plot of the meta-analysis comparing the performance of animals treated with CBD versus vehicle in depressive-like behavior tests stratified by disease model. Results are shown including outliers and influential results. Negative values indicate a favorable effect of CBD, suggesting a potential antidepressant effect across all tests. CBD, cannabidiol; CI, confidence interval; SMD, standardized mean difference.

**Supplementary Fig. S15** Forest plot of the meta-analysis comparing the performance of animals treated with CBD versus vehicle subgrouped by disease model for each of the depressive-like behavioral tests included. Outliers and influential results were removed prior to analysis. Negative values indicate a favorable effect of CBD, suggesting a potential antidepressant effect across all tests. CBD, cannabidiol; CI, confidence interval; DF, degrees of freedom; SMD, standardized mean difference

**Supplementary Fig. S16** Forest plot of the meta-analysis comparing the performance of animals treated with CBD versus vehicle in each depressive-like paradigm, stratified by experimental model. Results are shown including outliers and influential results. Negative values indicate a favorable effect of CBD, suggesting a potential antidepressant effect across all tests. CBD, cannabidiol; CI, confidence interval; LPS, lipopolysaccharide; SMD, standardized mean difference.

**Supplementary Fig. S17** Funnel plot of depressive-like behavior in included studies, shown before (top) and after (bottom) trim-and-fill with outlier exclusion to evaluate potential publication bias.

***Memory and cognition***

**Supplementary Fig. S18** Forest plot of the meta-analysis comparing the performance of animals treated with CBD versus vehicle across the evaluated paradigms related to memory and cognition. Results are shown including outliers and influential results. Negative values indicate a favorable effect of CBD, suggesting reduced cognitive deficits. CBD, cannabidiol; CI, confidence interval; SMD, standardized mean difference.

**Supplementary Fig. S19** Forest plot of the meta-analysis comparing animals treated with CBD versus vehicle across the evaluated paradigms related to memory and cognition, subgrouped by sex. Results are shown including outliers and influential cases (A) and after outlier removal (B). Negative values indicate a favorable effect of CBD, suggesting reduced cognitive deficits. CBD, cannabidiol; CI, confidence interval; SMD, standardized mean difference.

**Supplementary Fig. S20** Forest plot of the meta-analysis comparing animals treated with CBD versus vehicle across the evaluated paradigms related to memory and cognition, subgrouped by species. Results are shown including outliers and influential cases (A) and after outlier removal (B). Negative values indicate a favorable effect of CBD, suggesting reduced cognitive deficits. CBD, cannabidiol; CI, confidence interval; SMD, standardized mean difference.

**Supplementary Fig. S21** Forest plot of the meta-analysis comparing animals treated with CBD versus vehicle across the evaluated paradigms related to memory and cognition, subgrouped by dose. Intraperitoneal administration is shown including outliers (A) and after removal (B), and oral administration including outliers (C) and after removal (D). Negative values indicate a favorable effect of CBD, suggesting reduced cognitive deficits. CBD, cannabidiol; CI, confidence interval; SMD, standardized mean difference.

**Supplementary Fig. S22** Forest plot of the meta-analysis comparing the performance of animals treated with CBD versus vehicle in cognitive tests stratified by disease model. Results are shown including outliers and influential results. Negative values indicate a favorable effect of CBD, suggesting reduced cognitive deficits. CBD, cannabidiol; CI, confidence interval; SMD, standardized mean difference.

**Supplementary Fig. S23** Forest plot of the meta-analysis comparing the performance of animals treated with CBD versus vehicle subgrouped by disease model for each of the cognitive tests included. Outliers and influential results were removed prior to analysis. Negative values indicate a beneficial effect of CBD, reflecting better performance relative to vehicle-treated animals. CBD, cannabidiol; CI, confidence interval; DF, degrees of freedom; SMD, standardized mean difference

**Supplementary Fig. S24** Forest plot of the meta-analysis comparing the performance of animals treated with CBD versus vehicle in each cognitive test, stratified by experimental model. Results are shown including outliers and influential results. Negative values indicate a favorable effect of CBD, suggesting reduced cognitive deficits. CBD, cannabidiol; CI, confidence interval; LPS, lipopolysaccharide; SMD, standardized mean difference.

**Supplementary Fig. S25** Funnel plot of included cognitive and memory studies, shown before (top) and after (bottom) trim-and-fill with outlier exclusion to evaluate potential publication bias.

***Fear memory***

**Supplementary Fig. S26** Forest plot of the meta-analysis comparing the performance of animals treated with CBD versus vehicle across the evaluated paradigms of fear-related memory. Results are shown including outliers and influential results. Negative values indicate a beneficial effect of CBD, reflecting reduced fear responses to the aversive stimulus. CBD, cannabidiol; CI, confidence interval; SMD, standardized mean difference.

**Supplementary Fig. S27** Forest plot of the meta-analysis comparing animals treated with CBD versus vehicle across the evaluated paradigms of fear-related memory, subgrouped by sex. Results are shown including outliers and influential cases (A) and after outlier removal (B). Negative values indicate a beneficial effect of CBD, reflecting reduced fear responses to the aversive stimulus. CBD, cannabidiol; CI, confidence interval; SMD, standardized mean difference.

**Supplementary Fig. S28** Forest plot of the meta-analysis comparing animals treated with CBD versus vehicle across the evaluated paradigms of fear-related memory, subgrouped by species. Results are shown including outliers and influential cases (A) and after outlier removal (B). Negative values indicate a beneficial effect of CBD, reflecting reduced fear responses to the aversive stimulus. CBD, cannabidiol; CI, confidence interval; SMD, standardized mean difference.

**Supplementary Fig. S29** Forest plot of the meta-analysis comparing animals treated with CBD versus vehicle across the evaluated paradigms of fear-related memory, subgrouped by species. Intraperitoneal administration is shown including outliers (A) and after removal (B). Negative values indicate a beneficial effect of CBD, reflecting reduced fear responses to the aversive stimulus. CBD, cannabidiol; CI, confidence interval; SMD, standardized mean difference.

**Supplementary Fig. S30** Forest plot of the meta-analysis comparing the performance of animals treated with CBD versus vehicle in fear-related memory stratified by disease model. Results are shown including outliers and influential results. Negative values indicate a beneficial effect of CBD, reflecting reduced fear responses to the aversive stimulus. CBD, cannabidiol; CI, confidence interval; SMD, standardized mean difference.

**Supplementary Fig. S31** Forest plot of the meta-analysis comparing the performance of animals treated with CBD versus vehicle subgrouped by disease model for each of the fear-memory related tests included. Outliers and influential results were removed prior to analysis. Negative values indicate a beneficial effect of CBD, reflecting a reduced aversive response when animals are exposed to the aversive stimulus. CBD, cannabidiol; CI, confidence interval; DF, degrees of freedom; SMD, standardized mean difference

**Supplementary Fig. S32** Forest plot of the meta-analysis comparing the performance of animals treated with CBD versus vehicle in each fear-related memory test, stratified by experimental model. Results are shown including outliers and influential results. Negative values indicate a beneficial effect of CBD, reflecting reduced fear responses to the aversive stimulus. CBD, cannabidiol; CI, confidence interval; LPS, lipopolysaccharide; SMD, standardized mean difference.

**Supplementary Fig. S33** Funnel plot of fear-memory studies, shown before (top) and after (bottom) trim-and-fill with outlier exclusion to assess potential publication bias.

# PRISMA 2020 Main Checklist

| **Topic** | **No.** | **Item** | **Location where item is reported** |
| --- | --- | --- | --- |
| **TITLE** |  |  |  |
| **Title** | 1 | Identify the report as a systematic review. | Page 1, Line 1 |
| **ABSTRACT** |  |  |  |
| **Abstract** | 2 | See the PRISMA 2020 for Abstracts checklist |  |
| **INTRODUCTION** |  |  |  |
| **Rationale** | 3 | Describe the rationale for the review in the context of existing knowledge. | Introduction, L 65-74; L87-95 |
| **Objectives** | 4 | Provide an explicit statement of the objective(s) or question(s) the review addresses. | L 89-93 |
| **METHODS** |  |  |  |
| **Eligibility criteria** | 5 | Specify the inclusion and exclusion criteria for the review and how studies were grouped for the syntheses. | L 126-148; L 179-183 |
| **Information sources** | 6 | Specify all databases, registers, websites, organisations, reference lists and other sources searched or consulted to identify studies. Specify the date when each source was last searched or consulted. | L 122-124 |
| **Search strategy** | 7 | Present the full search strategies for all databases, registers and websites, including any filters and limits used. | L 107-124 |
| **Selection process** | 8 | Specify the methods used to decide whether a study met the inclusion criteria of the review, including how many reviewers screened each record and each report retrieved, whether they worked independently, and if applicable, details of automation tools used in the process. | L 126-157 |
| **Data collection process** | 9 | Specify the methods used to collect data from reports, including how many reviewers collected data from each report, whether they worked independently, any processes for obtaining or confirming data from study investigators, and if applicable, details of automation tools used in the process. | L 159-170; L 172-187 |
| **Data items** | 10a | List and define all outcomes for which data were sought. Specify whether all results that were compatible with each outcome domain in each study were sought (e.g. for all measures, time points, analyses), and if not, the methods used to decide which results to collect. | L 172-175; Supplementary table 1 |
|  | 10b | List and define all other variables for which data were sought (e.g. participant and intervention characteristics, funding sources). Describe any assumptions made about any missing or unclear information. | L 184-187 |
| **Study risk of bias assessment** | 11 | Specify the methods used to assess risk of bias in the included studies, including details of the tool(s) used, how many reviewers assessed each study and whether they worked independently, and if applicable, details of automation tools used in the process. | L 159-170 |
| **Effect measures** | 12 | Specify for each outcome the effect measure(s) (e.g. risk ratio, mean difference) used in the synthesis or presentation of results. | L 189-200 |
| **Synthesis methods** | 13a | Describe the processes used to decide which studies were eligible for each synthesis (e.g. tabulating the study intervention characteristics and comparing against the planned groups for each synthesis (item 5)). | L 179-183 |
|  | 13b | Describe any methods required to prepare the data for presentation or synthesis, such as handling of missing summary statistics, or data conversions. | L 184-186; 190-198 |
|  | 13c | Describe any methods used to tabulate or visually display results of individual studies and syntheses. | L 184-187; 190-198 |
|  | 13d | Describe any methods used to synthesize results and provide a rationale for the choice(s). If meta-analysis was performed, describe the model(s), method(s) to identify the presence and extent of statistical heterogeneity, and software package(s) used. | L 189-221 |
|  | 13e | Describe any methods used to explore possible causes of heterogeneity among study results (e.g. subgroup analysis, meta-regression). | L 199-221 |
|  | 13f | Describe any sensitivity analyses conducted to assess robustness of the synthesized results. | L 209-214 |
| **Reporting bias assessment** | 14 | Describe any methods used to assess risk of bias due to missing results in a synthesis (arising from reporting biases). | L 159-170 |
| **Certainty assessment** | 15 | Describe any methods used to assess certainty (or confidence) in the body of evidence for an outcome. | L 159-170 |
| **RESULTS** |  |  |  |
| **Study selection** | 16a | Describe the results of the search and selection process, from the number of records identified in the search to the number of studies included in the review, ideally using a flow diagram. | L 223-241 |
|  | 16b | Cite studies that might appear to meet the inclusion criteria, but which were excluded, and explain why they were excluded. | L 227-235 |
| **Study characteristics** | 17 | Cite each included study and present its characteristics. | Supplementary table 2. |
| **Risk of bias in studies** | 18 | Present assessments of risk of bias for each included study. | Supplementary Fig. S1 |
| **Results of individual studies** | 19 | For all outcomes, present, for each study: (a) summary statistics for each group (where appropriate) and (b) an effect estimate and its precision (e.g. confidence/credible interval), ideally using structured tables or plots. | Supplementary table 3. |
| **Results of syntheses** | 20a | For each synthesis, briefly summarise the characteristics and risk of bias among contributing studies. | L 256-619; Fig. 1; Supplementary table 2; Supplementary Fig. S1 |
|  | 20b | Present results of all statistical syntheses conducted. If meta-analysis was done, present for each the summary estimate and its precision (e.g. confidence/credible interval) and measures of statistical heterogeneity. If comparing groups, describe the direction of the effect. | L 256-619; Fig 1-5 |
|  | 20c | Present results of all investigations of possible causes of heterogeneity among study results. | L 256-619 |
|  | 20d | Present results of all sensitivity analyses conducted to assess the robustness of the synthesized results. | L 256-619; Fig 1-5; Supplementary Fig. S2-S33 |
| **Reporting biases** | 21 | Present assessments of risk of bias due to missing results (arising from reporting biases) for each synthesis assessed. | L 256-619 |
| **Certainty of evidence** | 22 | Present assessments of certainty (or confidence) in the body of evidence for each outcome assessed. | L 256-619 |
| **DISCUSSION** |  |  |  |
| **Discussion** | 23a | Provide a general interpretation of the results in the context of other evidence. | L621-910 |
|  | 23b | Discuss any limitations of the evidence included in the review. | L 912-932 |
|  | 23c | Discuss any limitations of the review processes used. | L 928-932 |
|  | 23d | Discuss implications of the results for practice, policy, and future research. | L 920-927 |
| **OTHER INFORMATION** |  |  |  |
| **Registration and protocol** | 24a | Provide registration information for the review, including register name and registration number, or state that the review was not registered. | L 99-101 |
|  | 24b | Indicate where the review protocol can be accessed, or state that a protocol was not prepared. | L99-101 |
|  | 24c | Describe and explain any amendments to information provided at registration or in the protocol. | NA |
| **Support** | 25 | Describe sources of financial or non-financial support for the review, and the role of the funders or sponsors in the review. | L941-945 |
| **Competing interests** | 26 | Declare any competing interests of review authors. | L946-947 |
| **Availability of data, code and other materials** | 27 | Report which of the following are publicly available and where they can be found: template data collection forms; data extracted from included studies; data used for all analyses; analytic code; any other materials used in the review. | L948-950 |

**Supplementary PRISMA 2020 Checklist***. From:* Page MJ, McKenzie JE, Bossuyt PM, Boutron I, Hoffmann TC, Mulrow CD, et al. The PRISMA 2020 statement: an updated guideline for reporting systematic reviews. MetaArXiv. 2020, September 14. DOI: 10.31222/osf.io/v7gm2. For more information, visit: <www.prisma-statement.org>

**Supplementary References**

1.	Montoya ZT, Uhernik AL, Smith JP. Comparison of cannabidiol to citalopram in targeting fear memory in female mice. J Cannabis Res. 2020;2:48.

2.	Hernández-Hernández E, García-Fuster MJ. Dose-Dependent Antidepressant-Like Effects of Cannabidiol in Aged Rats. Front Pharmacol. 2022;13:891842.

3.	Gom RC, Wickramarachchi P, George AG, Lightfoot SHM, Newton-Gunderson D, Hill MN, et al. Phytocannabinoids restore seizure-induced alterations in emotional behaviour in male rats. Neuropsychopharmacology. 2025;50:751–761.

4.	Samanta A, Aleman-Zapata A, Agarwal K, Özsezer P, Alonso A, van der Meij J, et al. CBD lengthens sleep but shortens ripples and leads to intact simple but worse cumulative memory. iScience. 2023;26:108327.

5.	Yadav-Samudrala BJ, Gorman BL, Barmada KM, Ravula HP, Huguely CJ, Wallace ED, et al. Effects of acute cannabidiol on behavior and the endocannabinoid system in HIV-1 Tat transgenic female and male mice. Front Neurosci. 2024;18:1358555.

6.	García-Gutiérrez MS, Navarro D, Austrich-Olivares A, Manzanares J. Unveiling behavioral and molecular neuroadaptations related to the antidepressant action of cannabidiol in the unpredictable chronic mild stress model. Front Pharmacol. 2023;14:1171646.

7.	Austrich-Olivares A, García-Gutiérrez MS, Illescas L, Gasparyan A, Manzanares J. Cannabinoid CB1 receptor involvement in the actions of CBD on anxiety and coping behaviors in mice. Pharmaceuticals (Basel). 2022;15:473.

8.	Kaplan JS, Wagner JK, Reid K, McGuinness F, Arvila S, Brooks M, et al. Cannabidiol exposure during the mouse adolescent period is without harmful behavioral effects on locomotor activity, anxiety, and spatial memory. Front Behav Neurosci. 2021;15:711639.

9.	Gasparyan A, Navarrete F, Manzanares J. Cannabidiol and sertraline regulate behavioral and brain gene expression alterations in an animal model of PTSD. Front Pharmacol. 2021;12:694510.

10.	Jin J, Fu C, Xia J, Luo H, Wang X, Chen S, et al. Cannabidiol ameliorates cognitive decline in 5×FAD mouse model of Alzheimer’s disease through potentiating the function of extrasynaptic glycine receptors. Mol Psychiatry. 2025;30:1817–1827.

11.	Silote GP, Gatto MC, Eskelund A, Guimarães FS, Wegener G, Joca SRL. Strain-, sex-, and time-dependent antidepressant-like effects of cannabidiol. Pharmaceuticals (Basel). 2021;14:1269.

12.	Abame MA, He Y, Wu S, Xie Z, Zhang J, Gong X, et al. Chronic administration of synthetic cannabidiol induces antidepressant effects involving modulation of serotonin and noradrenaline levels in the hippocampus. Neurosci Lett. 2021;744:135594.

13.	Aghamiri H, Jafari-Sabet M, Hoormand M. Ameliorative effect of cannabidiol on topiramate-induced memory loss: The role of hippocampal and prefrontal cortical NMDA receptors and CREB/BDNF signaling pathways in rats. Neurochem Res. 2024;49:363–378.

14.	Bitencourt RM, Pamplona FA, Takahashi RN. Facilitation of contextual fear memory extinction and anti-anxiogenic effects of AM404 and cannabidiol in conditioned rats. Eur Neuropsychopharmacol. 2008;18:849–859.

15.	Bright U, Akirav I. Cannabidiol modulates alterations in PFC microRNAs in a rat model of depression. Int J Mol Sci. 2023;24:2052.

16.	Campos AC, Guimarães FS. Involvement of 5HT1A receptors in the anxiolytic-like effects of cannabidiol injected into the dorsolateral periaqueductal gray of rats. Psychopharmacology (Berl). 2008;199:223–230.

17.	Campos AC, Ferreira FR, Guimarães FS. Cannabidiol blocks long-lasting behavioral consequences of predator threat stress: possible involvement of 5HT1A receptors. J Psychiatr Res. 2012;46:1501–1510.

18.	Campos AC, Brant F, Miranda AS, Machado FS, Teixeira AL. Cannabidiol increases survival and promotes rescue of cognitive function in a murine model of cerebral malaria. Neuroscience. 2015;289:166–180.

19.	Cassol-Jr OJ, Comim CM, Silva BR, Hermani FV, Constantino LS, Felisberto F, et al. Treatment with cannabidiol reverses oxidative stress parameters, cognitive impairment and mortality in rats submitted to sepsis by cecal ligation and puncture. Brain Res. 2010;1348:128–138.

20.	Chaves YC, Genaro K, Stern CA, de Oliveira Guaita G, de Souza Crippa JA, da Cunha JM, et al. Two-weeks treatment with cannabidiol improves biophysical and behavioral deficits associated with experimental type-1 diabetes. Neurosci Lett. 2020;729:135020.

21.	Chaves YC, Genaro K, Crippa JA, da Cunha JM, Zanoveli JM. Cannabidiol induces antidepressant and anxiolytic-like effects in experimental type-1 diabetic animals by multiple sites of action. Metab Brain Dis. 2021;36:639–652.

22.	Cheng D, Low JK, Logge W, Garner B, Karl T. Chronic cannabidiol treatment improves social and object recognition in double transgenic APPswe/PS1∆E9 mice. Psychopharmacology (Berl). 2014;231:3009–3017.

23.	Cheng D, Spiro AS, Jenner AM, Garner B, Karl T. Long-term cannabidiol treatment prevents the development of social recognition memory deficits in Alzheimer’s disease transgenic mice. J Alzheimers Dis. 2014;42:1383–1396.

24.	Chesworth R, Cheng D, Staub C, Karl T. Effect of long-term cannabidiol on learning and anxiety in a female Alzheimer’s disease mouse model. Front Pharmacol. 2022;13:931384.

25.	Coles M, Watt G, Kreilaus F, Karl T. Medium-dose chronic cannabidiol treatment reverses object recognition memory deficits of APP Swe /PS1ΔE9 transgenic female mice. Front Pharmacol. 2020;11:587604.

26.	Corrê M da S, de Freitas BS, Machado GDB, Pires VN, Bromberg E, Hallak JEC, et al. Cannabidiol reverses memory impairments and activates components of the Akt/GSK3β pathway in an experimental model of estrogen depletion. Behav Brain Res. 2022;417:113555.

27.	de Morais H, Chaves YC, Waltrick APF, Jesus CHA, Genaro K, Crippa JA, et al. Sub-chronic treatment with cannabidiol but not with URB597 induced a mild antidepressant-like effect in diabetic rats. Neurosci Lett. 2018;682:62–68.

28.	Ding Y-S, Wang J, Kumar V, Ciaccio J, Dakhel S, Tan C, et al. Evidence for cannabidiol modulation of serotonergic transmission in a model of osteoarthritis via in vivo PET imaging and behavioral assessment. Int J Innov Res Med Sci. 2022;7:254–271.

29.	Drummond-Main CD, Ahn Y, Kesler M, Gavrilovici C, Kim DY, Kiroski I, et al. Cannabidiol impairs brain mitochondrial metabolism and neuronal integrity. Cannabis Cannabinoid Res. 2023;8:283–298.

30.	Fabris D, Carvalho MC, Brandão ML, Prado WA, Zuardi AW, Crippa JA, et al. Sex-dependent differences in the anxiolytic-like effect of cannabidiol in the elevated plus-maze. J Psychopharmacol. 2022;36:1371–1383.

31.	de Paula Faria D, Estessi de Souza L, Duran FL de S, Buchpiguel CA, Britto LR, Crippa JA de S, et al. Cannabidiol treatment improves glucose metabolism and memory in streptozotocin-induced Alzheimer’s disease rat model: A proof-of-concept study. Int J Mol Sci. 2022;23:1076.

32.	Florensa-Zanuy E, Garro-Martínez E, Adell A, Castro E, Díaz Á, Pazos Á, et al. Cannabidiol antidepressant-like effect in the lipopolysaccharide model in mice: Modulation of inflammatory pathways. Biochem Pharmacol. 2021;185:114433.

33.	Fogaça MV, Reis FMCV, Campos AC, Guimarães FS. Effects of intra-prelimbic prefrontal cortex injection of cannabidiol on anxiety-like behavior: involvement of 5HT1A receptors and previous stressful experience. Eur Neuropsychopharmacol. 2014;24:410–419.

34.	Fogaça MV, Campos AC, Coelho LD, Duman RS, Guimarães FS. The anxiolytic effects of cannabidiol in chronically stressed mice are mediated by the endocannabinoid system: Role of neurogenesis and dendritic remodeling. Neuropharmacology. 2018;135:22–33.

35.	García-Baos A, Puig-Reyne X, García-Algar Ó, Valverde O. Cannabidiol attenuates cognitive deficits and neuroinflammation induced by early alcohol exposure in a mice model. Biomed Pharmacother. 2021;141:111813.

36.	Gasparyan A, Navarro D, Navarrete F, Austrich-Olivares A, Scoma ER, Hambardikar VD, et al. Cannabidiol repairs behavioral and brain disturbances in a model of fetal alcohol spectrum disorder. Pharmacol Res. 2023;188:106655.

37.	Georgieva-Kotetarova M, Kandilarov I, Vilmosh N, Zlatanova H, Yanchev N, Delev D, et al. Cannabidiol improves memory and decreases IL-1β serum levels in rats with lipopolysaccharide-induced inflammation. Folia Med (Plovdiv). 2023;65:940–949.

38.	Gomes FV, Llorente R, Del Bel EA, Viveros M-P, López-Gallardo M, Guimarães FS. Decreased glial reactivity could be involved in the antipsychotic-like effect of cannabidiol. Schizophr Res. 2015;164:155–163.

39.	Granjeiro EM, Gomes FV, Guimarães FS, Corrêa FMA, Resstel LBM. Effects of intracisternal administration of cannabidiol on the cardiovascular and behavioral responses to acute restraint stress. Pharmacol Biochem Behav. 2011;99:743–748.

40.	Greco R, Francavilla M, Demartini C, Zanaboni AM, Sodergren MH, Facchetti S, et al. Characterization of the biochemical and behavioral effects of cannabidiol: implications for migraine. J Headache Pain. 2023;24:48.

41.	De Gregorio D, McLaughlin RJ, Posa L, Ochoa-Sanchez R, Enns J, Lopez-Canul M, et al. Cannabidiol modulates serotonergic transmission and reverses both allodynia and anxiety-like behavior in a model of neuropathic pain. Pain. 2019;160:136–150.

42.	Guimarães FS, Chiaretti TM, Graeff FG, Zuardi AW. Antianxiety effect of cannabidiol in the elevated plus-maze. Psychopharmacology (Berl). 1990;100:558–559.

43.	Guimarães FS, de Aguiar JC, Mechoulam R, Breuer A. Anxiolytic effect of cannabidiol derivatives in the elevated plus-maze. Gen Pharmacol. 1994;25:161–164.

44.	Han X, Song X, Song D, Xie G, Guo H, Wu N, et al. Comparison between cannabidiol and sertraline for the modulation of post-traumatic stress disorder-like behaviors and fear memory in mice. Psychopharmacology (Berl). 2022;239:1605–1620.

45.	Hou M, Wang S, Yu D, Lu X, Zhao X, Chen Z, et al. Cannabidiol prevents depressive-like behaviors through the modulation of neural stem cell differentiation. Front Med. 2022;16:227–239.

46.	Kruk-Slomka M, Biala G. Cannabidiol attenuates MK-801-induced cognitive symptoms of schizophrenia in the passive avoidance test in mice. Molecules. 2021;26:5977.

47.	Linge R, Jiménez-Sánchez L, Campa L, Pilar-Cuéllar F, Vidal R, Pazos A, et al. Cannabidiol induces rapid-acting antidepressant-like effects and enhances cortical 5-HT/glutamate neurotransmission: role of 5-HT1A receptors. Neuropharmacology. 2016;103:16–26.

48.	Long LE, Chesworth R, Huang X-F, McGregor IS, Arnold JC, Karl T. A behavioural comparison of acute and chronic Delta9-tetrahydrocannabinol and cannabidiol in C57BL/6JArc mice. Int J Neuropsychopharmacol. 2010;13:861–876.

49.	Malvestio RB, Medeiros P, Negrini-Ferrari SE, Oliveira-Silva M, Medeiros AC, Padovan CM, et al. Cannabidiol in the prelimbic cortex modulates the comorbid condition between the chronic neuropathic pain and depression-like behaviour in rats: The role of medial prefrontal cortex 5-HT1A and CB1 receptors. Brain Res Bull. 2021;174:323–338.

50.	Marçal AP, Soares N, Asth L, Moreira FA, Ferreira AVM, Aguiar DC. Cannabidiol ameliorates the anxiogenic and compulsive-like behaviors induced by chronic consumption of a high-carbohydrate diet in male mice. Metab Brain Dis. 2022;37:2711–2718.

51.	Marinho ALZ, Vila-Verde C, Fogaça MV, Guimarães FS. Effects of intra-infralimbic prefrontal cortex injections of cannabidiol in the modulation of emotional behaviors in rats: contribution of 5HT₁A receptors and stressful experiences. Behav Brain Res. 2015;286:49–56.

52.	de Mattos BA, Bonato JM, Splendor MC, Del Bel E, Milani H, de Oliveira RMW. Cannabidiol improves non-motor symptoms, attenuates neuroinflammation, and favours hippocampal newborn neuronal maturation in a rat model of Parkinsonism. Acta Neuropsychiatr. 2024;36:307–319.

53.	Mesiakaris K, Atsopardi K, Lagoumintzis G, Margarity M, Poulas K. Cannabidiol modulation of nicotine-induced toxicity: Assessing effects on behavior, brain-derived neurotrophic factor, and oxidative stress in C57BL/6 male mice. J Neurosci Res. 2024;102:e25384.

54.	Meyer E, Bonato JM, Mori MA, Mattos BA, Guimarães FS, Milani H, et al. Cannabidiol confers neuroprotection in rats in a model of transient global cerebral ischemia: Impact of hippocampal synaptic neuroplasticity. Mol Neurobiol. 2021;58:5338–5355.

55.	Mori MA, Meyer E, Soares LM, Milani H, Guimarães FS, de Oliveira RMW. Cannabidiol reduces neuroinflammation and promotes neuroplasticity and functional recovery after brain ischemia. Prog Neuropsychopharmacol Biol Psychiatry. 2017;75:94–105.

56.	Mori MA, Meyer E, da Silva FF, Milani H, Guimarães FS, Oliveira RMW. Differential contribution of CB1, CB2, 5-HT1A, and PPAR-γ receptors to cannabidiol effects on ischemia-induced emotional and cognitive impairments. Eur J Neurosci. 2021;53:1738–1751.

57.	Myers AM, Siegele PB, Foss JD, Tuma RF, Ward SJ. Single and combined effects of plant-derived and synthetic cannabinoids on cognition and cannabinoid-associated withdrawal signs in mice. Br J Pharmacol. 2019;176:1552–1567.

58.	Osborne AL, Solowij N, Babic I, Huang X-F, Weston-Green K. Improved social interaction, recognition and working memory with cannabidiol treatment in a prenatal infection (poly I:C) rat model. Neuropsychopharmacology. 2017;42:1447–1457.

59.	Pazos MR, Cinquina V, Gómez A, Layunta R, Santos M, Fernández-Ruiz J, et al. Cannabidiol administration after hypoxia-ischemia to newborn rats reduces long-term brain injury and restores neurobehavioral function. Neuropharmacology. 2012;63:776–783.

60.	Poudel B, Bany BM, Hales DB, Cheatwood JL. Effects of cannabidiol (CBD) on Doxorubicin-induced anxiety and depression-like behaviors and mRNA expression of inflammatory markers in rats. Brain Sci. 2024;14:999.

61.	Raymundi AM, da Silva TR, Zampronio AR, Guimarães FS, Bertoglio LJ, Stern CAJ. A time-dependent contribution of hippocampal CB1 , CB2 and PPARγ receptors to cannabidiol-induced disruption of fear memory consolidation. Br J Pharmacol. 2020;177:945–957.

62.	Resstel LBM, Joca SRL, Moreira FA, Corrêa FMA, Guimarães FS. Effects of cannabidiol and diazepam on behavioral and cardiovascular responses induced by contextual conditioned fear in rats. Behav Brain Res. 2006;172:294–298.

63.	Réus GZ, Stringari RB, Ribeiro KF, Luft T, Abelaira HM, Fries GR, et al. Administration of cannabidiol and imipramine induces antidepressant-like effects in the forced swimming test and increases brain-derived neurotrophic factor levels in the rat amygdala. Acta Neuropsychiatr. 2011;23:241–248.

64.	da Silva Rodrigues F, Jantsch J, de Farias Fraga G, Luiza de Camargo Milczarski V, Silva Dias V, Scheid C, et al. Cannabidiol improves maternal obesity-induced behavioral, neuroinflammatory and neurochemical dysfunctions in the juvenile offspring. Brain Behav Immun. 2024;119:301–316.

65.	Rossignoli MT, Lopes-Aguiar C, Ruggiero RN, Do Val da Silva RA, Bueno-Junior LS, Kandratavicius L, et al. Selective post-training time window for memory consolidation interference of cannabidiol into the prefrontal cortex: Reduced dopaminergic modulation and immediate gene expression in limbic circuits. Neuroscience. 2017;350:85–93.

66.	Sales AJ, Crestani CC, Guimarães FS, Joca SRL. Antidepressant-like effect induced by Cannabidiol is dependent on brain serotonin levels. Prog Neuropsychopharmacol Biol Psychiatry. 2018;86:255–261.

67.	Sartim AG, Guimarães FS, Joca SRL. Antidepressant-like effect of cannabidiol injection into the ventral medial prefrontal cortex-Possible involvement of 5-HT1A and CB1 receptors. Behav Brain Res. 2016;303:218–227.

68.	Sartim AG, Sales AJ, Guimarães FS, Joca SR. Hippocampal mammalian target of rapamycin is implicated in stress-coping behavior induced by cannabidiol in the forced swim test. J Psychopharmacol. 2018;32:922–931.

69.	Sartim AG, Marques J, Silveira KM, Gobira PH, Guimarães FS, Wegener G, et al. Co-administration of cannabidiol and ketamine induces antidepressant-like effects devoid of hyperlocomotor side-effects. Neuropharmacology. 2021;195:108679.

70.	Schiavon AP, Soares LM, Bonato JM, Milani H, Guimarães FS, Weffort de Oliveira RM. Protective effects of cannabidiol against hippocampal cell death and cognitive impairment induced by bilateral common carotid artery occlusion in mice. Neurotox Res. 2014;26:307–316.

71.	Schiavon AP, Bonato JM, Milani H, Guimarães FS, Weffort de Oliveira RM. Influence of single and repeated cannabidiol administration on emotional behavior and markers of cell proliferation and neurogenesis in non-stressed mice. Prog Neuropsychopharmacol Biol Psychiatry. 2016;64:27–34.

72.	Shbiro L, Hen-Shoval D, Hazut N, Rapps K, Dar S, Zalsman G, et al. Effects of cannabidiol in males and females in two different rat models of depression. Physiol Behav. 2019;201:59–63.

73.	Shoval G, Shbiro L, Hershkovitz L, Hazut N, Zalsman G, Mechoulam R, et al. Prohedonic effect of cannabidiol in a rat model of depression. Neuropsychobiology. 2016;73:123–129.

74.	Silva-Cardoso GK, Lazarini-Lopes W, Hallak JE, Crippa JA, Zuardi AW, Garcia-Cairasco N, et al. Cannabidiol effectively reverses mechanical and thermal allodynia, hyperalgesia, and anxious behaviors in a neuropathic pain model: Possible role of CB1 and TRPV1 receptors. Neuropharmacology. 2021;197:108712.

75.	Sonego AB, Prado D da S, Guimarães FS. PPARγ receptors are involved in the effects of cannabidiol on orofacial dyskinesia and cognitive dysfunction induced by typical antipsychotic in mice. Prog Neuropsychopharmacol Biol Psychiatry. 2021;111:110367.

76.	Szkudlarek HJ, Desai SJ, Renard J, Pereira B, Norris C, Jobson CEL, et al. Δ-9-Tetrahydrocannabinol and Cannabidiol produce dissociable effects on prefrontal cortical executive function and regulation of affective behaviors. Neuropsychopharmacology. 2019;44:817–825.

77.	Tito PAL, Bernardino TC de S, Bellozi PMQ, da Silva MCM, de Miranda AS, Vieira ÉLM, et al. Cannabidiol prevents lipopolysaccharide-induced sickness behavior and alters cytokine and neurotrophic factor levels in the brain. Pharmacol Rep. 2021;73:1680–1693.

78.	Uhernik AL, Montoya ZT, Balkissoon CD, Smith JP. Learning and memory is modulated by cannabidiol when administered during trace fear-conditioning. Neurobiol Learn Mem. 2018;149:68–76.

79.	Villa M, Martínez-Vega M, Silva L, Muneta-Arrate I, Gómez-Soria A, Muguruza C, et al. Effects of cannabidiol in post-stroke mood disorders in neonatal rats. Pediatr Res. 2024;95:1783–1790.

80.	Watt G, Shang K, Zieba J, Olaya J, Li H, Garner B, et al. Chronic treatment with 50 mg/kg cannabidiol improves cognition and moderately reduces Aβ40 levels in 12-month-old male AβPPswe/PS1ΔE9 transgenic mice. J Alzheimers Dis. 2020;74:937–950.

81.	Watt G, Chesworth R, Przybyla M, Ittner A, Garner B, Ittner LM, et al. Chronic cannabidiol (CBD) treatment did not exhibit beneficial effects in 4-month-old male TAU58/2 transgenic mice. Pharmacol Biochem Behav. 2020;196:172970.

82.	Wolf SA, Bick-Sander A, Fabel K, Leal-Galicia P, Tauber S, Ramirez-Rodriguez G, et al. Cannabinoid receptor CB1 mediates baseline and activity-induced survival of new neurons in adult hippocampal neurogenesis. Cell Commun Signal. 2010;8:12.

83.	Xu C, Chang T, Du Y, Yu C, Tan X, Li X. Pharmacokinetics of oral and intravenous cannabidiol and its antidepressant-like effects in chronic mild stress mouse model. Environ Toxicol Pharmacol. 2019;70:103202.

84.	Zanelati TV, Biojone C, Moreira FA, Guimarães FS, Joca SRL. Antidepressant-like effects of cannabidiol in mice: possible involvement of 5-HT1A receptors: Cannabidiol induces antidepressant-like effect. Br J Pharmacol. 2010;159:122–128.

85.	Zieba J, Sinclair D, Sebree T, Bonn-Miller M, Gutterman D, Siegel S, et al. Cannabidiol (CBD) reduces anxiety-related behavior in mice via an FMRP-independent mechanism. Pharmacol Biochem Behav. 2019;181:93–100.

86.	Bis-Humbert C, García-Cabrerizo R, García-Fuster MJ. Antidepressant-like effects of cannabidiol in a rat model of early-life stress with or without adolescent cocaine exposure. Pharmacol Rep. 2021;73:1195–1202.

87.	Bis-Humbert C, García-Cabrerizo R, García-Fuster MJ. Decreased sensitivity in adolescent versus adult rats to the antidepressant-like effects of cannabidiol. Psychopharmacology (Berl). 2020;237:1621–1631.

88.	Gáll Z, Farkas S, Albert Á, Ferencz E, Vancea S, Urkon M, et al. Effects of chronic cannabidiol treatment in the rat chronic unpredictable mild stress model of depression. Biomolecules. 2020;10:801.

89.	Amini M, Abdolmaleki Z. The effect of cannabidiol coated by nano-chitosan on learning and memory, hippocampal CB1 and CB2 levels, and amyloid plaques in an Alzheimer’s disease rat model. Neuropsychobiology. 2022;81:171–183.

90.	Rêgo DSB, Calió ML, Filev R, Mello LE, Leslie ATFS. Long-term effects of cannabidiol and/or fentanyl exposure in rats submitted to neonatal pain. J Pain. 2024;25:715–729.

91.	Li M, Xu B, Li X, Li Y, Qiu S, Chen K, et al. Mitofusin 2 confers the suppression of microglial activation by cannabidiol: Insights from in vitro and in vivo models. Brain Behav Immun. 2022;104:155–170.

92.	Wang L, Wu X, Yang G, Hu N, Zhao Z, Zhao L, et al. Cannabidiol alleviates the damage to dopaminergic neurons in 1-methyl-4-phenyl-1,2,3,6-tetrahydropyridine-induced Parkinson’s disease mice via regulating neuronal apoptosis and neuroinflammation. Neuroscience. 2022;498:64–72.

93.	Rodrigues F da S, Jantsch J, de Farias Fraga G, Dias VS, Pereira Medeiros C, Wickert F, et al. Cannabidiol partially rescues behavioral, neuroinflammatory and endocannabinoid dysfunctions stemming from maternal obesity in the adult offspring. Neuropharmacology. 2025;262:110196.

94.	Shen Z, Bao N, Chen J, Tang M, Yang L, Yang Y, et al. Neuromolecular and behavioral effects of cannabidiol on depressive-associated behaviors and neuropathic pain conditions in mice. Neuropharmacology. 2024;261:110153.

95.	Domingos LB, Müller HK, da Silva NR, Filiou MD, Nielsen AL, Guimarães FS, et al. Repeated cannabidiol treatment affects neuroplasticity and endocannabinoid signaling in the prefrontal cortex of the Flinders Sensitive Line (FSL) rat model of depression. Neuropharmacology. 2024;248:109870.

96.	Frederico Gava F, Jaconi De Carli R, Stork S, Gainski Danielski L, Bonfante S, Joaquim L, et al. Cannabidiol effect on long-term brain alterations in septic rats: Involvement of PPARγ activation. Brain Res. 2024;1828:148771.

97.	Franzen JM, Vanz F, Werle I, Guimarães FS, Bertoglio LJ. Cannabidiol impairs fear memory reconsolidation in female rats through dorsal hippocampus CB1 but not CB2 receptor interaction. Eur Neuropsychopharmacol. 2022;64:7–18.

98.	Alonso C, Satta V, Hernández-Fisac I, Fernández-Ruiz J, Sagredo O. Disease-modifying effects of cannabidiol, β-caryophyllene and their combination in Syn1-Cre/Scn1aWT/A1783V mice, a preclinical model of Dravet syndrome. Neuropharmacology. 2023;237:109602.

99.	Alvarez FJ, Alvarez AA, Rodríguez JJ, Lafuente H, Canduela MJ, Hind W, et al. Effects of cannabidiol, hypothermia, and their combination in newborn rats with hypoxic-ischemic encephalopathy. eNeuro. 2023;10.

100.	Aso E, Fernández-Dueñas V, López-Cano M, Taura J, Watanabe M, Ferrer I, et al. Adenosine A2A-cannabinoid CB1 receptor heteromers in the hippocampus: Cannabidiol blunts Δ9-tetrahydrocannabinol-induced cognitive impairment. Mol Neurobiol. 2019;56:5382–5391.

101.	Cabral-Pereira G, Sánchez-Benito D, Díaz-Rodríguez SM, Gonçalves J, Sancho C, Castellano O, et al. Behavioral and molecular effects induced by cannabidiol and valproate administration in the GASH/Sal model of acute audiogenic seizures. Front Behav Neurosci. 2020;14:612624.

102.	Chaves YC, Raymundi AM, Waltrick APF, de Souza Crippa JA, Stern CAJ, da Cunha JM, et al. Cannabidiol modulates contextual fear memory consolidation in animals with experimentally induced type-1 diabetes mellitus. Acta Neuropsychiatr. 2024;36:276–286.

103.	El-Alfy AT, Ivey K, Robinson K, Ahmed S, Radwan M, Slade D, et al. Antidepressant-like effect of Δ9-tetrahydrocannabinol and other cannabinoids isolated from Cannabis sativa L. Pharmacol Biochem Behav. 2010;95:434–442.

104.	Gáll Z, Kelemen K, Tolokán A, Zolcseak I, Sável I, Bod R, et al. Anticonvulsant action and long-term effects of chronic cannabidiol treatment in the rat pentylenetetrazole-kindling model of epilepsy. Biomedicines. 2022;10:1811.

105.	Kodali M, Madhu LN, Kolla VSV, Attaluri S, Huard C, Somayaji Y, et al. FDA-approved cannabidiol [Epidiolex®] alleviates Gulf War Illness-linked cognitive and mood dysfunction, hyperalgesia, neuroinflammatory signaling, and declined neurogenesis. Mil Med Res. 2024;11:61.

106.	Ledesma-Corvi S, Hernández-Hernández E, García-Fuster MJ. Exploring pharmacological options for adolescent depression: a preclinical evaluation with a sex perspective. Transl Psychiatry. 2022;12:220.

107.	Macêdo-Souza C, Maisonnette SS, Hallak JE, Crippa JA, Zuardi AW, Landeira-Fernandez J, et al. Systemic chronic treatment with cannabidiol in Carioca high- and low-conditioned freezing rats in the neuropathic pain model: Evaluation of pain sensitivity. Pharmaceuticals (Basel). 2023;16:1003.

108.	Pérez-Valenzuela E, Hudson R, Uzuneser T, De Felice M, Szkudlarek H, Rushlow W, et al. Sex-dependent synergism of an edible THC: CBD formulation in reducing anxiety and depressive-like symptoms following chronic stress. Curr Neuropharmacol. 2024;22:2059–2078.

109.	Resstel LBM, Tavares RF, Lisboa SFS, Joca SRL, Corrêa FMA, Guimarães FS. 5-HT1A receptors are involved in the cannabidiol-induced attenuation of behavioural and cardiovascular responses to acute restraint stress in rats. Br J Pharmacol. 2009;156:181–188.

110.	Silvestri C, Pagano E, Lacroix S, Venneri T, Cristiano C, Calignano A, et al. Fish oil, cannabidiol and the gut Microbiota: An investigation in a Murine model of colitis. Front Pharmacol. 2020;11:585096.

111.	Stollenwerk TM, Pollock S, Hillard CJ. Contribution of the adenosine 2A receptor to behavioral effects of tetrahydrocannabinol, cannabidiol and PECS-101. Molecules. 2021;26:5354.

112.	Visini G, Brown S, Weston-Green K, Shannon Weickert C, Chesworth R, Karl T. The effects of preventative cannabidiol in a male neuregulin 1 mouse model of schizophrenia. Front Cell Neurosci. 2022;16:1010478.

113.	Xie G, Qin Y, Wu N, Han X, Li J. Single-nucleus transcriptome profiling from the hippocampus of a PTSD mouse model and CBD-treated cohorts. Genes (Basel). 2024;15:519.

114.	Assareh N, Gururajan A, Zhou C, Luo JL, Kevin RC, Arnold JC. Cannabidiol disrupts conditioned fear expression and cannabidiolic acid reduces trauma-induced anxiety-related behaviour in mice. Behav Pharmacol. 2020;31:591–596.

115.	Gomes FV, Reis DG, Alves FHF, Corrêa FMA, Guimarães FS, Resstel LBM. Cannabidiol injected into the bed nucleus of the stria terminalis reduces the expression of contextual fear conditioning via 5-HT1A receptors. J Psychopharmacol. 2012;26:104–113.

116.	Luján MÁ, Cantacorps L, Valverde O. The pharmacological reduction of hippocampal neurogenesis attenuates the protective effects of cannabidiol on cocaine voluntary intake. Addict Biol. 2020;25:e12778.

117.	Martín-Sánchez A, González-Pardo H, Alegre-Zurano L, Castro-Zavala A, López-Taboada I, Valverde O, et al. Early-life stress induces emotional and molecular alterations in female mice that are partially reversed by cannabidiol. Prog Neuropsychopharmacol Biol Psychiatry. 2022;115:110508.

118.	Sales AJ, Guimarães FS, Joca SRL. CBD modulates DNA methylation in the prefrontal cortex and hippocampus of mice exposed to forced swim. Behav Brain Res. 2020;388:112627.

119.	Ribeiro de Novais Júnior L, Vicente da Silva T, da Silva LM, Metzker de Andrade F, da Silva AR, Meneguzzo V, et al. Repeated administration of a full-spectrum cannabidiol product, not a cannabidiol isolate, reverses the lipopolysaccharide-induced depressive-like behavior and hypolocomotion in a rat model of low-grade subchronic inflammation. Cannabis Cannabinoid Res. 2025;10:236–246.

120.	Arnanz MA, Ruiz de Martín Esteban S, Martínez Relimpio AM, Rimmerman N, Tweezer Zaks N, Grande MT, et al. Effects of chronic, low-dose cannabinoids, cannabidiol, delta-9-tetrahydrocannabinol and a combination of both, on amyloid pathology in the 5xFAD mouse model of Alzheimer’s disease. Cannabis Cannabinoid Res. 2024;9:1312–1325.

121.	Dinur E, Goldenberg H, Robinson E, Naggan L, Kozela E, Yirmiya R. A novel anti-inflammatory formulation comprising celecoxib and cannabidiol exerts antidepressant and anxiolytic effects. Cannabis Cannabinoid Res. 2024;9:561–580.

122.	Dong S, Zhao H, Nie M, Sha Z, Feng J, Liu M, et al. Cannabidiol alleviates neurological deficits after traumatic brain injury by improving intracranial lymphatic drainage. J Neurotrauma. 2024;41:e2009–e2025.

123.	Sturaro C, Fakhoury B, Targowska-Duda KM, Zribi G, Schoch J, Ruzza C, et al. Preclinical effects of cannabidiol in an experimental model of migraine. Pain. 2023;164:2540–2552.
